# Recognition and Degradation of Plant Cell Wall Polysaccharides by Two Human Gut Symbionts

**DOI:** 10.1371/journal.pbio.1001221

**Published:** 2011-12-20

**Authors:** Eric C. Martens, Elisabeth C. Lowe, Herbert Chiang, Nicholas A. Pudlo, Meng Wu, Nathan P. McNulty, D. Wade Abbott, Bernard Henrissat, Harry J. Gilbert, David N. Bolam, Jeffrey I. Gordon

**Affiliations:** 1 Center for Genome Sciences and Systems Biology, Washington University School of Medicine, St. Louis, Missouri, United States of America; 2 Department of Microbiology and Immunology, University of Michigan Medical School, Ann Arbor, Michigan, United States of America; 3 Institute for Cell and Molecular Biosciences, The Medical School, Newcastle University, Newcastle upon Tyne, United Kingdom; 4 Complex Carbohydrate Research Center, University of Georgia, Athens, Georgia, United States of America; 5 Architecture et Fonction des Macromolécules Biologiques, CNRS and Universities of Aix-Marseille I & II, Marseille, France; University of California Davis, United States of America

## Abstract

Symbiotic bacteria inhabiting the human gut have evolved under intense pressure to utilize complex carbohydrates, primarily plant cell wall glycans in our diets. These polysaccharides are not digested by human enzymes, but are processed to absorbable short chain fatty acids by gut bacteria. The Bacteroidetes, one of two dominant bacterial phyla in the adult gut, possess broad glycan-degrading abilities. These species use a series of membrane protein complexes, termed Sus-like systems, for catabolism of many complex carbohydrates. However, the role of these systems in degrading the chemically diverse repertoire of plant cell wall glycans remains unknown. Here we show that two closely related human gut Bacteroides, *B. thetaiotaomicron* and *B. ovatus*, are capable of utilizing nearly all of the major plant and host glycans, including rhamnogalacturonan II, a highly complex polymer thought to be recalcitrant to microbial degradation. Transcriptional profiling and gene inactivation experiments revealed the identity and specificity of the polysaccharide utilization loci (PULs) that encode individual Sus-like systems that target various plant polysaccharides. Comparative genomic analysis indicated that *B. ovatus* possesses several unique PULs that enable degradation of hemicellulosic polysaccharides, a phenotype absent from *B. thetaiotaomicron*. In contrast, the *B. thetaiotaomicron* genome has been shaped by increased numbers of PULs involved in metabolism of host mucin *O*-glycans, a phenotype that is undetectable in *B. ovatus*. Binding studies of the purified sensor domains of PUL-associated hybrid two-component systems in conjunction with transcriptional analyses demonstrate that complex oligosaccharides provide the regulatory cues that induce PUL activation and that each PUL is highly specific for a defined cell wall polymer. These results provide a view of how these species have diverged into different carbohydrate niches by evolving genes that target unique suites of available polysaccharides, a theme that likely applies to disparate bacteria from the gut and other habitats.

## Abstract 2

Competition for nutrients contained in diverse types of plant cell wall-associated polysaccharides may explain the evolution of substrate-specific catabolic gene modules in common bacterial members of the human gut microbiota.

## Author Summary

Bacteria inhabiting the human gut are critical for digestion of the plant-derived glycans that compose dietary fiber. Enzymes produced by the human body cannot degrade these abundant dietary components, and without bacterial assistance they would go unused. We investigated the molecular strategies employed by two species belonging to one of the most abundant bacterial groups in the human colon (the Bacteroidetes). Our results show that each species has evolved to degrade a unique subset of glycans; this specialization is reflected in their respective genomes, each of which contains numerous separate gene clusters involved in metabolizing plant fiber polysaccharides or glycans present in secreted mucus. Each glycan-specific gene cluster produces a related series of membrane-associated proteins which together serve to bind and degrade a specific glycan. Expression of each glycan-specific gene cluster is controlled by an environmental sensor that responds to the presence of a unique molecular signature contained in the substrate that it targets. These results provide a view of how related bacterial species have diverged into different carbohydrate niches by evolving genes that sense and degrade unique suites of available polysaccharides, a process that likely applies to disparate bacteria from the gut and other habitats.

## Introduction

The human distal gut is home to a densely populated microbial community (microbiota) that plays key roles in health and nutrition. The microbial symbionts that occupy this habitat produce an arsenal of enzymes that degrade dietary complex carbohydrates (glycans) that cannot be hydrolyzed by host enzymes [1]. The simple sugars generated are fermented into host-absorbable end products, including short-chain fatty acids, that can contribute as much as ∼10% of the calories extracted from the human diet and are thought to play a role in preventing colorectal cancer [2].

Viewed at the broad taxonomic level of bacterial phylum, two groups of bacteria dominate the distal gut microbiota of adult humans and other mammals: the Bacteroidetes and the Firmicutes [3]–[6]. Studies of cultivated human microbiota species indicate that the Bacteroidetes, composed largely of members of the genus *Bacteroides*, exhibit broad capacities to metabolize a variety of plant- and animal-derived glycans [7],[8].

Bacteroidetes from a variety of environments including the human gut employ a similar strategy for binding and degrading various glycans [9]. These Gram-negative bacteria have amplified and permuted a series of gene clusters termed polysaccharide utilization loci (PULs). Each PUL that has been characterized to date encodes a suite of cell envelope-associated proteins (Sus-like system) that confer the ability to metabolize a single glycan or group of related glycans [8],[10]–[12]. Each Sus-like system contains at least one pair of outer membrane proteins homologous to SusC and SusD, which are essential for the import and degradation of starch by the prototypic starch-utilization system (Sus) for which related systems are named [10]. SusC-like proteins are predicted TonB-dependent receptors that span the outer membrane and transport oligosaccharides in an energy-dependent manner. SusD-like proteins are outer membrane lipoproteins that are oriented towards the external environment; they bind directly to specific glycans and contribute to the capture and delivery of oligosaccharides to the SusC transporter [12],[13]. SusC- and SusD-like proteins function in concert with other outer membrane glycan binding proteins and polysaccharide degrading enzymes (glycoside hydrolases, polysaccharide lyases, and carbohydrate esterases), which are grouped into sequence-based families in the Carbohydrate Active Enzymes (CAZy) database [14].

PULs frequently contain genes encoding inner membrane sensor-regulator systems that control the expression of genes in their associated and usually adjacent locus [12],[15],[16]. These sensor-regulators are most commonly extra-cytoplasmic function sigma (ECF-σ)/anti-σ factor pairs or hybrid two-component systems (HTCS) that contain all of the domains of a classical two-component system phosphorelay in a single polypeptide [17]. While glycans are known to activate PUL-associated HTCS, there is a paucity of information about the actual molecular cues recognized, and the mechanism by which these inducers mediate their effect, although a previous study has indicated that large oligosaccharides are involved in activation of an HTCS controlled xylanase locus from a ruminant Bacteroidetes [18]. In the simplest model, the activating glycan will bind directly to the periplasmic sensor domain, as occurs when fructose activates the *B. thetaiotaomicron* fructan PUL by binding directly to its HTCS [12]. However, the periplasmic sensor domain of the fructan HTCS is atypical compared to those found in other HTCS (i.e., it is around one-third the size and adopts a periplasmic binding protein fold). It is therefore possible that the sensor domains most commonly associated with other PULs recognize more complex ligands, that the glycans are presented to the cognate HTCS bound to other periplasmic proteins, or that the HTCS interact with part of their cognate SusC porin in the periplasm in a manner analogous to the trans-envelope signaling that occurs in ECF-σ/anti-σ systems [16],[19]. Furthermore, the extent to which there is cross-talk between the regulatory systems of different PULs is unclear. Thus, while previous animal feeding experiments have shown which PULs in *B. thetaiotaomicron* are activated in vivo by diets containing plant polysaccharides [20],[21], we do not know the specific components of the diet that activate individual PULs.

A major factor shaping the balance between different human gut bacterial phylogenetic types (phylotypes) is the ability of each group to compete efficiently for the complex glycans that are delivered to the distal intestine. To understand how human gut bacteria have evolved to occupy distinct niches, we have measured the ability of two closely related *Bacteroides* phylotypes to metabolize complex dietary glycans. *B. thetaiotaomicron* and *B. ovatus* share 96.5% nucleotide sequence identity in their 16S rDNA genes. With the exception of cellulose, these species are together capable of using all major glycan classes found in the gut mucosa and in plant cells as sole carbon sources. However, each species alone has evolved to target only a partial set of all possible glycans. We use transcriptional profiling, in conjunction with the characterization of mutants lacking functional PULs, to establish that their ability to metabolize different plant cell wall glycans is contingent on selective expression of PUL-encoded Sus-like systems. Most of the identified plant glycan PULs are linked to an HTCS that is activated by the presence of the PUL's target glycan. Specific activation of each PUL is achieved by direct recognition of signature oligosaccharide cues by the cognate HTCS. *B. thetaiotaomicron* contains many PULs that are not present in *B. ovatus* and confer its increased ability to target host mucin *O*-glycans and expanded capacity to target pectic structures. Conversely, *B. ovatus* harbors several unique PULs that enable it to use all of the common hemicelluloses, while *B. thetaiotaomicron* is unable to metabolize this group of plant structural polysaccharides. In both cases, unique species-specific PULs are scattered throughout the bacterial genome rather than being present in one or more large blocks. These data support the concept that adaptation to different glycan niches is driving selective evolution of PULs in these two species. This theme may apply to other bacteria in the human gut and has implications for both the basic ecology of the gut microbiota as well as efforts to intentionally manipulate this community to restore health or alter nutrition.

## Results and Discussion

### *B. thetaiotaomicron* and *B. ovatus* Have Distinct But Partially Overlapping Glycan Niches

To investigate the relationship between glycan metabolic phenotypes and underlying genetic architecture in commonly isolated human gut bacteria, we focused on *B. thetaiotaomicron* ATCC 29148 (also known as VPI-5482) and *B. ovatus* ATCC 8483. The rationale for our selection is based on a previous survey showing that while members of these two species can access a wide range of glycans [7], they display substantial differences in complex carbohydrate utilization.

To define in detail the range of glycans that *B. thetaiotaomicron* and *B. ovatus* are capable of utilizing, we constructed a custom panel of plant, animal, and microbial carbohydrates arrayed in 96-well format (Table 1). These polysaccharides are more extensive than those used in previous studies, and include plant polymers such as rhamnogalacturonan I and II and purified fragments from a number of other highly decorated pectins and hemicelluloses. In addition, we monitored anaerobic growth over a defined time interval, enabling quantitative measurements of both growth rate and final culture density for each glycan.

**Table 1:** Growth of *B. thetaiotaomicron (Bt)* and *B. ovatus (Bo)* on glycans and monosaccharides.

Substrate (Source)	*Bt* Rate (ΔA_600_/h)	*Bt* Density (A_600_ max)	*Bo* Rate (ΔA_600_/h)	*Bo* Density (A_600_ max)
**Pectins:**				
arabinan (sugar beet)^b^	0.037±0.003^a^	0.56±0.02^a^	0.007±0.002	0.13±0.06
arabinogalactan (larch)^b^	0.045±0.007^a^	0.60±0.04^a^	no growth	no growth
pectic galactan (potato)^b^	0.093±0.013^a^	0.82±0.10	0.057±0.012	0.75±0.17
homogalacturonan (citrus)^b^^,^ c	0.066±0.013^a^	0.70±0.13^a^	0.042±0.008	0.55±0.06
rhamnogalacturonan I (potato)^b^	0.027±0.004	0.54±0.04	0.036±0.005^a^	0.65±0.06^a^
rhamnogalacturonan II (red wine)^b^	0.033±0.005	0.50±0.04	0.040±0.003	0.61±0.01^a^
**Hemicelluloses and β-glucans:**				
arabinoxylan (wheat)^c^	no growth	no growth	0.030±0.016^a^	0.63±0.12^a^
xylan, water soluble (oat)^c^	no growth	no growth	0.024±0.009^a^	0.65±0.09^a^
xyloglucan (tamarind)^c^	no growth	no growth	0.065±0.021^a^	0.75±0.11^a^
glucomannan (konjac)^c^	no growth	no growth	0.027±0.003^a^	0.27±0.05^a^
galactomannan (carob)^c^	no growth	no growth	0.105±0.027^a^	0.94±0.04^a^
β-glucan (barley)^c^	no growth	no growth	0.028±0.014^a^	0.67±0.12^a^
lichenin (Icelandic moss)	no growth	no growth	no growth	no growth
laminarin (seaweed)	no growth	no growth	no growth	no growth
**Cellooligosaccharides:**				
cellobiose	no growth	no growth	0.020±0.012^a^	0.70±0.32^a^
cellohexaose	no growth	no growth	no growth	no growth
**Starch, fructan, and α-glucans:**				
amylopectin (potato)	0.075±0.007	0.96±0.04	0.101±0.014^a^	1.12±0.09^a^
amylopectin (maize)	0.056±0.005	0.89±0.05	0.086±0.017^a^	0.97±0.07
pullulan^b^	0.063±0.004	0.82±0.05	0.068±0.039	0.76±0.20
dextran	0.108±0.017	1.07±0.05	0.114±0.028	1.15±0.09
inulin (chicory)	0.005±0.002	0.25±0.07	0.026±0.004^a^	0.59±0.05^a^
levan	0.036±0.007^a^	0.52±0.04^a^	no growth	no growth
**Host-derived glycans:**				
neutral mucin *O*-glycans^b^	0.015±0.002^a^	0.29±0.01^a^	no growth	no growth
α-mannan^b^	0.035±0.003^a^	0.58±0.02^a^	0.004±0.001	0.16±0.01
chondroitin sulfate^b^	0.065±0.009^a^	0.64±0.05^a^	0.013±0.002	0.40±0.02
hyaluronan^b^	0.125±0.019^a^	1.08±0.18	0.053±0.018	0.76±0.29
heparin^b^	0.025±0.006	0.31±0.04^a^	0.016±0.007	0.20±0.01
glycogen	0.050±0.003	0.93±0.02	0.054±0.018	0.87±0.06
**Monosaccharides:**				
arabinose	0.031±0.004	0.28±0.04	0.033±0.021	0.43±0.19
fructose	0.073±0.006	0.99±0.03	0.048±0.005	0.88±0.04
fucose	0.024±0.002	0.54±0.04	0.024±0.008	0.39±0.08
galactose	0.038±0.003	0.42±0.03	0.041±0.029	0.75±0.41
galacturonic acid	0.021±0.002	0.62±0.02	0.010±0.003	0.57±0.08
glucose^b^^,^ c	0.067±0.013	0.81±0.13	0.084±0.026	0.89±0.20
glucuronic acid	0.024±0.005	0.57±0.02	0.029±0.016	0.67±0.17
glucosamine	0.008±0.001	0.42±0.03	0.031±0.002	0.77±0.15
mannose	0.103±0.006	1.24±0.05	0.061±0.041	0.95±0.12
N-acetylgalactosamine	0.007±0.0003	0.43±0.02	0.009±0.005	0.62±0.31
N-acetylglucosamine	0.066±0.007	1.10±0.12	0.029±0.006	0.84±0.12
N-acetylneuraminic acid	no growth	no growth	no growth	no growth
rhamnose	0.033±0.016	0.48±0.17	0.023±0.010	0.35±±0.11
ribose	0.040±0.003	0.52±0.03	0.049±±0.020	0.78±0.24
xylose	0.049±0.005	0.50±0.04	0.047±0.027	0.80±0.34

Growth measurements are described in Materials and Methods; values in superscript represent one standard deviation of 6 replicate measurements.

a Glycans for which the growth rate or density of one strain was significantly greater than the other (*p* value<0.01; Student's *t* test).

b Substrates on which *Bt* was transcriptionally profiled.

c Substrates on which *Bo* was transcriptionally profiled.

*B. thetaiotaomicron* and *B. ovatus* each grew on a subset of the glycans tested (Table 1). Both species grew on plant cell wall pectins except arabinogalactan and arabinan, which were only efficiently utilized by *B. thetaiotaomicron*. Interestingly, as we had noted during a previous comparison of *B. thetaiotaomicron* growth on starch and dextran [13], each species grew more rapidly on several different polysaccharides compared to their corresponding monosaccharide components. Although this result is counter-intuitive because each polysaccharide needs to first be de-polymerized prior to metabolism, it suggests that monosaccharide transport and metabolism pathways are optimally triggered by the presence of polymerized sugar molecules. For example, growth of *B. thetaiotaomicron* on potato pectic galactan was 2.5±0.5 times more rapid than on its monosaccharide constituent galactose (*p*<0.001, Student's *t* test). More efficient utilization of polymeric glycans may be attributable to the fact that very little free monosaccharide reaches *Bacteroides* species in the distal intestine, with selective pressures acting on these bacteria to evolve ways to directly couple downstream catabolic pathways to glycan recognition.

*B. ovatus* was the only one of the two species capable of growing on the hemicelluloses tested (Table 1); it also grew on the disaccharide cellobiose but not on cellohexaose, suggesting that it is not capable of targeting higher molecular forms of cellulose. We conclude that the ability of *B. ovatus* to grow on cellobiose is a product of its ability to degrade other molecules that contain β1,4-glucosidic linkages, such as barley β-glucan or xyloglucan, a view supported by HTCS specificity data presented below. Additional insight into the sensing and catabolic specificities of *B. ovatus* for closely related β-glucan substrates was provided by testing a variety of structures that vary with respect to the linkages they contain and their relative ratios in the polysaccharide (see Text S1).

Beyond their utilization of plant cell wall glycans, both species grew well on plant cell storage carbohydrates such as fructans and starch (amylopectin), and on the starch-like molecules pullulan and glycogen. *B. thetaiotaomicron* and *B. ovatus* have reciprocal specificities for fructans: *B. thetaiotaomicron* grows best on the β2,6-linkages that occur in levan, whereas *B. ovatus* does not grow at all on levan but prefers β2,1-linked inulin [12].

Only *B. thetaiotaomicron* grew on mucin *O*-glycans, a trait that we previously demonstrated to be dependent on expression of over a dozen different PULs [8]. *B. thetaiotaomicron* also grew much more efficiently than *B. ovatus* on α-mannan, a fungal cell wall glycan that contains similar α-mannosidic linkages to those found in the core regions of *N*-linked glycans present on secreted mucus and epithelial surfaces. Lastly, *B. thetaiotaomicron* exhibited better growth (more rapid rate and higher cell density) on the third class of host-derived glycans, glycosaminoglycans (GAGs), which include chondroitin sulfate, heparin, and hyaluronan.

Together, the results indicate that these two human gut-associated *Bacteroides* have evolved distinct and only partially overlapping glycan niches. *B. thetaiotaomicron* is more adept at foraging the more soluble, and possibly more accessible, pectic components of plant cell walls. It also exhibits a well-developed capacity to metabolize host mucin *O*-glycans, a trait that could allow it to preferentially colonize the protective mucus layer that overlies the gut epithelium. Conversely, *B. ovatus* is metabolically specialized to utilize less soluble plant cell wall components like hemicelluloses, in addition to pectins, and thus is more likely to occupy physical microhabitats located in the gut lumen.

### *B. thetaiotaomicron* Deploys a Subset of Its Sus-Like Systems to Target Cell Wall Pectins

To examine the molecular basis underlying plant cell wall glycan utilization by these two species, we used custom GeneChips representing 99.5% and all of the predicted or known ORFs in the *B. thetaiotaomicron* and *B. ovatus* genomes, respectively. Our rationale was that identifying the genes responsible for plant cell wall degradation would allow us to compare the genomic location and organization of these genes between species. Whole-genome transcriptional profiles were generated for each species during exponential growth on individual glycans (substrates upon which each species was profiled are noted in Table 1). The specific transcriptional responses of each species to growth on a particular glycan were then determined by comparison to a reference dataset of that same species grown in minimal medium with glucose as the sole carbon source (MM-G).

We previously demonstrated that genes associated with individual PULs typically exhibit large increases in their transcription when exposed to the substrates they process [8],[12]. Therefore, we applied a cutoff of ≥10-fold change in expression in minimal medium containing a given glycan compared to MM-G. A total of 280 *B. thetaiotaomicron* genes exhibited altered expression in response to growth on one or more of the six pectins tested, or pullulan (a control for expression of the starch utilization system). Expression of 268 genes (96%) was increased, while only 12 exhibited decreased expression (see Table S1 for a list of genes and fold-change values). Of the genes with altered expression during growth on one or more glycans, 155 (56%) were associated with 16 different PULs and all but five of these genes were upregulated. These observations indicate that PULs are a primary component of this symbiont's response to different pectins.

**Figure 1:**
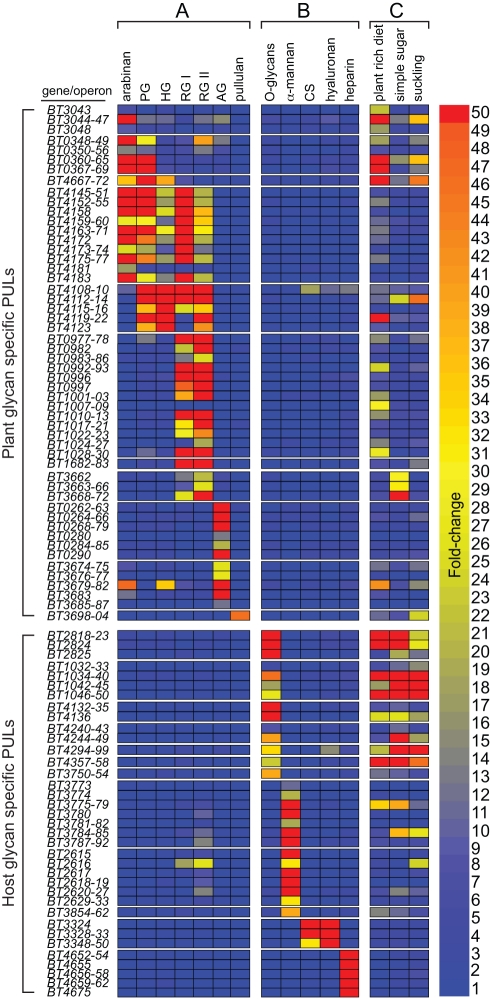
*B. thetaiotaomicron* PULs expressed in response to plant and host glycans. Heatmap showing PUL operon induction by various plant and host glycans. Each box represents the average fold-change (relative to MM-G) of all genes within the indicated operon (gene numbers listed at left) and is calibrated according to the vertical bar at the extreme right. Individual PULs are separated by horizontal breaks in the heat map. Growth conditions have been divided into three categories: column A, growth on plant cell wall pectins and the starch-like molecule pullulan; column B, growth on different forms of host glycans or α-mannan (a proxy for the mannose rich core region of *N*-glycans); column C, growth in vivo in adult mono-associated NMRI mice fed a “plant rich diet” or a “simple sugar” diet, or neonatal mice that are still suckling on mother's milk (“suckling”). A Venn diagram illustration of total gene changes in these conditions is provided in Figure S3. PG, pectic galactan; HG, homogalacturonan; RG I, rhamnogalacturonan I; RG II, rhamnogalacturonan II; AG, arabinogalactan; CS, chondroitin sulfate.

To better visualize responses of entire PULs to the various glycans tested, genes were grouped into putative operons [22] and the average fold-change of each operon re-calculated from normalized GeneChip values [8]. Eleven PULs had one or more operons that still exhibited ≥10-fold induction when cells were exposed to pectin or pullulan (Figure 1). Based on the predicted activities of the enzymes encoded by these PULs (enzymes in CAZy families where pectin degradation is a common feature), it is likely that 10 of them (all except the pullulan-induced starch PUL, *BT3698-3704*) make a significant contribution to pectin degradation (Table S2; for schematic diagrams of these 10 PULs, see Figure S1). Support for this view is provided by a recent study that showed that the three GH43 enzymes from the arabinan-activated PUL spanning *BT0348-69* display arabinan-specific activity [23]. This emerging portrait of the predicted enzyme specificity in PULs that have been empirically matched with specific polysaccharide substrates will provide a valuable template for future functional annotation of the more than 100 Bacteroidetes genomes that are known to harbor similar gene clusters, as well as a starting point for more focused biochemical and enzymatic studies of how these systems each attack their specific substrates.

Comparison of our data to previous in vivo studies using gnotobiotic mice colonized with *B. thetaiotaomicron* alone (“mono-associated”) showed that a subset of the pectin-responsive loci were expressed in the ceca of animals consuming a diet composed of wheat- and soy-based plant material (“plant-rich diet” column in Figure 1), suggesting that these natural dietary substrates contain pectins that are accessible to *B. thetaiotaomicron* in the absence of other bacteria. When these dietary substrates are withheld, as in mice fed a simple sugar diet [20] or in neonatal mice suckling on mother's milk [21], expression of the “pectin PULs” is reduced. In contrast, PULs targeting host-derived glycans, especially the *O*-glycans that are abundant in secreted mucus, are highly expressed regardless of diet, suggesting that *B. thetaiotoamicron* continuously forages on these substrates in vivo.

To better understand the specificity of PUL expression in response to different glycans, we also evaluated the plant glycan-inducible responses of 12 previously identified PULs, known to orchestrate the degradation of host-derived glycans [8]. Despite the fact that some sugars like galactose and fucose are common to plant and host glycans, PULs specific for host glycans were not expressed in the presence of plant pectins or vice versa (compare columns A and B in the top and bottom sections of Figure 1), suggesting that individual PULs are activated in response to more complicated and unique oligosaccharide signals. Experiments that probe the specificity of HTCS regulators for oligosaccharide signals described later in this report support this view.

A notable feature of the pectin-specific PULs summarized in Figure 1 is activation of some PULs (e.g., *BT4145-4183*) by multiple substrates. A likely explanation for this phenomenon is that the preparations used for the growth assays were contaminated with trace amounts of other pectic glycans due to the inherent complexity and covalent connections originally present in these molecules prior to purification. This conclusion is supported by compositional analysis data available from the supplier: each preparation contained 5%–18% “contaminating” sugars that are not expected to be part of the purified pectic glycan but are present in other chains attached to a common backbone polysaccharide. Also, consistent with this notion, each PUL typically showed a much stronger transcriptional response on one substrate compared to the others. For example, *BT4108-23* showed highest induction on homogalacturonan and lower overall responses to three other pectins—pectic galactan and rhamnogalacturonans I and II. Compositional analysis of the former two substrates indicated the presence of homogalacturonan contamination, whereas homogalacturonan is a backbone component of the rhamnogalacturonan II structure [24]. Thus, it is likely that *BT4108-23* primarily targets homogalacturonan and that this substrate is responsible for its activation. This notion is compatible with the observed carbohydrate active enzyme (CAZyme) content of this PUL, which encodes seven enzymes that are members of families known to target homogalacturonan. Biochemical analysis of one of the CAZymes from the PUL (BT4116; a predicted family 1 polysaccharide lyase) supports this view as the enzyme was able to cleave homogalacturonan in an endo-like fashion but displayed no activity against rhamnogalacturonan I (Figure S2 and Table S2).

A feature shared by 8 of the 10 *B. thetaiotaomicron* PULs activated by pectin is their association with a HTCS regulator. To further dissect the involvement of specific PULs in pectin degradation, we disrupted the HTCS genes associated with pectin-activated PULs as well as several others associated with additional PULs (Table 2; see Figure S1 for a schematic of HTCS mutant locations and Table S3 for a list of all HTCS mutant strains tested). Six of the mutants with disruptions in HTCS genes linked to pectin-induced PULs resulted in a growth defect on different pectins (Table 2 and Figure S4). In contrast to the previously observed complete loss of growth after disruption of three *B. thetaiotaomicron* HTCS regulators associated with GAG- or fructan-utilizing PULs [8],[12], none of the disrupted HTCS genes linked to pectin-induced PULs resulted in such a drastic phenotype on any of the pectins tested. Thus, although no individual PUL is absolutely essential for full degradation of a particular pectin preparation tested, each of the six individual PULs identified appear to be optimized for degradation of a specific substrate. This observation is supported by data presented below that show that individual PULs are specifically activated by defined oligosaccharides derived from the different pectins.

**Table 2:** Defects associated with loss of pectin sensing HTCS regulators in *B. thetaiotaomicron*.

PUL	Primary Substrate	HTCS Mutant Growth Defect on Primary Substrate (Δ*HTCS*)^a^^,^ b
*BT3674-87*	arabinogalactan	149.9±4.4% of wild-type (Δ*BT3678*)
*BT0262-90*	arabinogalactan	78.9±5.5% of wild-type growth (Δ*BT0267*)
*BT0348-69*	arabinan	48.3±4.9% of wild-type growth (Δ*BT0366*)
*BT3043-48*	arabinan	none (Δ*BT3049*)
*BT4145-83*	rhamnogalacturonan I	58.5±2.0% of wild-type (Δ*BT4178*)^c^
*BT0977-30*	rhamnogalacturonan II	78.3±4.2% of wild-type (Δ*BT0981*)
*BT4667-72*	pectic galactan	21.6±1.0% of wild-type (Δ*BT4673*)
*BT4108-23*	homogalacturonan	11.6±0.9% of wild-type growth (Δ*BT4111*)^d^

a None of the HTCS mutants indicated exhibited defects on substrates other than those noted.

b Values provided are average percent of wild-type growth density (*n* = ≥5 replicates per mutant). *p* values for all mutant growths were <0.00001 compared to wild-type replicates.

c Disruption of an additional HTCS gene *BT4182* in this locus did not result in any growth defects on the pectins tested.

d Disruption of an additional HTCS gene *BT4124* in this locus did not result in any growth defects on the pectins tested.

### Several Unique *B. ovatus* PULs Target Hemicellulose

Our phenotypic analysis indicated that *B. ovatus* has evolved a capacity to target a series of hemicellulosic polysaccharides that *B. thetaiotaomicron* cannot access. To determine which *B. ovatus* genes are involved in hemicellulose metabolism, we performed transcriptional profiling experiments on *B. ovatus* cells grown on each of the different hemicelluloses (Table 1). This species was also profiled on the pectin homogalacturonan so that we could compare its responses to that of *B. thetaiotaomicron* on the same substrate. Growth of *B. ovatus* on individual hemicellulose preparations or homogalacturonan resulted in altered expression of 259 total genes using the same ≥10-fold cutoff threshold used for *B. thetaiotaomicron*: 229 of these genes were upregulated, while 30 were downregulated. As with *B. thetaiotaomicron*, most of the *B. ovatus* genes were uniquely expressed in response to just one or a few of the carbohydrates (Table S4).

**Figure 2:**
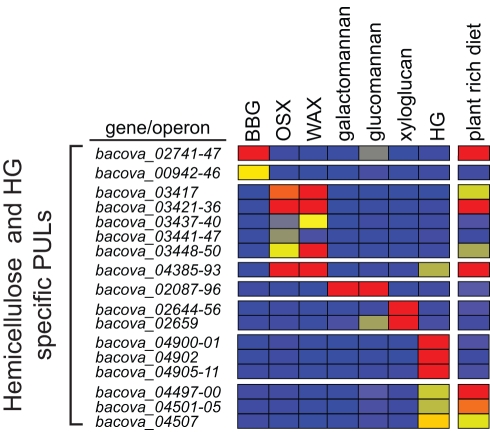
*B. ovatus* PULs expressed in response to plant cell wall glycans. A heatmap showing PUL operon induction by various hemicelluloses and homogalacturonan. Data analysis is identical to that described for *B. thetaiotaomicron* in Figure 1, including comparison to a MM-G reference and use of a ≥10-fold cutoff. The rightmost column in the heatmap shows the responses of PULs in the ceca of mice fed the same plant-rich diet used for *B. thetaiotoamicron*. Heatmap values are calibrated according to the bar shown in Figure 1. A Venn diagram illustration of total gene changes in these conditions is provided in Figure S6. BBG, barley β-glucan; OSX, oat spelt xylan; WAX, wheat arabinoxylan; HG, homogalacturonan.

We next identified putative PULs in the *B. ovatus* genome using the same criteria as those used previously for *B. thetaiotaomicron* [8]. Minimally, a PUL had to contain at least a pair of genes encoding homologs of the *B. thetaiotaomicron* SusC/D proteins. This effort yielded 112 candidate *B. ovatus* PULs encompassing 1,129 ORFs (see Table S5 for a list of genes and annotations by PUL). As observed in *B. thetaiotaomicron*, most annotated PULs also contained one or more genes encoding predicted CAZymes and/or an environmental sensor/transcriptional regulator. In total, 140 (61.1%) of the genes that were upregulated in response to growth on one or more glycans were located in PULs. Six different PULs were activated by hemicellulosic polysaccharides: two by xylans, one by galacto-/glucomannan, one by xyloglucan, and two by barley β-glucan (Figure 2).

The predicted CAZyme content of the six PULs activated by hemicelluloses (Table S2 and Figure S6) is consistent with their capacity to orchestrate degradation of the inducing polysaccharides. For example, the xylan-, xyloglucan-, and galacto-/glucomannan-regulated PULs encode GH10, GH9, and GH26 enzymes, respectively, families dominated by xylanases (GH10), endoglucanases (GH9), and β-mannanases (GH26). Indeed, a previous study has shown that two enzymes encoded within the smaller of the two xylan activated PULs (*BACOVA_04387* and *BACOVA_04386*) display endo-xylanase and β-xylosidase activities, respectively [25]. In addition, a PUL that is homologous to the smaller *B. ovatus* xylan PUL was recently identified in the rumen Bacteroidete *Prevotella bryantii* using a transcriptomic approach and wheat arabinoxylan as a test substrate [11]. Finally, several of the enzymes from the xylan-activated PULs contain carbohydrate binding modules from families known to display xylan binding functionality (CBM6, 22, and 35), adding further support for a role of products of these loci in xylan utilization. Interestingly, the large xylan PUL also contains a number of genes encoding enzymes from CAZyme families not previously implicated in xylan deconstruction (e.g., GH31, GH95, GH97, GH98), suggesting that these sequences may exhibit novel specificities or target linkages so far not identified in various xylans (Table S2).

Together, these findings support our conclusion that both *B. ovatus* and *B. thetaiotaomicron* rely on similar PUL-based strategies to degrade plant cell wall glycans. They also highlight how broadly Bacteroidetes Sus-like systems have evolved and further define the experimentally demonstrated range of substrates they target to include all major classes of hemicelluloses.

We subsequently measured *B. ovatus* PUL gene expression in the distal gut (cecum) of mono-associated gnotobiotic mice fed the same plant glycan-rich diet used to examine in vivo expression of *B. thetaiotaomicron*. A total of 353 *B. ovatus* genes exhibited altered expression in vivo relative to in vitro growth in MM-G: 126 genes also exhibited altered expression in the presence of one or more of the plant cell wall glycans tested and 50% of all in vivo responsive *B. ovatus* genes were located within putative PULs (Figure S5 and Table S5). Three *B. ovatus* PULs that were activated by the hemicelluloses xylan and β-glucan were expressed in vivo, suggesting that these substrates were present in the plant-rich diet fed to mice and could be sensed by this species in the absence of other members of the human gut microbiota (Figure 2). One of two *B. ovatus* PULs that responded to homogalacturonan in vitro was also expressed in vivo.

### Evolution of PULs in the *B. thetaiotaomicron* and *B. ovatus* Genomes

**Figure 3:**
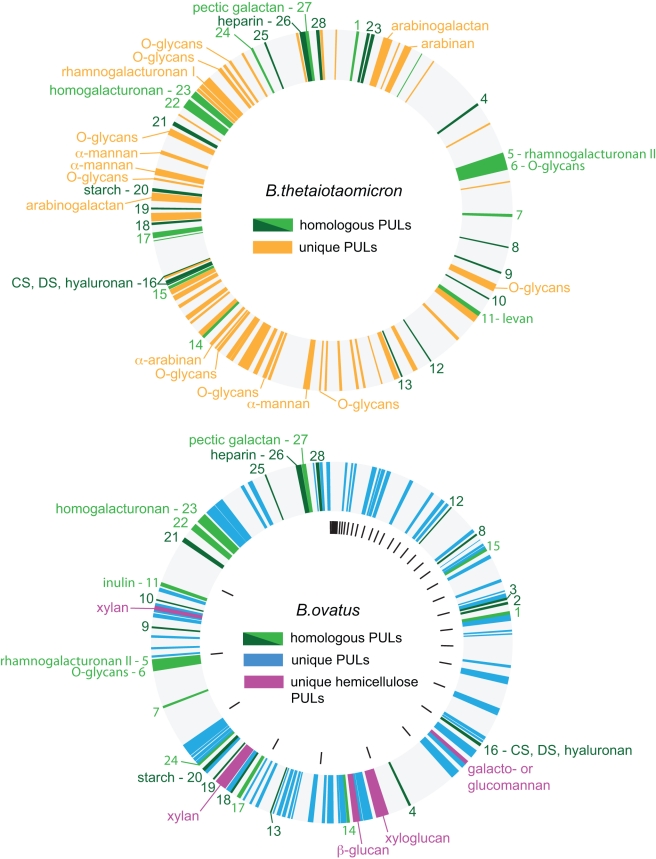
Comparisons of PULs in the sequenced type strain genomes of *B. thetaiotaomicron* and *B. ovatus*. The arrangement of PULs in the genome of each species is illustrated as a circular map with genes color coded as follows: “homologous PULs” (dark green); “probably homologous PULs” (light green); *B. thetaiotaomicron*-specific PULs (gold); *B. ovatus* specific PULs (light blue), *B. ovatus*-specific hemicellulose PULs (pink); all other genes in each species (gray). Shared PULs are labeled 1 through 28 based on their order in the *B. thetaiotaomicron* genome (clockwise from the top). Contigs in the deep-draft *B. ovatus* genome assembly are arranged in order of increasing size (clockwise from the top). Gaps in the genome assembly are illustrated as black tick marks around the interior of the *B. ovatus* genome. Empirically measured in vitro substrate specificities for some PULs are labeled around each genome schematic and correspond to PULs that were induced ≥10-fold in response to the indicated glycan class in this or previous studies [8],[12]. Homologous *B. ovatus* PULs that correspond to a *B. thetaiotaomicron* locus with known substrate response are also labeled with that substrate name. CS, chondroitin sulfate; DS, dermatan sulfate.

Given the partially overlapping sets of carbohydrate degradation traits exhibited by *B. thetaiotaomicron* and *B. ovatus*, we wanted to examine the degree to which individual orthologous PULs were maintained between these two *Bacteroides*. We reasoned that PULs that are unique to either genome would provide evidence of independent acquisition or retention of traits that are not shared between the species. Because the genome sequence of *B. thetaiotaomicron* VPI-5482 has been assembled into a single circular chromosome and contains fewer PULs than *B. ovatus*, we performed individual searches, using each of the 88 individual *B. thetaiotaomicron* PULs as queries, to probe for similar loci in the deep draft assembly of the *B. ovatus* genome. Our method (described in detail in Materials and Methods) was based on first comparing the core SusC/D amino acid sequences from each *B. thetaiotaomicron* PUL to the closest set of homologs in *B. ovatus* and then to score potentially homologous PULs for both gene homology and synteny within the PUL and in flanking genomic regions. Using this approach, each of the 198 PULs in the two *Bacteroides* species was scored as “homologous,” “probably homologous,” or “unique” to a respective species (Table S6). Only 28 PULs met our criteria for being homologous between *B. thetaiotaomicron* and *B. ovatus* (i.e., included in the homologous or probably homologous groups), suggesting that differential acquisition or retention of novel PULs is a mechanism underlying the phenotypic differences between these species (Figure 3). Among the group of PULs shared by both species were loci corresponding to each of the glycan metabolic traits that were strongly exhibited by both species: these include PULs for targeting starch, fructans, glycosaminoglycans, and all pectins except arabinan and arabinogalactan (PULs with dark or light green labels in Figure 3).

Evidence of the divergent evolution of these two species becomes apparent through visualization of the non-homologous PULs in their respective genomes. *B. thetaiotaomicron* contains at least eight unique PULs associated with targeting host mucin *O*-glycans (see PULs with gold labels in Figure 3 that are noted as “*O*-glycans”; this designation only includes PULs that have been confirmed to respond to purified neutral mucin *O*-glycans in vitro [8]). In addition, three previously validated PULs for degrading α-mannan are also unique to *B. thetaiotaomicron*. Conversely, *B. ovatus* contains five unique PULs that underlie its ability to target plant cell wall hemicelluloses. An additional PUL (*BACOVA_0942-46*) that responded to β-glucan weaker than a second PUL (*BACOVA_02741-47*) was scored as homologous.

The PULs that encode each species' unique phenotypes are scattered throughout each genome, suggesting that they arose through individual genetic events. This latter observation provides evidence for the idea that these two species are adapting to different carbohydrate niches and that these adaptations could serve to exclude access to others. For example, if *B. ovatus* had obtained its hemicellulose utilization PULs by lateral gene transfer or from a common ancestor, it might be expected that *B. thetaiotaomicron* would have been exposed to this same pool of traits during its own evolution. However, the sequenced strain of *B. thetaiotaomicron* analyzed here has not acquired any of these individual loci, nor do any of several dozen different *B. thetaiotaomicron* strains tested exhibit growth on hemicelluloses [7] (N. Pudlo and E. C. Martens, unpublished). Thus, *B. ovatus* appears to have evolved a predilection for hemicellulose degradation that has resulted in its retention of PULs that target a family of glycans occupying similar positions in the plant cell wall. A similar picture emerges for *B. thetaiotaomicron*, which has evolved a predilection for host mucin *O*-glycans and possibly *N*-glycans as evidenced by its robust ability to degrade the linkages in α-mannan.

### *Bacteroides* HTCS Recognize Complex Oligosaccharide Signals

The data presented above indicate that each *Bacteroides* PUL recognizes a specific molecular cue that is a component of its target polysaccharide. The most common class of regulator associated with PULs that target plant cell wall glycans are HTCS, inner membrane spanning proteins with predicted periplasmic sensory domains [12],[17]. A potential mechanism of signal perception by these regulators is direct binding of an oligosaccharide degradation product to the periplasmic domain of the HTCS. As noted above, this mechanism of signal perception has already been validated for one HTCS (BT1754) from *B. thetaiotaomicron*'s fructan utilization PUL [12]. However, unlike BT1754, which recognizes monomeric fructose and contains a ∼300 aa sensory domain that adopts the periplasmic binding protein fold, the majority of *Bacteroidetes* HTCS contain a much larger putative periplasmic sensor domain of ∼700–900 aa [26]. Sequence analysis revealed that these large sensor domains contain multiple short motifs (Reg_prop, Pfam 07494) indicative of an overall β-propeller fold, followed by a domain of ∼120 aa termed YYY (Pfam 07495) [27],[28]. In addition to the predicted N-terminal periplasmic domain, most HTCS polypeptides possess all of the cytoplasmic domains present in a classical two component system phosphorelay [26], but in a single polypeptide, including a phosphoacceptor and dimerization domain, histidine kinase, receiver domain, and a DNA binding domain of the HTH_AraC family (Figure S7).

**Figure 4:**
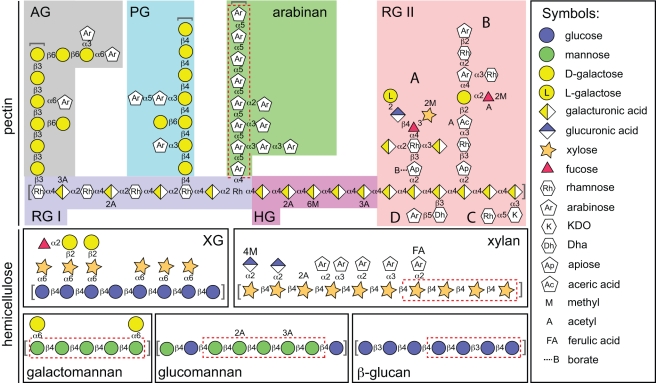
Schematic of the structures of plant cell wall pectins and hemicelluloses. Various representative glycan structures presented to *B. thetaiotaomicron* and *B. ovatus* in this study. The dashed red lines show the identity of the oligosaccharide signal molecules that preferentially activate PUL expression. The key to the abbreviated polysaccharides is provided in the legends to Figures 1 and 2. Brackets at the end(s) of glycan chains indicate that the actual glycan structure can be longer than that which is illustrated. Definitions of symbols used are provided in the key at the right. Note that the structures drawn are representative of predominant linkages and monosaccharides contained in each of the glycans. However, the illustrated glycans do not represent exact structures and many permutations are possible that vary in terms of chain length, methyl or acetyl substitution, or branch configurations, depending upon the species or tissue of origin, and/or the state of cellular differentiation.

To explore the mechanism of signal perception and identity in the HTCS proteins and further dissect their specificity, we expressed and purified the predicted periplasmic domains of several *B. thetaiotaomicron* and *B. ovatus* HTCS identified above as being involved in plant glycan utilization and assessed their ability to bind carbohydrates using isothermal titration calorimetry (ITC). The ITC data reveal that the periplasmic domains of four HTCS bind specifically to oligosaccharides that uniquely define the parent polysaccharide that the cognate PUL is optimized to degrade. Binding data are summarized in Table 3 and Figure S8, molecular illustrations of oligosaccharide signaling molecules that optimally bind to each HTCS sensor domain are shown in Figure 4, and detailed findings for each individual glycan are provided in Text S1. Notably, the binding of oligosaccharides to the HTCS is highly specific with each sensor domain only displaying affinity for glycan fragments derived from a single type of polysaccharide (e.g., the arabinan sensor BT0366 binds only arabino-oligosaccharides and not other oligosaccharides tested) (see footnote to Table 3).

**Table 3:** ITC analysis of HTCS periplasmic domains binding to oligo- and polysaccharides.

HTCS	Saccharide	Ka	ΔG	ΔH	TΔS	*N*
		(M^−1^)	(kcal.mol^−1^)	(kcal.mol^−1^)	(kcal.mol^−1^)	
**BT0366 (arabinan)** a	Arabinobiose-Arabinotetraose	NB^b^	—	—	—	—
	Arabinopentaose	NB	—	—	—	—
	Arabinohexaose	3.9E3±0.9^c^	−4.9±0.1	+7.6±0.7	+12.5±0.6	1.2±0.2
		1.5E3±0.1	−4.3±0.1	−6.5±2.0	−2.2±2.1	4.6±0.7
	Arabinoheptaose	3.5E4±0.6	−6.2±0.1	+0.6±0.3	+6.8±0.2	1.3±0.1
		3.2E3±0.2	−4.8±0.0	−12.5±2.1	−7.7±2.1	1.6±0.2
	Arabinooctaose^d^	4.8E4±0.1	−6.4±0.0	+1.6±0.7	+8.0±0.7	0.7±0.1
		5.0E3±0.6	−5.0±0.1	−17.5±2.1	−12.5±2.0	1.3±0.1
	Arabinan (linear)^e^	1.0E5±0.0	−6.8±0.0	−26.8±0.0	−20.0±0.1	—
**BACOVA_04394 (xylan)**	Xylobiose	TLTF^f^	—	—	—	—
	Xylotriose	1.1E3±0.1	−4.1±0.1	−12.9±0.1	−8.8±0.2	1.0±0.0
	Xylotetraose^d^	6.8E5±1.2	−7.95±0.1	−10.5±1.2	−2.55±1.2	1.3±0.1
	Xylopentaose	8.8E4±2.9	−6.7±0.2	−1.2±0.1	+5.5±0.3	1.1±0.2
	Xylohexaose	TLTF	—	—	—	—
	Xylan (Oat)	NB	—	—	—	—
**BACOVA_02740 (β-glucan)**	Glucotriose A (β3,4)	NB	—	—	—	—
	Glucotriose B (β4,3)	3.4E3±1.9	−4.8±0.3	−9.65±1.9	−4.85±2.1	1.1±0.1
	Glucotetraose B (β4,4,3)	2.5E3±1.3	−4.6±0.3	−8.6±1.4	−4.0±1.1	0.9±0.2
	Glucotetraose C (β4,3,4)^d^	3.2E4±0.7	−6.1±0.1	−14.0±1.0	−8.1±1.1	1.2±0.1
	Glucopentaose A (β3,4,4,4)	TLTF	—	—	—	—
	Cellobiose	TLTF	—	—	—	—
	β1,4 Cello-oligosaccharides	NB	—	—	—	—
	(3–6 sugars)					
	β1,3 Laminari-oligosaccharides (2–6 sugars)	NB	—	—	—	—
	β-glucan (Barley)	NB	—	—	—	—
**BACOVA_02097 (galacto-/glucomannan)**	Mannobiose+Mannotriose	NB	—	—	—	—
	Mannotetraose	2.0E4±0.6	−5.9±0.2	+2.3±1.5	+8.2±1.3	1.0±0.0
		2.7E3±0.3	−4.7±0.1	−5.4±0.3	−0.7±0.2	4.8±0.5
	Mannopentaose	1.95E5±0.25	−7.2±0.1	+0.4±0.1	+7.6±0.2	0.2±0.0
		4.65E3±0.25	−5.0±0.0	−34.7±1.5	−29.7±1.5	1.0±0.1
	Mannohexaose^d^	1.0E6±1.2	−8.2±0.1	+1.4±0.2	+9.6±0.0	0.05±0.01
		3.6E4±1.4	−6.2±0.0	−26.0±3.0	−19.8±3.0	1.2±0.1
	Galactomannan (Carob)^e^	6.0E5±2.4	−7.9±0.2	−8.6±0.1	−2.0±0.1	—
		6.4E4±0.4	−6.55±0.1	−23.2±1.6	−16.45±1.8	—
	Di-galactosyl mannopentaose^g^	NB	—	—	—	—

a Locus tag of HTCS with polysaccharide that activates associated PUL in parentheses.

b NB, no binding.

c Data are averages and SDs of at least triplicate titrations at 25°C, 50 mM Hepes buffer, pH 8.0. Binding of oligosaccharides to BT0366 and both oligo- and polysaccharides to BACOVA_02097 were fit to a two-site binding model. Values reported are for site 1 then site 2.

d Representative titration curves are provided in Figure S7.

e Fit as ligand in cell.

f TLTF, too low to fit (i.e., binding event observed, but affinity too low to accurately fit the data. Ka<∼5.0×10^2^ M^−1^).

g Megazyme cat. no. OGGM5.

Each HTCS sensor domain was also evaluated for binding to the other oligosaccharides tested from non-inducing glycan sources. In all cases, no binding was detected.

Unlike the previously described fructose-sensing HTCS that binds a simpler monosaccharide signal [12], all of the HTCS sensors described here interacted directly with oligosaccharides, consistent with the idea that the specific recognition of most polysaccharides requires information contained in both the sugar content and glycosidic linkages. By sensing oligosaccharide cues that uniquely define the parent polysaccharide, *B. thetaiotaomicron* and *B. ovatus* (and by extension other Bacteroidetes that contain PUL-associated HTCS) are able to differentiate between multiple complex glycans that contain the same sugars, and to respond efficiently by activating only the appropriate PUL for their degradation. Interestingly, the preferred ligands for the HTCS were relatively large glycan fragments ranging from tetra- to octa-saccharides and therefore represent early products of the depolymerization process that are likely transported into the periplasm via their specific PUL-encoded SusC-like outer membrane transporter, as has been suggested for maltooligosaccharides for the starch-utilization system [29]. Together, these data suggest that Bacteroides respond rapidly and specifically to the presence of polysaccharides in their environment. This requirement for a rapid and highly defined response may explain the localization of the signal input and output domains of HTCS in a single polypeptide, as this physical constraint will both maximize the speed of activation and minimize cross-talk among these systems. It is also notable that all four HTCS sensors interacted directly with linear oligosaccharides, suggesting that the presence of branches, which can vary between related glycans from different sources, is not a required signaling component for this subset of sensors. In one case (mannopentaose versus di-galactosyl-mannopentaose), the presence of galactose branching interfered completely with detectable binding, suggesting that removal of these branches is a prerequisite for sensing of galactomannans.

**Figure 5:**
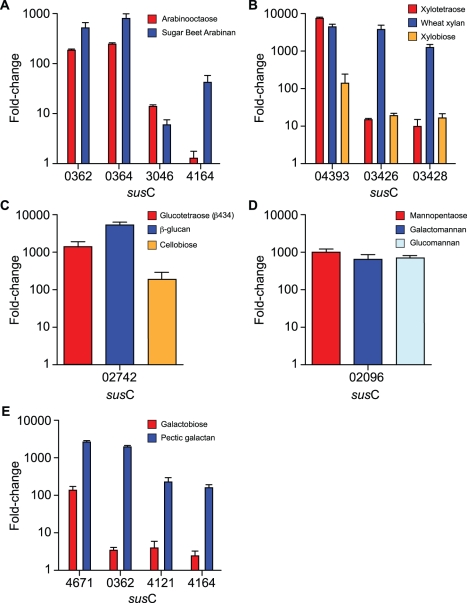
Plant glycan PULs are specifically activated by signature oligosaccharides that define their target polysaccharide. Cells were grown on MM supplemented with polysaccharide, oligosaccharide, or glucose as the sole carbon source, and levels of different *susC* transcripts in each condition were quantified by qPCR. The *susC* genes chosen as markers for expression of their cognate PUL were identified from the GeneChip data. Panels (A) and (E) are *B. thetaiotaomicron*. Panels (B–D) are *B. ovatus*. The *y*-axis shows the fold-change relative to a MM-G reference; *x*-axis labels indicate the locus tag of the *susC* gene probed in each condition. Data are averages and standard deviations of three biological replicates.

To confirm that the oligosaccharides we identified by ITC were able to specifically activate PUL gene expression in bacterial cells, we measured the relative amount of the *sus*C-like gene transcript (a proxy for expression of the whole operon) of each PUL when the bacteria were grown on the cognate HTCS ligand. The data reveal that the oligosaccharide that binds preferentially to the HTCS specifically upregulates the *sus*C-like gene, and by inference the whole PUL, associated with that HTCS (Figure 5). For example, exposure of *B. thetaiotaomicron* to arabino-octaose (the preferred ligand for BT0366 HTCS) results in a 10–100-fold greater induction of the *sus*C homolog present in the BT0366-associated PUL (*BT0348-69*) compared to its effects on the other PULs that are activated by arabinan, but whose associated HTCS display no binding to arabino-oligosaccharides (250–400-fold for *susC*-like genes *BT0362* and *BT0364* compared to 15- and 2-fold for the *sus*C-like genes, *BT3046* and *BT4164*). Similarly, growth of *B. ovatus* on xylotetraose, the preferred ligand for BACOVA_04394, upregulates the *susC*-like gene (*BACOVA_04393*) associated with this PUL, but not two *susC*-like genes from the larger xylan PUL (*BACOVA_03426* and *_03428*). These findings support the HTCS binding data and demonstrate that activation of each PUL is by a defined oligosaccharide cue that is specifically recognized by the associated HTCS (i.e., there is little or no cross-reactivity between the PULs). Additional details of the transcriptional response of *B. thetaiotaomicron* to pectic oligosaccharides are provided in Text S1.

### Prospectus

The distal gut of humans is constantly inundated with a dynamic array of carbohydrates. These substrates feed the dense consortium of microbes that compete in this habitat. The gut also presents biochemical gradients that result from the differential rates of digestion of dietary resources and from the presence of a mucus layer overlying the epithelium. Given the gut's intrinsic biochemical heterogeneity, it is perhaps not surprising that different microbial lineages would evolve to fill distinct glycan niches. The data presented here support the notion that two closely related human gut symbionts have taken divergent paths that have left each species with a partially unique repertoire of metabolic traits. Although *B. thetaiotaomicron* and *B. ovatus* are both common in the normal adult human gut microbiota, at least in Western societies [5], they represent only two of at least 45 different species of Bacteroidetes that have been cultured to date from human specimens. Complete or deep-draft genomic sequences will soon be available for many more members of these species as culturing efforts [30] and sequencing technology progress rapidly. The availability of additional cultured Bacteroidetes, together with the ability to dissect their carbohydrate active phenotypes using the approaches described here, present an opportunity to reveal basic biological parameters that have catalyzed the niche-specific adaptation of gut bacterial lineages throughout human history. At the same time, identifying the molecular machinery for acquisition, import, and catabolism of specific polysaccharides will help inform efforts to engineer carbohydrate active phenotypes in microbes (via PUL “transplants”) in order to fulfill important industrial needs, as well as to manipulate human gut microbiome function in ways that restore health or enhance nutrition.

## Materials and Methods

### Ethics Statement

All experiments involving mice used protocols approved by the Washington University Animal Studies Committee in accordance with guidelines set forth by the American Veterinary Medical Association. Trained veterinarians from the Washington University Division of Comparative Medicine supervised all experiments. The laboratory animal program at Washington University is accredited by the Association for Assessment and Accreditation of Laboratory Animal Care International (AAALAC).

### Bacterial Growth and Phenotypic Profiling on a Custom Carbohydrate Array

*B. thetaiotaomicron* ATCC 29148 (VPI-5482) and *B. ovatus* ATCC 8483 were routinely grown in tryptone-yeast extract-glucose (TYG) medium [31] or on brain-heart infusion (BHI; Beckton Dickinson) agar plus 10% horse blood (Colorado Serum Co.). Antibiotics were added as appropriate: erythromycin (25 µg/ml) and gentamicin (200 µg/ml). Minimal medium (MM) was formulated as previously described [8]. *B. thetaiotaomicron* HTCS mutant strains were constructed by suicide plasmid insertion (denoted as “Ω” mutants) [32].

The ability of *B. thetaiotaomicron* and *B. ovatus* to grow on pure carbohydrates was measured using a custom carbohydrate array constructed in a 96-well format. Each well of a flat bottom 96-well plate (Costar) was loaded with 100 µl of each sterilized carbohydrate stock (Table S7) at 2× concentration. Each substrate was represented twice on each assay plate in two non-adjacent wells. Two carbohydrate-free water wells were included as negative controls. Cultures for assay inoculations were grown for ∼24 h at 37°C under an atmosphere of 10% H_2_, 5% CO_2_, and 85% N_2_ in MM-G, and a 1 ml aliquot centrifuged to pellet bacteria, which were then gently resuspended in 2× MM-no carbohydrate (MM-NC) and used to inoculate 50 ml of 2× MM-NC at a ratio of 1∶50. Each carbohydrate array was loaded with 100 µl of the inoculated 2× medium to produce 96 individual 200 µl cultures. Assay plates were sealed in an anaerobic chamber (Coy manufacturing, Grass Lake, MI) under the atmosphere noted above with an optically clear gas-permeable polyurethane membrane (Diversified Biotech, Boston, MA). Plates were then loaded into a Biostack automated plate handling device coupled to a Powerwave HT absorbance reader (both devices from Biotek Instruments, Winooski, VT). Absorbance at 600 nm (*A*_600_) was measured for each well at 10–15 min intervals. *B. thetaiotaomiocron* and *B. ovatus* were each tested in three separate carbohydrate arrays (*n* = 6 replicate cultures). Data were processed using Gen5 software (Biotek) and Microsoft Excel.

Several glycans yielded complicated polyphasic growth profiles rather than a single exponential growth phase. Thus, we quantified growth in each assay by first identifying a minimum time point (*A*_min_) at which *A*_600_ had increased by 10% over a baseline reading taken during the first 500 min of incubation. Next, we identified the time point at which *A*_600_ reached its maximum (*A*_max_) immediately after exponential growth. Two growth parameters were generated for each well: “rate” [(*A*_max_−*A*_min_)/(T_max_−T_min_)], and “density” (*A*_max_−*A*_min_), where T_max_ and T_min_ are the corresponding time values for each absorbance. Cultures that failed to increase density by at least 0.1 (*A*_600_) were scored as no growth.

Cultures for transcriptional profiling were grown in borosilicate test tubes containing 5 ml of the same MM formulations described above, except that rhamnogalacturonan II was used at 15 mg/ml for transcriptional profiling experiments. All cultures were harvested during mid- to late-exponential phase; absorbance values (at 600 nm) of each harvested culture are summarized in Table S8.

### Purification of Select Glycan Substrates for Bacterial Growth

Water soluble oat spelt xylan (OSX) was prepared by solubilizing oat spelt xylan (Fluka) in 1 M NaOH followed by centrifugation (8,750× g for 30 min) to remove insoluble material. The soluble supernatant was then adjusted to pH 7.0 with HCl, centrifuged again to remove insoluble glycans that precipitated at neutral pH, dialyzed exhaustively against ddH_2_O and finally dried by lyophilization. Rhamnogalacturonan II (a kind gift from Malcolm O'Neil at the University of Georgia Complex Carbohydrate Research Center) was purified from red wine as described previously [33].

### Whole-Genome Transcriptional Profiling

Transcriptional profiling was performed using custom Affymetrix GeneChips containing probesets representing >98% of 4,779 predicted *B. thetaiotaomicron* genes [20], and all of the 5,536 predicted *B. ovatus* genes. GeneChip targets were prepared from whole bacterial RNA and hybridized to the microarrays according to standard Affymetrix protocols (www.affymetrix.com). Data were normalized using Microarray Suite 5 or Expression Console software (Affymetrix) and processed using GeneSpringGX 7.3.1 software (Agilent) according to a previously described workflow [8]. Further details concerning bacterial growth conditions and experimental parameters are provided in Table S9, along with individual GEO accession numbers and file names for each dataset.

### Colonization of Germfree Mice with *B. ovatus*

All mice were from the NMRI-KI inbred line and were reared in gnotobiotic isolators as previously described [21]. Six-week-old male germfree animals were used for *B. ovatus* colonization. Each mouse was gavaged with 100 µl of a fresh overnight culture containing ∼1×10^8^ cfu/ml. *B. ovatus* colonization levels in the cecum were between 5×10^10^ and 5×10^11^ cfu/ml for all animals. Animals were sacrificed 14 d after colonization and their cecal contents harvested for RNA extraction.

### Comparison of PULs between the *B. thetaiotaomicron* and *B. ovatus* Genomes

To locate putative PULs, the publicly available *B. ovatus* ORF annotation was searched using an iterative BLAST strategy described for other Bacteroidetes species [34]. This process yielded 112 *B. ovatus* PULs that minimally contained homologs of *susC/susD*. To compare PUL gene content between species we used a list of reciprocal best BLASTP hits between the *B. thetaiotaomicron* and *B. ovatus* genomes (E-value cutoff ≤−10) and the “show ortholog neighborhood regions” in the Department of Energy Integrated Microbial Genome website (img.jgi.doe.gov) to guide analysis of PULs that were shared between these two species. Beginning with the first *susC* sequence in *B. thetaiotaomicron*, we searched for an orthologous neighborhood in *B. ovatus*. If this produced a hit, then we repeated the search with the adjacent *B. thetaiotaomicron susD* sequence to verify that the same locus was found in *B. ovatus*. We next compared the genomic regions surrounding each potentially orthologous PUL for conservation of gene content both within the PUL and in neighboring genomic regions. PULs were differentially scored for orthology between species based on the following criteria: (i) loci that had identical numbers of homologous PUL genes in the same orientation between species, and also contained at least three syntenic homologous genes in the region *flanking* the PUL, were scored as “orthologous PULs”; (ii) loci that exhibited similar numbers of homologous PUL genes but in different orientation between species and still contained at least three syntenic homologous genes in the region flanking the PUL were scored as “probably orthologous PULs”; (iii) PULs that exhibited different numbers of genes with little or no apparent homology, poor conservation of functional predictions (e.g., carbohydrate active enzymes), and were not located at syntenic genomic regions were scored as “non-orthologous PULs.”

### Cloning, Expression, and Purification of Recombinant Proteins

DNA encoding the HTCS periplasmic domains were amplified from the appropriate species' genomic DNA using the primers stated in Table S9 and cloned into pET21d or pET28b (Novagen). The location of signal peptides and internal transmembrane domains in the HTCS proteins were predicted using the web-based programs SignalP 3.0 (http://www.cbs.dtu.dk/services/SignalP/) and TMPred (http://www.ch.embnet.org/software/TMPRED_form.html), respectively. *E. coli* BL21 or Tuner (Novagen) cells were used to express recombinant proteins, which were purified in a single step using metal affinity chromatography, as described previously [35].

### Isothermal Titration Calorimetry

ITC was performed essentially as described previously [34], using a Microcal VP-ITC. Proteins (50–200 µM, in cell) were dialyzed into 20 mM HEPES, pH 8.0, and ligands (0.5–20 mM oligosaccharides, 5–20 mg/ml polysaccharides, in syringe) were dissolved in the dialysis buffer to minimize heats of dilution. Integrated binding heat effects minus heats of dilution were analyzed by non-linear regression using either a single or two-site binding model (Microcal Origin 7.0 software).

### Quantitative PCR (qPCR)

Additional quantification of transcript expression was performed by qPCR using a Roche Lightcycler 480 and primers listed in Table S9. Bacteria were cultured in 5 ml of MM containing 0.5% carbon source, as described above. Triplicate bacterial cultures were harvested at mid-log phase and placed in RNAprotect (Qiagen) prior to purification with RNeasy kit (Qiagen). cDNA was produced with QuantiTect Reverse Transcription kit (Qiagen). qPCR was performed in a 96-well plate with SYBRgreen 480 I Master (Roche). Data were normalized to 16S rRNA transcript levels.

All oligosaccharides and polysaccharides (low viscosity) used for ITC and qPCR studies were from Megazyme (Wicklow, Ireland), except for oat spelt xylan and cellobiose, which were from Fluka. The water-soluble fraction of oat spelt xylan was used and prepared as described above.

### CAZyme Analysis of *B. thetaiotaomicron* and *B. ovatus* ATCC 8483

Each protein model encoded by the genomes of the two *Bacteroides* studied here was subjected to a combination of BLAST [36] and HMMer [37] searches against, respectively, sequence libraries built with the individual modules of the proteins found in the CAZy database (www.cazy.org), and HMM models built with each of the families present in CAZy [14]. To avoid missing distant relatives, permissive thresholds were used (E-value<0.1), and all resulting candidate proteins were manually screened by comparison to the CAZy families (multiple alignments; presence of catalytic residues where known; presence of appended catalytic and non-catalytic modules, etc.).

## Supporting Information

Text S1 Additional Results and Discussion of the *B. thetaiotaomicron* and *B. ovatus* plant cell wall degrading mechanisms.
(DOC)

Figure S1 Schematic of *B. thetaiotaomicron* PULs involved in plant glycan metabolism. (A) Eight HTCS-associated *B. thetaiotaomicron* PULs involved in plant pectin degradation. The HTCS genes (pink boxes) that were disrupted by plasmid insertions are labeled with an “Ω” symbol; black symbols indicate no phenotype was observed; red symbols indicate HTCS mutants that resulted in a growth-deficient phenotype. (B) Two *B. thetaiotaomicron* PULs expressed in response to rhamnogalacturonan II that lack an associated transcriptional regulator. Each gene is drawn to scale as a rectangle with its orientation indicated by the closed triangle. Dashed lines are used to connect linear segments and do not represent actual genomic distance. Genes that were not induced ≥10-fold in the indicated growth condition are shown as partially transparent (note that in all but two cases shown the HTCS regulator does not itself undergo any expression change). Genes encoding known or predicted functionalities are color coded: glycoside hydrolase (dark blue), polysaccharide lyase (light blue), carbohydrate esterase (light green), *susC*-like gene (purple), *susD*-like gene (orange), hybrid two-component system (pink), and other or unknown function (white). Genes encoding predicted enzymatic functions are also annotated according to their CAZy family number: glycoside hydrolase (GH), polysaccharide lyase (PL), and carbohydrate esterase (CE).
(EPS)

Figure S2 Activity and specificity of the family 1 polysaccharide lyase BT4116. Homogalacturonan (HG; non-methyl esterified) and rhamnogalacturonan I (RG) at 5 mg ml^−1^ were digested with 1 µM BT4116 in 20 mM Tris pH 8.0, 1 mM CaCl_2_ for 1 h at 37°C. TLC plates were analyzed in a solvent system of ethyl acetate, acetic acid, formic acid, and water (9∶3∶1∶4) and developed in orcinol. GalA standard (lane 1), HG+enzyme (lane 2), HG starting material (lane 3), RG+enzyme (lane 4), RG starting material (lane 5). Arrows show the positions of the main products of homogalacturonan digestion.
(EPS)

Figure S3 A Venn diagram of *B. thetaiotaomicron* genes that respond to plant and host glycans in vitro and in vivo. Comparison of genes from three different classes of growth conditions: in vitro growth on purified plant glycans; in vitro growth on purified host glycans; and in vivo growth in the ceca of mono-associated gnotobiotic mice consuming a diet rich in plant polysaccharides. All genes summarized exhibited ≥10-fold increased (“up”) or decreased (“down”) expression relative to growth on MM-glucose. These criteria are identical to those used in Figure 1 of the main text and in Table S1. Regulated genes from in vitro growth in the presence of the 7 plant glycans and 5 host glycans listed in Figure 1 of the main text were grouped together into the “in vitro plant glycan set” and “in vitro host glycans set,” respectively. Regions of overlap indicate inclusion of regulated genes from multiple lists.
(EPS)

Figure S4 Growth curves of *B. thetaiotaomicron* HTCS mutants on various pectins. In each panel, the growth profile of one or more HTCS mutants is compared directly to wild-type *B. thetaiotaomicron*. Six individual replicate cultures (200 µl each, in the same 96-well plate) were averaged to generate each curve. Error bars represent the standard deviation of each averaged value between the replicates. The average growth maximum for each mutant was compared to wild-type on the same substrate to quantify the growth defect in each mutant (see Table 2, main text).
(EPS)

Figure S5 A Venn diagram of *B. ovatus* genes that respond to plant glycans in vitro and in vivo. Comparison of genes from two different classes of growth conditions: in vitro growth on purified plant glycans; in vivo growth in the ceca of mice consuming a plant-rich diet. All genes summarized exhibited ≥10-fold increased (“up”) or decreased (“down”) expression relative to growth on MM-G; these are identical to the criteria used in Figure 2 of the main text and Table S4. Regulated genes from in vitro growth on the six-plant cell wall glycans listed in Figure 2 of the main text were grouped together into the in vitro plant glycans sets. Regions of overlap indicate inclusion of regulated genes from multiple lists.
(EPS)

Figure S6 Schematic of *B. ovatus* PULs involved in plant glycan metabolism. Seven *B. ovatus* PULs involved in hemicellulose or homogalacturonan degradation. Each gene is drawn to scale as a rectangle with its orientation indicated by the closed triangle. Dashed lines are used to connect linear segments and do not represent actual genomic distance. Genes that were not induced ≥10-fold in the indicated growth condition are shown as partially transparent (note that in all but two cases shown the HTCS regulator does not itself undergo any expression change). Genes encoding known or predicted functionalities are color coded: glycoside hydrolase (dark blue), polysaccharide lyase (light blue), carbohydrate esterase (light green), *susC*-like gene (purple), *susD*-like gene (orange), hybrid two-component system (pink), and other or unknown function (white). Genes encoding predicted enzymatic functions are also annotated according to their CAZy family number: glycoside hydrolase (GH), polysaccharide lyase (PL), and carbohydrate esterase (CE).
(EPS)

Figure S7 Domain structure of a typical Bacteroidetes hybrid two-component system (HTCS). The large (∼800 aa) periplasmic sensor domain is composed of a number of Reg_prop (Pfam 07494) repeat motifs predicted to form two 7-bladed β-propellers and a C-terminal YYY domain (Pfam 07495). Transmembrane domain is abbreviated TM. Conserved His (H) and Asp (D) residues in histidine kinase and receiver domains of the cytoplasmic phosphorelay are shown.
(EPS)

Figure S8 ITC data showing binding of the periplasmic domains of plant glycan PUL associated HTCS to their activating oligosaccharide signals. The upper parts of each panel show the raw binding heats and the lower parts the integrated heats fit to a single site (BACOVA_04394 and BACOVA_02740) or two-site (BT0366 and BACOVA_02097) binding model.
(EPS)

Table S1 *B. thetaiotaomicron* genes with altered expression in vitro during growth on plant and host glycans or in vivo in mono-associated gnotobiotic mice fed a plant glycan rich diet. Genes are separated according to the Venn diagram sectors delineated in Figure S4. Values shown are the mean fold differences compared to expression during growth in MM-G; only fields with values ≥10-fold are shown. Upregulated genes are shown in green; downregulated genes are shown in red. Empty cells indicate a fold-change value <10.
(PDF)

Table S2 Glycan degrading enzymes encoded in *B. thetaiotaomicron* and *B. ovatus* PULs. Glycan degrading enzymes are listed as annotated in the CAZy database [14]. Predicted target linkages are based on the known activities within each CAZy family and the linkages known to be present in the substrate presented for growth.
(PDF)

Table S3 Hybrid two-component system mutants analyzed in this study.
(PDF)

Table S4 *B. ovatus* genes with altered expression in vitro during growth on plant glycans or in mice fed a plant glycan rich diet. Mean fold differences in expression compared to growth on minimal medium plus glucose are noted. “Sector” designations for each sub-list refer to the Venn diagram in Figure S6.
(PDF)

Table S5 Putative PULs identified in the *B. ovatus* ATCC8384 genome. *B. ovatus* PULs that contain genes expressed in vivo in the ceca of mice fed a plant rich diet are indicated in the final column. Abbreviations and notes: extracytoplasmic function sigma factor, ECF; hybrid two-component system, HTCS; regulators with homology to other families of PUL-associated regulators are indicated (SusR, GntR, AraC); the presence of a potential regulator in a different family is indicated as “unknown.”
(PDF)

Table S6 Comparison of homologous PULs between *B. thetaiotaomicron* and *B. ovatus*. Dark green lines delineate “homologous PULs.” Light green lines delineate “probably homologous PULs.” Yellow lines delineate genes immediately adjacent to PULs that are syntenic best Blast hits. Gray lines delineate genes within homologous PULs that are not best Blast hits.
(PDF)

Table S7 Supplier information for carbohydrate growth array.
(PDF)

Table S8 Microarray datasets used in this study. All GeneChip data used in this study are available from the Gene Expression Omnibus (GEO) database (www.ncbi.nlm.nih.gov/projects/geo/) under the indicated accession number.
(PDF)

Table S9 Primers used in this study. Sequences recognized by restriction enzymes used for molecular cloning are underlined.
(PDF)

## Abbreviations

CAZy: Carbohydrate Active Enzymes
ECF-σ: extra-cytoplasmic function sigma
GAGs: glycosaminoglycans
HTCS: hybrid two-component system
ITC: isothermal titration calorimetry
MM: minimal medium
MM-NC: MM-no carbohydrate
OSX: oat spelt xylan
PULs: polysaccharide utilization loci
qPCR: Quantitative RT-PCR
Sus: starch-utilization system
TYG: tryptone-yeast extract-glucose

## References

[1] Flint H. J, Bayer E. A, Rincon M. T, Lamed R, White B. A (2008). Polysaccharide utilization by gut bacteria: potential for new insights from genomic analysis.. Nat Rev Microbiol.

[2] McNeil N. I (1984). The contribution of the large intestine to energy supplies in man.. Am J Clin Nutr.

[3] Eckburg P. B, Bik E. M, Bernstein C. N, Purdom E, Dethlefsen L (2005). Diversity of the human intestinal microbial flora.. Science.

[4] Ley R. E, Hamady M, Lozupone C, Turnbaugh P. J, Ramey R. R (2008). Evolution of mammals and their gut microbes.. Science.

[5] Qin J, Li R, Raes J, Arumugam M, Burgdorf K. S (2010). A human gut microbial gene catalogue established by metagenomic sequencing.. Nature.

[6] Muegge B. D, Kuczynski J, Knights D, Clemente J. C, Gonzalez A (2011). Diet drives convergence in gut microbiome functions across mammalian phylogeny and within humans.. Science.

[7] Salyers A. A, Vercellotti J. R, West S. E, Wilkins T. D (1977). Fermentation of mucin and plant polysaccharides by strains of Bacteroides from the human colon.. Appl Environ Microbiol.

[8] Martens E. C, Chiang H. C, Gordon J. I (2008). Mucosal glycan foraging enhances fitness and transmission of a saccharolytic human gut bacterial symbiont.. Cell Host Microbe.

[9] Martens E. C, Koropatkin N. M, Smith T. J, Gordon J. I (2009). Complex glycan catabolism by the human gut microbiota: the Bacteroidetes Sus-like paradigm.. J Biol Chem.

[10] Shipman J. A, Berleman J. E, Salyers A. A (2000). Characterization of four outer membrane proteins involved in binding starch to the cell surface of Bacteroides thetaiotaomicron.. J Bacteriol.

[11] Dodd D, Moon Y. H, Swaminathan K, Mackie R. I, Cann I. K (2010). Transcriptomic analyses of xylan degradation by Prevotella bryantii and insights into energy acquisition by xylanolytic bacteroidetes.. J Biol Chem.

[12] Sonnenburg E. D, Zheng H, Joglekar P, Higginbottom S. K, Firbank S. J (2010). Specificity of polysaccharide use in intestinal bacteroides species determines diet-induced microbiota alterations.. Cell.

[13] Koropatkin N. M, Martens E. C, Gordon J. I, Smith T. J (2008). Starch catabolism by a prominent human gut symbiont is directed by the recognition of amylose helices.. Structure.

[14] Cantarel B. L, Coutinho P. M, Rancurel C, Bernard T, Lombard V (2009). The Carbohydrate-Active EnZymes database (CAZy): an expert resource for glycogenomics.. Nucleic Acids Res.

[15] D'Elia J. N, Salyers A. A (1996). Effect of regulatory protein levels on utilization of starch by Bacteroides thetaiotaomicron.. J Bacteriol.

[16] Martens E. C, Roth R, Heuser J. E, Gordon J. I (2009). Coordinate regulation of glycan degradation and polysaccharide capsule biosynthesis by a prominent human gut symbiont.. Journal of Biological Chemistry.

[17] Sonnenburg E. D, Sonnenburg J. L, Manchester J. K, Hansen E. E, Chiang H. C (2006). A hybrid two-component system protein of a prominent human gut symbiont couples glycan sensing in vivo to carbohydrate metabolism.. Proc Natl Acad Sci U S A.

[18] Miyazaki K, Hirase T, Kojima Y, Flint H. J (2005). Medium- to large-sized xylo-oligosaccharides are responsible for xylanase induction in Prevotella bryantii B14.. Microbiology.

[19] Dodd D, Mackie R. I, Cann I. K (2010). Xylan degradation, a metabolic property shared by rumen and human colonic Bacteroidetes.. Mol Microbiol.

[20] Sonnenburg J. L, Xu J, Leip D. D, Chen C. H, Westover B. P (2005). Glycan foraging in vivo by an intestine-adapted bacterial symbiont.. Science.

[21] Bjursell M. K, Martens E. C, Gordon J. I (2006). Functional genomic and metabolic studies of the adaptations of a prominent adult human gut symbiont, Bacteroides thetaiotaomicron, to the suckling period.. J Biol Chem.

[22] Westover B. P, Buhler J. D, Sonnenburg J. L, Gordon J. I (2005). Operon prediction without a training set.. Bioinformatics.

[23] Cartmell A, McKee L. S, Pena M. J, Larsbrink J, Brumer H (2011). The structure and function of an arabinan-specific {alpha}-1,2-arabinofuranosidase identified from screening the activities of bacterial GH43 glycoside hydrolases.. J Biol Chem.

[24] Mohnen D (2008). Pectin structure and biosynthesis.. Curr Opin Plant Biol.

[25] Whitehead T. R, Hespell R. B (1990). The genes for three xylan-degrading activities from Bacteroides ovatus are clustered in a 3.8-kilobase region.. J Bacteriol.

[26] Xu J, Chiang H. C, Bjursell M. K, Gordon J. I (2004). Message from a human gut symbiont: sensitivity is a prerequisite for sharing.. Trends Microbiol.

[27] Mascher T, Helmann J. D, Unden G (2006). Stimulus perception in bacterial signal-transducing histidine kinases.. Microbiol Mol Biol Rev.

[28] Menke M, Berger B, Cowen L (2010). Markov random fields reveal an N-terminal double beta-propeller motif as part of a bacterial hybrid two-component sensor system.. Proc Natl Acad Sci U S A.

[29] Anderson K. L, Salyers A. A (1989). Genetic evidence that outer membrane binding of starch is required for starch utilization by Bacteroides thetaiotaomicron.. J Bacteriol.

[30] Goodman A, Kallstrom G, Faith J. J, Reyes A, Moore A (2011). Extensive personal human gut microbiota culture collections characterized and manipulated in gnotobiotic mice.. Proc Natl Acad Sci U S A.

[31] Holdeman L. V, Cato E. D, Moore W. E. C (1977). Anaerobe laboratory manual.

[32] Salyers A. A, Bonheyo G, Shoemaker N. B (2000). Starting a new genetic system: lessons from bacteroides.. Methods.

[33] Spellman M. W, McNeill M, Darvill A. G, Albersheim P, Henrick K (1983). Isolation and characterization of 3-C-carboxy-5-deoxy-lyxlose. A naturally occurring branched chain, acidic monosaccharide.. Carbohydrate Research.

[34] Xu J, Mahowald M. A, Ley R. E, Lozupone C. A, Hamady M (2007). Evolution of symbiotic bacteria in the distal human intestine.. PLoS Biol.

[35] Bolam D. N, Xie H, Pell G, Hogg D, Galbraith G (2004). X4 modules represent a new family of carbohydrate-binding modules that display novel properties.. J Biol Chem.

[36] Altschul S. F, Madden T. L, Schaffer A. A, Zhang J, Zhang Z (1997). Gapped BLAST and PSI-BLAST: a new generation of protein database search programs.. Nucleic Acids Res.

[37] Eddy S. R (2009). A new generation of homology search tools based on probabilistic inference.. Genome Inform.

