# Supplementary material for: Recognition and Degradation of Plant Cell Wall Polysaccharides by Two Human Gut Symbionts
Source: PLoS Biol. 2011 Dec 20;9(12):e1001221. doi: 10.1371/journal.pbio.1001221 (PMC3243724; doi:10.1371/journal.pbio.1001221)
Supplement: Text S1 — Additional Results and Discussion of the B. thetaiotaomicron and B. ovatus plant cell wall degrading mechanisms. (DOC) [file pbio.1001221.s001.doc]

**Results and Discussion**

**Exclusivity of *B. ovatus* for barley β-glucan**

*B. ovatus* was capable of growth on barley β-glucan (a β-glucan with a ~3:1 ratio of β1,4 to β1,3 linkages), but did not grow on lichenin (a β-glucan with a ~2:1 ratio of β1,4 to β1,3 linkages) or laminarin (a β-glucan with a ~3:1 ratio of β1,3 to β1,6 linkages). Taken together these data indicate that *B. ovatus* has a finely tuned capacity for β-glucan sensing and degradation: it is incapable of degrading the uniform β1,4-glucosidic linkages that occur in cellulose and cellohexaose, but is capable of degrading barley β-glucan, which has a slightly altered structure (*i.e*., introduction of a β1,3 linkage every fourth residue). If the ratio of β−glucosidic linkages is further skewed towards β1,3 as in lichenin, the ability to use the substrate for growth is lost, suggesting that the sensory, enzymatic and/or binding functions that target barley β-glucan are highly specific. This specificity has likely been adapted and permuted for utilizing other substrates, as we have also identified different *Bacteroides* species capable of using lichenin or laminarin to the exclusion of other β-glucans (N. Pudlo and E. Martens, unpublished).

**Oligosaccharide specificity of the *B. thetaiotaomicron* arabinan HTCS.**

Growth of *B. thetaiotaomicron* on α-arabinan (mainly linear α1,5-linked L-arabinofuranosyl units with α2 or α3 arabinose branching) activates four different PULs (**Fig. 1**, main text), which between them are linked to genes encoding five different HTCS (BT3049, BT0366, BT4673, BT4178 and BT4182). To further test our hypothesis that individual PULs have evolved to target specific pectic substructures, we examined the ligand specificity of the periplasmic domains of each of these HTCS, except for BT4673, which could not be expressed in *E. coli*. The data showed that BT0366, but none of the other HTCS, bound specifically to linear α1,5-linked arabino-oligosaccharides of 6 sugars or more, with affinity increasing up to arabino-octaose (**Table 3**, main text), supporting the conclusion that BT0366 is the regulator of the primary arabinan PUL. This HTCS was also able to interact with both linear and branched arabinan. These findings are consistent with the view that activation of the other three PULs when *B. thetaiotaomicron* was grown on arabinan was due to the presence of non-arabinose sugars in the arabinan preparation, and that activation of the BT0366-associated PUL (*BT0348-69*) by pectic galactan reflected the presence of arabinohexaose (or larger) substructures within the polysaccharide (either as side chains or contaminants). Interestingly, the binding of BT0366 to arabino-oligosaccharides appeared to be a multi-phasic event with two obvious equilibria occurring over the course of the isothermal titration calorimetry assay, and the integrated heats fit well to the default 2-site binding model in Origin v7.0 (**Fig. S7**). This pattern of multi-site binding was also observed with the *B. ovatus* mannan sensor BACOVA_02097, but not the other two HTCS analyzed here (**Table 3** and **Fig. S7**).

**Oligosaccharide specificity of the *B. ovatus* xylan HTCS**

Three different HTCS genes, *BACOVA_03437, BACOVA_03441* and *BACOVA_04394* are linked to the two discrete xylan-activated PULs in *B. ovatus* (**Fig. S1C**). The former two genes are both part of a large PUL spanning *BACOVA_03417-50*; whereas, the latter is adjacent to a smaller PUL encompassing *BACOVA_04385-93*. ITC analysis of the ligand specificity of the periplasmic domains of these HTCS could only be performed with BACOVA_04394, as expression trials with the other two HTCS failed to produce any recombinant protein in *E. coli*. The data show that BACOVA_04394 bound to linear β1,4 linked xylooligosaccharides composed of two or more sugar units (up to xylohexaose), with a strong preference for xylotetraose over shorter or longer oligosaccharides (approximately an order of magnitude difference in affinity to the next closest oligosaccharide X5; **Table 3**). No interaction with polymeric oat spelt xylan was detected. The binding of BACOVA_04394 to undecorated xylo-oligosaccharides indicates that the adjacent PUL is activated when any type of β1,4 linked xylan is present, irrespective of additional substitutions. More complex xylan structures may be targeted by this PUL, but will be broken down to linear xylo-oligosaccharides at some point during their de-polymerization. Activation of the other larger xylan PUL may be by substituted oligosaccharides, as both wheat and oat xylans contain a number of different side chains appended to the core xylan backbone, including arabinose and glucuronic acid.

**Oligosaccharide specificity of the *B. ovatus* β-glucan HTCS**

Mixed linkage (β1,4-1,3) barley β-glucan activated two discrete PULs in *B. ovatus* (**Fig. 2**, main text and **Fig. S1C**), although only the most highly upregulated PUL (*BACOVA_02741-47*) was linked to a HTCS gene (*BACOVA_02740*). Screening the periplasmic domain of BACOVA_02740 against a range of mixed linkage gluco-oligosaccharides by ITC revealed that the protein bound to the β4-3 linked trisaccharide as well as β4-4-3-linked tetrasaccharide with approximately the same affinity, but had a strong preference (~10-fold higher *K*_a_) for the β4-3-4 tetrasaccharide. The HTCS displayed no detectable interaction with the β3-4 linked trisaccharide, β1-3 laminari-oligosaccharides or β-glucan polysaccharide, and although some binding to the mixed linkage pentasaccharide (β3-4-4-4) was observed, the affinity was too low to quantify. Interestingly, BACOVA_02740 bound cellobiose, albeit weakly, but not larger cellooligosaccharides, providing a likely explanation for the ability of *B. ovatus* to utilize this disaccharide.

**Oligosaccharide specificity of the *B. ovatus* galacto- and glucomannan HTCS**

The HTCS gene adjacent to the single PUL activated by both galacto- and glucomannan is *BACOVA_02097* (**Fig. 2**, main text and **FigS1C**). As the shared oligosaccharide signature of both galactomannan (β1,4 linked mannose backbone with α1,6 linked galactose side chains on ~20% of mannoses) and glucomannan (β1,4 linked mannose-glucose backbone, ratio ~1:3 Glc:Man) are β1,4 linked mannoses, we tested the ability of the periplasmic domain of BACOVA_02097 to bind to manno-oligosaccharides from 2-6 sugars in length. The data show that the protein bound oligosaccharides containing 4 or more mannose residues with increasing affinity, but did not interact with mannobiose, mannotriose or galactosylated manno-oligosaccharides (**Table 3**, main text). BACOVA_02097 also displayed binding to galacto- and glucomannan.

***B. thetaiotaomicron*  transcriptional responses to galacto-oligosaccharide cues**

We used qPCR to explore the specificity of the four *B. thetaiotaomicron* PULs upregulated in response to pectic galactan, the backbone of which is a homopolymer of β1,4 linked galactose. *BT0362* was included as a control since although this gene was from a PUL activated by pectic galactan, we had already shown the PUL actually responded to arabino-oligosaccharide sidechains/contaminants in the galactan preparation used. As the only commercially available β1,4 linked galacto-oligosaccharide is galactobiose, we grew *B. thetaiotaomicron* on this disaccharide as well as pectic galactan and compared expression of the *sus*C markers from each of the four putative galactan PULs (**Fig. 1**). The data show that the *susC*-like gene (*BT4671*) from the *BT4667-72*-associated PUL was modestly upregulated when *B. thetaiotaomicron* was grown on galactobiose (**Fig. 5**, main text), supporting the transcriptomic and gene knockout data that indicate *BT4667-72* is responsive to pectic galactan. However, although galactobiose activated this PUL, the level of upregulation by the disaccharide was an order of magnitude lower than observed for the polysaccharide, suggesting that the preferred signal for BT4673 HTCS is a larger galacto-oligosaccharide.
