# Supplementary material for: Recognition and Degradation of Plant Cell Wall Polysaccharides by Two Human Gut Symbionts
Source: PLoS Biol. 2011 Dec 20;9(12):e1001221. doi: 10.1371/journal.pbio.1001221 (PMC3243724; doi:10.1371/journal.pbio.1001221)
Supplement: Table S1 — B. thetaiotaomicron genes with altered expression in vitro during growth on plant and host glycans or in vivo in mono-associated gnotobiotic mice fed a plant glycan rich diet. Genes are separated according to the Venn diagram sectors delineated in Figure S4. Values shown are the mean fold differences compared to expression during growth in MM-G; only fields with values ≥10-fold are shown. Upregulated genes are shown in green; downregulated genes are shown in red. Empty cells indicate a fold-change value <10. (PDF) [file pbio.1001221.s010.pdf]

Table S1. ***B. thetaiotaomicron*** **genes with altered expression** ***in vitro*** **during growth on plant and host glycans or** ***in vivo*** **in mono-associated gnotobiotic mice fed a plant glycan rich diet**

Genes are separated according to the Venn diagram sectors delineated in Figure S1. Values shown are the mean fold differences compared to expression during growth in MM-G; only fields with values ≥10-fold are shown.
Upregulated gene are shown in green; downregulated genes are shown in red. Empty cells indicate a fold-change values <10.

Gene	Gene description	arabinan	arabino galactan	pectic galactan	homogal acturonan	rhamnogal acturonan I	rhamnogal acturonan II	pullulan	neutral O-glycans	α-mannan	chondroitin sulfate	heparin	hyaluronan	adult plant rich	adult simple sugar	suckling
**Upregulated:**																
**Sector 1**																
BT0292	hypothetical protein													15.4		42.0
BT0294	hypothetical protein													94.1	176.2	43.7
BT0317	susC-like													116.3	223.3	39.0
BT0318	susD-like													18.7	25.0	
BT0483	susC-like													14.5	12.6	
BT0484	susD-like		10.7			19.2				16.9		15.2		11.7	15.3	48.7
BT0683	Glycoside hydrolase family 97													16.0		
BT0693	hypothetical protein													59.2	53.5	40.2
BT0722	conserved hypothetical protein, putative surface protein, function unknown													10.4		
BT0754	susC-like													13.8		
BT0763	hypothetical protein													23.9		
BT0764	hypothetical protein													18.9		
BT0765	putative protease													23.3	204.8	116.7
BT0865	Carbohydrate binding module family 32													42.5	394.7	246.9
BT0866	susD-like													21.4	282.1	145.4
BT0867	susC-like													10.1	15.1	
BT0901	putative universal stress protein UspA													19.4		
BT1008	xylanase													66.0		
BT1009	dihydroorotate dehydrogenase								74.9			14.0		139.9	232.7	457.1
BT1034	putative signal transducer								17.7					75.1	233.7	104.2
BT1047	susD-like													21.1	16.1	
BT1072	hypothetical protein													17.0	23.0	
BT1073	hypothetical protein													21.9	21.9	
BT1074	hypothetical protein													18.2	24.1	
BT1196	pyruvate carboxylase subunit B)													15.7		14.8
BT1280	susC-like								22.7				13.0	176.7	12.5	18.8
BT1283	hypothetical protein													17.2	78.5	20.9
BT1287	hypothetical protein													22.9	22.9	40.0
BT1486	hypothetical protein													41.0	33.1	59.6
BT1487	hypothetical protein													43.7	37.2	82.3
BT1488	hypothetical protein													26.2	16.8	26.1
BT1489	vitamin B12 receptor, outer membrane													16.3	13.4	
BT1492	tyrosine phenol-lyase													68.1	10.4	
BT1530	putative outer membrane protein OprM precursor													91.7		
BT1533	hypothetical protein													12.0	93.8	38.9
BT1619	susC-like													11.5	72.8	39.2
BT1653	hypothetical protein													17.1	67.5	124.6
BT1655	hypothetical protein													10.7	65.5	49.4
BT1656	putative transcriptional regulator													14.3	54.0	152.1
BT1718	putative 2-aminoethylphosphonate pyruvate aminotransferase													10.6		
BT1904	transcriptional regulator, AraC-type													31.9	60.5	57.3
BT1950	Fe3+ ABC transporter, ATP-binding protein													29.2	27.5	25.0
BT1951	Fe3+ ABC transporter, permease													48.3	68.7	51.1
BT1952	Fe3+ ABC transporter, periplasmic iron-binding protein													40.1	44.7	44.3
BT1953	putative TonB-linked outer membrane receptor													31.0	53.2	35.9
BT1954	putative surface layer protein													48.0	58.7	52.9
BT1955	putative cell wall biogenesis protein													36.0	102.3	77.0
BT1956	putative cell surface protein													21.4	24.3	
BT1973	NAD-specific glutamate dehydrogenase													12.5		11.0
BT2038	cation efflux system protein													12.9		
BT2040	hypothetical protein													16.2		
BT2087	hypothetical protein													17.2	14.2	12.4
BT2094	TonB-dependent receptor													12.6	11.9	

BT2383	RNA polymerase ECF-type sigma factor				11.3	49.7	53.2
BT2395	hypothetical protein				19.9	19.8	11.9
BT2404	L-asparaginase I				17.2	21.3	
BT2551	phosphate butyryltransferase				20.8	19.1	
BT2552	putative butyrate kinase				18.4	69.1	22.8
BT2569	RNA polymerase ECF-type sigma factor				29.3	65.7	14.9
BT2690	histidine ammonia-lyase				14.8	30.5	12.2
BT2691	methenyltetrahydrofolate cyclohydrolase				35.1	36.3	
BT2692	imidazolonepropionase				31.0	63.9	19.6
BT2693	formiminotransferase-cyclodeaminase				23.4	28.8	
BT2694	putative urocanate hydratase				10.6	13.9	
BT2762	TonB				40.3	25.4	
BT2778	RNA polymerase ECF-type sigma factor				96.0	47.3	
BT2779	3-demethylubiquinone-9 3-methyltransferase				24.5	22.4	
BT2784	hypothetical protein				13.4	16.2	
BT2785	hypothetical protein				12.8	35.6	62.1
BT2804	ribokinase				34.4		
BT2893	susD-like				14.2		
BT2894	susC-like	34.8	10.4	12.6	313.2		
BT2896	hypothetical protein				11.4		
BT2899	hypothetical protein				21.5		
BT3043	Glycoside hydrolase family 30				16.6		
BT3048	hypothetical protein				14.4		
BT3188	hypothetical protein				11.4		
BT3189	hypothetical protein				10.1		
BT3190	putative RNA polymerase ECF-type sigma factor				164.0	77.6	58.0
BT3259	hypothetical protein				18.9	37.6	
BT3436	hypothetical protein				25.1	53.4	
BT3437	hypothetical protein				27.3	138.7	
BT3438	hypothetical protein				139.3	201.8	
BT3441	hypothetical protein				18.3	16.1	
BT3475	susC-like				10.1		
BT3525	hypothetical protein				15.3		
BT3650	hypothetical protein				50.3		
BT3651	hypothetical protein				70.4		
BT3652	hypothetical protein				89.6		
BT3653	hypothetical protein				10.3		
BT3769	hypothetical protein				16.8		
BT3822	hypothetical protein				10.5		
BT3874	hypothetical protein				45.9	104.8	125.0
BT3983	susC-like				60.0	90.7	102.1
BT3984	susD-like				42.3	223.8	203.4
BT3985	hypothetical protein				102.8	243.8	270.0
BT3986	putative patatin-like protein				54.9	133.1	147.5
BT3987	Glycoside hydrolase family 18				43.3	103.9	136.4
BT3988	putative peptidoglycan bound protein				19.1	47.8	42.4
BT4038	susD-like				17.0	32.2	23.2
BT4040	Carbohydrate binding module family 32				16.3	50.4	10.4
BT4356	putative anti-sigma factor				61.8	400.8	47.4
BT4358	susD-like				14.6		18.5
BT4404	susC-like				27.1	33.1	43.0
BT4405	susD-like				18.3	12.0	16.2
BT4406	hypothetical protein				12.4	17.4	
BT4465	conserved hypothetical protein, putative outer membrane protein				30.9	13.1	
BT4647	RNA polymerase ECF-type sigma factor				17.2		
BT4649	hypothetical protein				18.5	17.7	11.4
BT4689	Glycoside hydrolase family 13				20.6		
BT4708	susD-like				25.4		
BT4709	Glycoside hydrolase family 18				18.5		
BT4710	hypothetical protein				21.1		
BT4711	hypothetical protein				13.3		11.5
BT4712	hypothetical protein						

**Sector 2**																
BT0319	hypothetical protein													162.6	346.7	101.6
BT0348	Glycoside hydrolase family 51	41.2	12.6	25.6			31.8							18.1		
BT0349	hypothetical protein	49.2	11.6	31.3			47.6							16.0		19.1
BT0360	Glycoside hydrolase family 43	315.8		156.7										67.6		13.1
BT0361	susD-like	381.0		210.0										156.1	28.2	46.6
BT0362	susC-like	234.0		118.8										57.2		11.4
BT0365	hypothetical protein	1222.0		676.7										780.7	29.0	100.0
BT0366	hybrid two-component system (HTCS)	35.1		16.9										10.9		
BT0367	Glycoside hydrolase family 43	1723.0		944.6	12.1								12.0	200.7		16.1
BT0368	Glycoside hydrolase family 51	52.5		30.7										20.3		
BT0369	Glycoside hydrolase family 43	77.2		43.3										19.4		15.1
BT0565	putative heat shock protein													15.2	11.7	
BT0787	succinyl-CoA synthetase alpha chain													15.3	30.4	
BT0788	succinyl-CoA synthetase beta chain													11.2	35.2	
BT0978	ECF-type sigma factor			17.9		146.2	251.3							22.3		15.4
BT1029	susC-like			14.8		551.4	495.0							61.0		
BT1142	hypothetical protein		10.2											37.4	16.9	
BT1448	biotin carboxyl carrier protein													26.8	58.1	15.6
BT1449	biotin carboxylase													36.4	52.9	18.6
BT1450	propionyl-CoA carboxylase beta chain													38.6	51.1	16.8
BT1491	hypothetical protein													24.6	12.2	26.0
BT1535	ABC transporter ATP-binding protein	12.0		11.6										134.0		16.6
BT1757	fructokinase													23.1		10.5
BT1758	glucose/galactose transporter				16.9									96.9	19.2	38.9
BT1760	Glycoside hydrolase family 32				21.9									72.5	50.8	90.4
BT1761	hypothetical protein				22.9									83.0	21.9	36.1
BT1763	susC-like				13.7									42.2	14.5	24.4
BT1765	Glycoside hydrolase family 32				13.3									40.8	10.0	21.6
BT2131	hypothetical protein		10.6											19.8	278.8	273.3
BT2500	hypothetical protein													150.7	19.4	
BT3046	susC-like	22.2												43.6		
BT3047	hypothetical protein	184.1	41.8	33.6	30.2		28.8		10.8				24.3	365.2	22.0	61.0
BT3082	Glycoside hydrolase family 32													18.2		
BT3614	putative oxidoreductase					54.2	103.8							23.1		
BT3655	Glycoside hydrolase family 43		24.8											17.4		
BT3656	Glycoside hydrolase family 43		20.9											19.1		
BT3681	susD-like	88.3	619.8		58.9	20.4	11.4					13.6		33.7		
BT3699	outer membrane protein SusF							43.7						11.5		24.2
BT3700	outer membrane protein SusE							54.2						15.9	17.1	49.3
BT3702	susC , outer membrane protein involved in starch binding							29.2						11.3		17.6
BT3703	susB, Glycoside hydrolase family 97							26.4						10.9		23.1
BT3704	susA, Glycoside hydrolase family 13							77.8						11.2		16.5
BT3735	hypothetical protein													13.9		14.8
BT3764	L-rhamnose isomerase	163.7		147.7	55.0	245.6	263.4							33.8	13.4	
BT4113	susD-like			53.0	92.6	49.3	50.7							12.5	38.1	74.4
BT4114	susC-like			68.2	136.1	82.7	73.7							11.7	20.4	34.2
BT4120	hypothetical protein			61.0	187.9		148.2							36.3		
BT4121	susC-like			53.6	224.5		145.6							14.5		
BT4122	susD-like			187.5	755.4		404.4							209.1	28.7	14.4
BT4153	Glycoside hydrolase family 28	52.9		45.3	13.2	70.7	13.7							13.5		
BT4163	hypothetical protein	101.3		72.9	36.3	186.5	46.2							20.7		
BT4164	susC-like	88.2		68.0	34.0	135.7	38.3							10.6		
BT4165	susD-like	114.9		83.3	37.6	128.0	50.8							16.1		
BT4166	putative lipoprotein	69.0		48.7	29.5	92.8	29.0							10.9		
BT4167	hypothetical protein	68.6		53.3	26.3	71.3	27.1							11.4		
BT4169	susD-like	77.1		67.0	35.9	93.8	38.5							16.6		
BT4170	Polysaccharide lyase family 9	34.5		26.1	12.9	47.4	14.8							10.7		
BT4172	hypothetical protein	52.1		45.4	16.3	65.4	19.7							10.9		
BT4175	Polysaccharide lyase family 11	73.6		57.0	20.0	140.7	28.2							18.3		
BT4176	Glycoside hydrolase family 105	35.2		25.6	11.4	48.1	12.4							10.0		
BT4667	Glycoside hydrolase family 2	13.8		123.4	25.9									78.6		24.0
BT4668	Glycoside hydrolase family 53	13.7		140.7	21.4									136.6	16.9	46.9
BT4670	susD-like			104.6										72.9		14.0
BT4672	hypothetical protein	19.8		246.9	16.3									75.6		19.2

Sector 3

BT0111	hypothetical protein		16.6							
BT0112	putative permease		13.6							
BT0113	hypothetical protein		10.0							
BT0115	arsenate reductase		24.7							
BT0116	arsenical pump-driving ATPase		14.4							
BT0117	arsenical resistance operon trans-acting repressor		15.2							
BT0118	putative cytochrome c biogenesis protein		27.5							
BT0119	hypothetical protein		16.9							
BT0120	hypothetical protein		29.3							
BT0121	transcriptional regulator, ArsR family		10.6							
BT0198	hypothetical protein	10.8								
BT0262	hypothetical protein		66.1							
BT0263	hypothetical protein		42.0							
BT0264	Glycoside hydrolase family 43		1860.0							
BT0265	Glycoside hydrolase family 43		465.4							
BT0266	hypothetical protein		225.2					13.1		17.4
BT0268	susC-like		72.7							
BT0269	susD-like		222.6							
BT0270	hypothetical protein		214.8							
BT0271	hypothetical protein		121.0							
BT0272	susC-like		123.1							
BT0273	susD-like		130.9							
BT0274	hypothetical protein		144.8							
BT0275	hypothetical protein		242.6							
BT0276	hypothetical protein		145.0							
BT0277	hypothetical protein		133.0							
BT0278	hypothetical protein		109.4							
BT0279	hypothetical protein		155.3						11.3	
BT0280	transposase for insertion sequence element ISRM3		11.7							
BT0284	putative peptidoglycan binding protein (LPXTG motif)		17.0							
BT0285	putative tolQ-type transport protein		17.2							
BT0286	hypothetical protein		16.5							
BT0287	putative biopolymer transmembrane protein		21.4							
BT0288	hypothetical protein		22.4							
BT0290	Glycoside hydrolase family 35		93.7							
BT0338	hypothetical protein					76.5	33.8			
BT0339	Glycoside hydrolase family 31					107.5	142.6	10.6		
BT0340	trimethylamine corrinoid protein 2 (TCP 2)					42.4	68.9	15.6		
BT0341	Na+/glucose cotransporter					211.6	360.8	11.3		
BT0342	hypothetical protein					47.6	143.2	10.7		
BT0343	methylcobamide:CoM methyltransferase mtbA					82.1	154.5	10.1		
BT0350	xylulose kinase (xylulokinase)	22.0		14.7			21.0			
BT0352	hypothetical protein	11.7								
BT0353	putative sugar epimerase/aldolase	10.2					10.7			
BT0354	hypothetical protein	12.9					14.7			
BT0355	Na+/glucose cotransporter	12.9		10.2			13.7			
BT0356	aldose 1-epimerase precursor	11.7		10.7			12.6			
BT0363	susD-like	471.2		256.2				98.6	13.6	26.9
BT0364	susC-like	297.4		151.6				177.0		18.4
BT0434	hypothetical protein					10.4				
BT0435	hypothetical protein					10.1				
BT0437	N-acylglucosamine 2-epimerase					10.1			11.0	
BT0438	Glycoside hydrolase family 89					14.1			10.6	
BT0442	glycerophosphoryl diester phosphodiesterase					10.9			10.0	
BT0622	Na+-transporting NADH:ubiquinone oxidoreductase, Electron transport complex protein rnfA				11.2					
BT0823	uronate isomerase					20.5				
BT0824	transcriptional regulator (LacI family)				19.9	10.4	14.3			

BT0854	hypothetical protein												11.3
BT0977	hypothetical protein					59.9	167.5						
BT0978	ECF-type sigma factor						251.3						
BT0979	hypothetical protein						14.1						
BT0980	Polysaccharide lyase family 1						11.5						
BT0982	hypothetical protein					20.5	62.1						
BT0983	Glycoside hydrolase family 2					14.2	24.0						
BT0984	hypothetical protein						22.9						
BT0985	putative sialic acid-specific acetylesterase II					12.3	36.7						
BT0986	Glycoside hydrolase family 106					11.2	17.5						
BT0992	Glycoside hydrolase family 2					77.9	115.6				35.4		
BT0993	Glycoside hydrolase family 2					22.7	66.6				10.2		10.3
BT0996	Glycoside hydrolase family 2					171.1	262.3						
BT0997	hypothetical protein					47.6	63.9						
BT1001	Glycoside hydrolase family 78					24.8	64.4				36.9		
BT1002	hypothetical protein					55.8	83.7						
BT1003	hypothetical protein					35.6	56.4						
BT1010	Glycoside hydrolase family 95					68.7	147.7				15.8		
BT1011	Glycoside hydrolase family 105					140.4	210.7				16.4		
BT1012	hypothetical protein					82.6	165.1				17.1		
BT1013	Glycoside hydrolase family 78					77.0	118.0						
BT1017	hypothetical protein						28.2						
BT1018	Glycoside hydrolase family 28						24.1						
BT1019	Glycoside hydrolase family 78					13.8	33.4						
BT1020	hypothetical protein					105.8	264.8				11.4		
BT1021	Glycoside hydrolase family 43					23.0	60.0				15.5		
BT1022	hypothetical protein					25.1	36.8						
BT1023	Polysaccharide lyase family 1					31.9	49.8						
BT1026	hypothetical protein						36.4						
BT1027	hypothetical protein						22.8						
BT1028	susD-like					78.1	120.8						
BT1029	susC-like						495.0						
BT1030	hypothetical protein			13.8		676.1	457.3				14.4		
BT1031	hypothetical protein					84.5	123.1						
BT1230	hypothetical protein		19.1			25.1	77.1	11.1	15.4		50.2	15.7	
BT1415	cytochrome c biogenesis protein ccsA				11.4		16.0						
BT1417	cytochrome C552 precursor				14.4		16.5						14.1
BT1418	quinol oxidase				13.6		16.4						20.3
BT1518	hypothetical protein												15.3
BT1519	N-acetylmuramoyl-L-alanine amidase											14.1	17.8
BT1682	susD-like					182.2	115.6						13.4
BT1683	susC-like					148.2	106.8						12.1
BT1759	Glycoside hydrolase family 32				24.7						92.3	29.1	56.5
BT1762	susD-like				70.0						396.7	61.9	80.2
BT1884	cold shock protein, putative DNA-binding protein	15.2											
BT1885	putative ATP-dependent RNA helicase	10.3											
BT1903	hypothetical protein											11.9	
BT2096	putative transcriptional regulator			10.1									
BT2097	hypothetical protein	17.9		32.5			11.6						
BT2118	transporter, AcrB/D/F family			10.0								15.4	
BT2254	Polysaccharide lyase family 10			10.2	30.1								
BT2680	Glycoside hydrolase family 2	31.2		22.8	10.4	50.7	12.8						
BT2813	sodium-dependent multivitamin transporter					201.7	184.0				18.4		
BT2814	dihydrodipicolinate synthase					275.5	219.5				13.0		
BT3044	hypothetical protein	32.6									88.3		14.7
BT3045	susD-like	19.4									256.9	21.0	59.5
BT3086	Glycoside hydrolase family 31			13.9	17.4	17.0	10.3			14.5			
BT3420	hypothetical protein		133.4								10.1		
BT3421	hypothetical protein		59.6										
BT3422	hypothetical protein		54.8										
BT3423	hypothetical protein		89.6										
BT3424	hypothetical protein		58.1										
BT3425	hypothetical protein		17.9										
BT3615	hypothetical protein					87.2	121.7				34.2		10.8

BT3616	fucose permease					49.7	80.0								
BT3617	sorbitol dehydrogenase					42.2	111.0						12.6	10.9	
BT3654	Glycoside hydrolase family 35		23.7										25.1		
BT3657	Glycoside hydrolase family 51		20.9										29.6		15.0
BT3658	hypothetical protein	28.3	74.2		10.3								43.9		12.6
BT3662	Glycoside hydrolase family 43					13.0	21.3							29.7	
BT3665	Glycoside hydrolase family 29					11.5	23.9							28.7	
BT3669	hypothetical protein					18.5	53.1							45.6	
BT3672	hypothetical protein					75.0	123.1							65.8	
BT3674	hypothetical protein		19.7										16.4	10.8	22.6
BT3675	Glycoside hydrolase family 43	10.3	33.1												
BT3676	hypothetical protein		33.1												
BT3677	hypothetical protein		21.5												
BT3679	conserved hypothetical protein, with a conserved domain	10.9	110.5		10.4								13.5		
BT3680	susC-like	54.6	625.5		50.6								45.3		10.4
BT3682	hypothetical protein	33.4	170.8		19.8								77.0	22.4	44.8
BT3683	Glycoside hydrolase family 16	12.2	83.6												
BT3685	Glycoside hydrolase family 43		12.2												
BT3686	hypothetical protein		13.9												
BT3698	alpha-amylase, susG							53.9							22.6
BT3701	susD							33.0							12.4
BT3747	hypothetical protein		36.6												
BT3763	rhamnulose kinase/L-fuculose kinase	497.1		523.4	184.0	602.2	827.6					11.2	28.7		
BT3765	L-rhamnose/H+ symporter	371.2		305.7	105.3	433.8	471.2						113.0	19.6	
BT3766	rhamnulose-1-phosphate aldolase	350.4		244.1	102.2	451.4	339.3						229.0	103.5	58.6
BT3767	lactaldehyde reductase	10.5				12.9	14.8								
BT4105	hexuronate transporter			10.1	17.1	16.1	12.4								
BT4109	Carbohydrate esterase family 8			117.0	374.2	51.8	140.9			19.4	18.2	12.0			
BT4110	Carbohydrate esterase family 8			37.5	90.5	21.1	56.9							10.2	13.7
BT4112	hypothetical protein			64.5	138.2	46.3	57.9							12.0	28.8
BT4115	Polysaccharide lyase family 1			36.5	73.6	11.0	24.4								
BT4116	Polysaccharide lyase family 1			37.4	127.8	39.5	46.2								
BT4119	Polysaccharide lyase family 1			36.5	135.5		89.1						11.6		14.7
BT4123	Glycoside hydrolase family 28			37.6	150.8		42.0								
BT4124	hybrid two-component system (HTCS)				24.2										
BT4145	Glycoside hydrolase family 106	14.0		13.9		20.7									
BT4146	Glycoside hydrolase family 28	21.5		17.5		29.8									
BT4147	hypothetical protein	21.2		19.1		19.2									
BT4148	hypothetical protein	22.5		18.5		34.9									
BT4149	Glycoside hydrolase family 28	46.7		38.4	16.0	64.3	20.1								
BT4150	Carbohydrate esterase family 12	37.5		33.8	15.4	73.3	13.1								
BT4151	Glycoside hydrolase family 2	242.3		219.9	91.1	568.6	75.0						26.3	17.6	23.4
BT4152	Glycoside hydrolase family 42	212.2		151.8	44.3	178.7	60.2						33.6		
BT4154	Carbohydrate esterase family 4	22.7		17.8		16.5									
BT4155	Glycoside hydrolase family 28					11.0									
BT4156	Glycoside hydrolase family 2	10.2				10.7									
BT4158	hypothetical protein	70.2		52.7	24.8	132.5	36.6								
BT4159	hypothetical protein	24.8		23.2		88.9	19.7								
BT4160	beta-galactosidase precursor	31.7		36.3	12.4	77.6	51.2						12.7		
BT4168	susC-like	39.8		28.8	19.6	60.3	17.4								
BT4171	hypothetical protein	49.6		36.2	24.2	77.5	15.7								
BT4173	Carbohydrate esterase family 12	19.9		14.8		64.0	10.6								
BT4174	Glycoside hydrolase family 105	31.0		22.3		62.2	13.7								
BT4177	hypothetical protein	64.6		48.6	20.2	68.1	23.2								
BT4181	Glycoside hydrolase family 2	17.4													
BT4183	Polysaccharide lyase family 9	53.9		27.6	13.5	55.5	21.2								
BT4185	Glycoside hydrolase family 43			12.8	33.0		12.1								
BT4187	Glycoside hydrolase family 28			35.2	75.1	37.4	40.6								
BT4227	hypothetical protein							10.4					11.2	53.2	19.3
BT4669	hypothetical protein	130.9		881.2	140.3	24.8					13.1	12.6	404.4	34.4	144.9
BT4671	susC-like	27.5		192.0	19.2								56.2		20.2
**Sector 4**															
BT0506	Glycoside hydrolase family 20								11.2				18.2	34.8	52.9
BT0791	hypothetical protein						18.5			13.4			13.8		
BT1036	hypothetical protein								37.7				35.4	153.4	39.5

BT1037	hypothetical protein							54.4					50.1	202.3	29.4
BT1038	hypothetical protein							62.0					79.5	271.1	51.8
BT1039	susD-like							37.2					62.4	198.8	42.7
BT1040	susC-like							48.0					39.3	190.7	39.4
BT1042	susC-like							20.4					19.2	127.6	238.0
BT1043	susD-like							17.8					16.8	98.1	182.3
BT1044	Glycoside hydrolase family 18							20.7					22.0	161.8	275.4
BT1045	hypothetical protein							13.1					10.8	51.0	100.3
BT1046	susC-like							78.3					288.2	158.8	82.1
BT1048	Glycoside hydrolase family 18							11.5					43.2	191.2	96.1
BT1534	hypothetical protein							22.6					132.7		14.7
BT1554	alanine dehydrogenase										10.8		33.5	36.0	
BT2559	susD-like								11.9				16.2	20.5	
BT2561	putative anti-sigma factor								11.2				20.7	12.7	
BT2818	susC-like							368.2					22.6	37.5	14.8
BT2820	susC-like							4216.0					230.1	46.8	
BT2821	susD-like							433.0					32.5	87.3	16.5
BT2822	hypothetical protein							139.5					11.6	30.1	
BT2823	hypothetical protein							3671.0					206.2	508.4	81.9
BT3778	hypothetical protein								113.5				14.2	34.0	10.7
BT3790	hypothetical protein						17.0		139.3				11.5		
BT3860	hypothetical protein								21.7				36.8	16.0	
BT4294	hypothetical protein							24.3					12.1	92.9	109.8
BT4295	putative chitobiase							30.8				13.5	23.9	406.3	488.3
BT4296	hypothetical protein							32.8				17.9	21.8	109.2	126.3
BT4297	susD-like							37.3				16.1	23.9	77.9	76.8
BT4298	susC-like							33.5				16.5	23.4	472.6	415.8
BT4299	hypothetical protein							38.0				19.0	20.6	126.4	176.8
BT4407	hypothetical protein							12.6					34.1	37.6	92.1
BT4648	hypothetical protein										11.8		55.3	13.4	
**Sector 5**															
BT1536	ABC transporter permease	33.6		32.7	16.8				11.7				207.2	45.6	15.6
BT2167	elongation factor G		15.1	11.6	20.1	12.6	10.1	31.6			37.0	11.5	79.2	186.5	101.1
BT3221	hypothetical protein			86.3		17.6		27.0					171.9		
BT3222	hypothetical protein			94.8				15.9					219.9		
BT3223	hypothetical protein			52.5				10.4					123.4		
BT3572	hypothetical protein		14.6					182.1					17.5		
BT3573	hypothetical protein		15.5					297.4					18.1	22.5	
BT3793	hypothetical protein										30.1		56.7	112.8	31.3
BT4108	Glycoside hydrolase family 105	19.1		131.6	300.3	103.2	146.7			28.2	13.6	16.2	16.7	12.9	10.3
BT4384	hypothetical protein								15.9				20.2	10.0	
**Sector 6**															
BT0489	putative gluconate aldolase				11.7	13.7					11.2	14.0			
BT1211	hypothetical protein	10.7					10.9				12.7				
BT1572	RNA polymerase ECF-type sigma factor	31.7		51.1	58.4		54.3			54.7	52.8				
BT1604	hypothetical protein	12.2		21.4	10.4		10.6			15.9	11.7				
BT3231	4-deoxy-L-threo-5-hexosulose-uronate ketol- isomerase				10.9					13.3	19.7	15.4			
BT3571	hypothetical protein		21.7					203.9					13.2		
BT3574	hypothetical protein		17.8					75.6							
BT3575	hypothetical protein							54.7							
BT4106	4-deoxy-L-threo-5-hexosulose-uronate ketol- isomerase				12.2	21.7	11.9			15.3	23.7	35.8			
BT4107	putative phosphotransferase enzyme II, C component	10.3		12.3	14.9	14.2	14.1			30.0	26.3	38.2			
BT4393	hypothetical protein					11.5		14.9			14.7			27.8	14.7
BT4715	non-specific DNA-binding protein Dps	23.4		21.7	21.6		33.0			27.8	22.1				
**Sector 7**															
BT0215	iron uptake regulatory protein										10.3				
BT0459	Glycoside hydrolase family 20							11.2						22.3	15.5
BT0488	2-dehydro-3-deoxygluconokinase											11.2			
BT1049	putative patatin-like protein							20.5					83.4	269.7	119.0
BT1273	L-fucose isomerase							21.6							
BT1274	L-fuculose-1-phosphate aldolase							15.6							

BT1275	L-fuculose kinase			20.3							
BT1276	hypothetical protein			25.4						14.1	17.5
BT1277	L-fucose permease			33.2						11.4	13.3
BT1532	ABC transporter permease			14.2					181.0		21.2
BT1596	putative sulfatase yidJ					11.7	40.9				
BT1626	Glycoside hydrolase family 2			12.1							
BT1627	Glycoside hydrolase family 20			20.0							
BT1628	putative sulfatase yidJ			17.2							
BT1629	hypothetical protein						15.0			18.1	
BT1631	susC-like					10.7	11.7	11.4		14.3	
BT2119	transmembrane protein precursor, possibly involved in transport	10.0	10.1					10.4		11.0	
BT2451	putative pyrogenic exotoxin B			12.2							
BT2560	susC-like				13.8				21.3	25.0	
BT2615	reverse transcriptase				50.0						
BT2617	reverse transcriptase				95.0						
BT2618	two-component system response regulator		12.7		232.3						
BT2619	two-component system sensor histidine kinase				39.8						
BT2620	Glycoside hydrolase family 97		11.3		84.4						
BT2621	hypothetical protein				75.3						
BT2622	Glycoside hydrolase family 67				78.7						
BT2623	Glycoside hydrolase family 76		18.8		168.8				14.6	20.0	17.0
BT2624	hypothetical protein		12.3		93.2						
BT2625	susD-like		17.8		128.8					12.3	
BT2626	susC-like		16.4		128.2					33.4	23.8
BT2627	putative cell surface protein, have conserved domain		18.7		104.7					23.2	14.9
BT2628	hybrid two-component system (HTCS)				55.7						
BT2629	Glycoside hydrolase family 92		15.6		99.0					12.9	
BT2630	conserved hypothetical protein with endonuclease/exonuclease/phosphatase family domain				16.6						
BT2631	Glycoside hydrolase family 76				13.1						
BT2632	hypothetical protein				15.6						
BT2633	hypothetical protein				14.3						
BT2812	alkyl hydroperoxide reductase C22 protein						10.5				
BT2819	susD-like			416.8					27.9	45.8	17.0
BT2824	Glycoside hydrolase family 16			1264.0					79.3	66.5	26.5
BT2825	Glycoside hydrolase family 18			77.7						18.6	12.0
BT2919	susD-like						20.7			10.7	
BT2920	susC-like			14.5			51.8	14.4	44.0	45.3	
BT3233	hypothetical protein						10.1				
BT3324	Polysaccharide lyase family 8					78.5		100.3			
BT3328	hypothetical protein					57.2		71.9			
BT3329	hypothetical protein					160.8		456.4			
BT3330	hypothetical protein					184.7		433.3			
BT3331	susD-like					94.6		171.3			
BT3332	susC-like					120.6		314.6			
BT3333	arylsulfatase A precursor (ASA)					115.2		55.7			
BT3348	Glycoside hydrolase family 88					28.5		74.3			
BT3349	putative sulfatase yidJ					23.0		79.2			
BT3350	Polysaccharide lyase family 8					44.1		52.9			
BT3750	susC-like			132.0					36.1	23.6	12.4
BT3751	outer membrane protein			15.8							
BT3752	susD-like			19.1							
BT3753	endo-beta-N-acetylglucosaminidase F2 precursor			12.7							
BT3754	hypothetical protein			13.1						10.1	
BT3773	Glycoside hydrolase family 92				10.7						
BT3774	Glycoside hydrolase family 38				20.0						
BT3775	hypothetical protein				52.0				41.4	14.7	
BT3776	hypothetical protein				34.1				12.3	16.1	
BT3777	hypothetical protein				70.6				54.2	86.6	27.9
BT3779	hypothetical protein		10.5		41.4				50.1	43.0	
BT3780	hypothetical protein		10.3		88.5						

BT3781	hypothetical protein							14.0						
BT3782	Glycoside hydrolase family 76							23.0						
BT3784	Glycoside hydrolase family 92					20.2		140.1					66.7	44.1
BT3786	hybrid two-component system (HTCS)							28.5						
BT3787	hypothetical protein					12.9		119.5						
BT3788	susC-like					19.3		161.8						11.4
BT3789	susD-like					12.2		96.7						
BT3791	hypothetical protein							56.6						
BT3792	Glycoside hydrolase family 76					12.6		86.6						
BT3854	susC-like							41.2						
BT3855	susD-like							44.5				18.7	29.3	10.5
BT3856	hypothetical protein							24.2						
BT3857	hypothetical protein							25.2						
BT3858	Glycoside hydrolase family 92				12.9			62.6				12.1	13.6	16.6
BT3859	hypothetical protein							38.5						
BT3861	hypothetical protein							46.2						
BT3862	Glycoside hydrolase family 99							50.5				25.6	12.0	10.6
BT3958	susC-like						11.6					12.4		
BT4132	Carbohydrate binding module family 32						100.7							
BT4133	hypothetical protein						395.8							
BT4134	susD-like						303.6					11.1		
BT4135	susC-like						723.8						22.0	
BT4136	Glycoside hydrolase family 29						1136.0					24.7	26.3	17.6
BT4244	Carbohydrate binding module family 32						49.3						187.0	19.3
BT4245	hypothetical protein						36.0						178.8	24.6
BT4246	susD-like						38.9						95.6	11.9
BT4247	susC-like						36.7						86.4	14.3
BT4248	putative anti-sigma factor						34.6						105.7	10.6
BT4249	hypothetical protein						12.9						20.2	
BT4394	Glycoside hydrolase family 20						10.7						14.1	
BT4410	hypothetical protein								40.9		54.1			
BT4411	hypothetical protein								37.3		91.2			
BT4631	putative arylsulfatase precursor						12.4							
BT4632	Carbohydrate binding module family 32						13.9							
BT4633	susD-like						12.4							13.8
BT4634	susC-like						17.1							12.4
BT4652	Polysaccharide lyase family 15									88.9				
BT4653	glucose/galactose transporter									123.1				
BT4654	ROK family transcriptional repressor, with glucokinase domain									51.1				
BT4655	hypothetical protein									93.8				
BT4656	N-acetylglucosamine-6-sulfatase precursor									158.1				
BT4657	Polysaccharide lyase family 12									115.8				
BT4658	Glycoside hydrolase family 88									88.5				
BT4659	susD-like									134.7				
BT4660	susC-like									165.3				
BT4661	hypothetical protein									142.1				
BT4662	Polysaccharide lyase family 12									120.5				
BT4675	Polysaccharide lyase family 13									84.4				
**Down regulated:**														
**Sector 1**														
BT0225	hypothetical protein					24.5						14.0		
BT0504	TonB-dependent outer membrane receptor											20.7		
BT0527	tryptophan synthase alpha chain											28.0	56.0	88.3
BT0528	putative N-(5'-phosphoribosyl)anthranilate isomerase											12.6		
BT0529	indole-3-glycerol phosphate synthase											16.8	23.2	10.9
BT0530	anthranilate phosphoribosyltransferase											11.8		
BT0532	anthranilate synthase component I											12.6		
BT0533	tryptophan synthase beta chain											12.2		
BT0678	cation efflux system protein czcC	25.6	59.5	132.1	157.5	68.1						116.6	204.0	125.3
BT0679	cation efflux system protein czcB	14.8	132.1	78.7	54.1	43.8						29.6	24.0	
BT0680	cation efflux system protein czcA		62.5	100.6	289.0	61.8						57.8	132.5	12.5

BT0988	Mg2+ transport ATPase protein B					30.6	16.0						56.2	96.9	33.2
BT1563	hypothetical protein												14.5	24.8	
BT1566	putative aluminum resistance protein												11.7	17.9	
BT1567	hypothetical protein												10.5	12.6	
BT1574	putative outer membrane protein precursor												24.4		
BT1798	hypothetical protein												14.1	212.4	14.0
BT1895	hypothetical protein												17.2	36.3	85.0
BT1896	putative cell surface antigen												17.5	59.8	20.4
BT2387	O-acetylhomoserine (thiol)-lyase												14.6	14.4	
BT2409	putative TonB-dependent outer membrane protein												37.6	28.3	23.3
BT4037	hypothetical protein										50.8		10.3	16.0	
BT4693	cation efflux system protein					104.9	16.9						52.9	34.8	19.2
BT4694	cation efflux system protein					127.1	17.9						43.3	48.1	33.9
BT4695	outer membrane efflux protein precursor					11.3							21.9	15.7	
**Sector 2**															
BT0226	hypothetical protein					11.9	17.8						12.7		
BT1564	hypothetical protein												15.5	21.5	
**Sector 3**															
BT1573	hypothetical protein				10.3										
BT2157	hypothetical protein					10.2									
**Sector 4**															
BT1419	hypothetical protein		12.0						73.5				23.5	31.4	11.5
**Sector 6**															
BT2156	putative sugar phosphate isomerase/epimerase	11.9		13.7	10.7	15.1				15.6					
BT2158	putative dehydrogenases and related proteins					11.0				10.9					
BT3113	putative transmembrane efflux protein	10.3	12.9						11.9		29.4	16.4		25.9	10.8
BT3240	susC-like	12.9	11.8					11.2				10.1			
BT3241	susD-like	13.2	14.8				13.0	15.1				10.9			
BT3242	hypothetical protein	15.4	17.3				17.4	18.7				18.1			
BT3243	hypothetical protein	10.7	10.7				10.9	11.0				11.9			
BT3244	hypothetical protein	13.5	15.1				14.8	16.4				16.4			
**Sector 7**															
BT1420	hypothetical protein								12.5						
BT2159	putative oxidoreductase									10.3		10.4			
BT3430	ribosomal protein S20										10.2				
BT4499	conserved hypothetical protein, putative membrane protein					18.8			10.6		104.8	16.1			
