# Supplementary material for: Recognition and Degradation of Plant Cell Wall Polysaccharides by Two Human Gut Symbionts
Source: PLoS Biol. 2011 Dec 20;9(12):e1001221. doi: 10.1371/journal.pbio.1001221 (PMC3243724; doi:10.1371/journal.pbio.1001221)
Supplement: Table S2 — Glycan degrading enzymes encoded in B. thetaiotaomicron and B. ovatus PULs. Glycan degrading enzymes are listed as annotated in the CAZy database [14]. Predicted target linkages are based on the known activities within each CAZy family and the linkages known to be present in the substrate presented for growth. (PDF) [file pbio.1001221.s011.pdf]

Table S2. Glycan degrading enzymes encoded in *B. thetaiotaomicron* and *B. ovatus* PULs

PUL	Primary substrate	CAZy family (number in PUL)	Predicted target linkage(s)
BT3674-87	arabinogalactan	GH16 (1)	unknown
		GH43 (2)	β1,3-gal, α-L-ara
		GH105 (1)	α1,2-L-rha-α1,4-galA
BT0262-90	arabinogalactan	GH35 (1)	β-gal
		GH43 (2)	β-gal, α-L-ara
BT0348-69	arabinan	GH43 (3)	α-L-ara[2]
		GH51 (2)	α-L-ara
BT3043-48	arabinan	GH30 (1)	unknown
BT4145-83	rhamnogalacturonan I	GH2 (3)	β-gal
		GH27 (1)	unknown
		GH28 (4)	α1,4-galA; α1,2-L-rha-α1,4-galA
		GH42 (1)	β-gal
		GH105 (2)	L-rha-α1,4-galA
		GH106 (1)	α-L-rha
		PL9 (2)	α1,4-galA
		PL11 (1)	α1,2-L-rha-α1,4-galA
		CE4 (2)	acetyl-GalA
		CE6 (1)	acetyl-GalA
		CE12 (2)	acetyl-GalA
BT0977-30[1]	rhamnogalacturonan II	GH2 (4)	β-gal
		GH28 (1)	galA-α1,4-galA; α1,2-L-rha-α1,4-galA
		GH33 (1)	unknown
		GH43 (1)	β-gal, α-L-ara, β-xyl
		GH78 (3)	α-L-rha
		GH95 (1)	α1,2-L-fuc
		GH105 (1)	L-rha-α1,4-galA
		GH106 (1)	α-L-rha
		PL1 (1)	α1,4-galA
		not classified (13	unknown
BT4667-72[1]	pectic galactan	GH2 (1)	β-gal
		GH53 (1)	β1,4-gal
BT4108-23[1]	homogalacturonan	GH28 (1)	galA-α1,4-galA; α1,2-L-rha-α1,4-galA
		GH105 (1)	L-rha-α1,4-galA
		PL1 (3)	α1,4-galA[3]
		CE8 (2)	methyl-galA
Bacova_02087-97	galacto- and glucomannan	GH26 (2)	β-man
		GH36 (1)	α-gal
Bacova_02640-59	xyloglucan	GH2 (1)	β-gal
		GH3 (2)	β-glu
		GH5 (1)	β-glu
		GH9 (1)	β-glu
		GH31 (1)	α-xyl
		GH43 (2)	β-gal
Bacova_02738-46	β-glucan	GH3 (2)	β-glu
		GH16 (1)	β-glu
Bacova_00942-46	β-glucan	GH30 (1)	β-glu
		GH3 (1)	β-glu
Bacova_03411-50	Wheat arabinoxylan, oat spelt xylan	GH3 (1)	β1,4-xyl
		GH10 (1)	β1,4-xyl
		GH30 (1)	β1,4-xyl (glucuronoxylan-specific)
		GH31 (1)	unknown
		GH43 (5)	β1,4-xyl, α-L-ara
		GH95 (1)	α-L-fuc
		GH97 (1)	unknown
		GH98 (1)	unknown
		GH115 (2)	glcA-α1,2-xyl
		CE1 (1)	acetyl-xyl; feruloyl-ara
		CE6 (2)	acetyl-xyl
Bacova_04384-94	Wheat arabinoxylan, oat spelt xylan	GH10 (2)	β1,4-xyl[4]
		GH43 (1)	β1,4-xyl[4]
		GH67 (1)	glcA-α1,2-xyl
		CE6 (1)	acetyl-xyl

[1]Denotes a *B. thetaiotaomicron* PUL that is conserved in *B. ovatus*

[2]Arabinan specific activity of all three GH43s (BT0360, BT0367 and BT0369) from this PUL was shown experimentally [23].

[3]BT4116 PL1 displays activity vs homogalacturonan (**Fig. S2**)

[4]Xylanase and xylosidase activity has been demonstrated experimentally for one of the GH10s (Bacova_04387) and the GH43 (Bacova_04386), respectively, from this PUL [25].
