# Supplementary material for: Recognition and Degradation of Plant Cell Wall Polysaccharides by Two Human Gut Symbionts
Source: PLoS Biol. 2011 Dec 20;9(12):e1001221. doi: 10.1371/journal.pbio.1001221 (PMC3243724; doi:10.1371/journal.pbio.1001221)
Supplement: Table S3 — Hybrid two-component system mutants analyzed in this study. (PDF) [file pbio.1001221.s012.pdf]

Table S3. Hybrid two-component system mutants analyzed in this study

Disrupted HTCS:	PUL-associated	Substrate defect
BT0267	yes	arabinogalactan
BT0366	yes	arabinan
BT0958	no	none found
BT0981	yes	rhamnogalacturonan II
BT1635	yes	none found
BT1734	no	none found
BT1754	yes	levan, fructose
BT2391	yes	none found
BT2628	yes	none found
BT2860	yes	none found
BT2897	yes	none found
BT2923	yes	none found
BT3049	yes	none found
BT3097	yes	none found
BT3172	yes	none found
BT3302	yes	none found
BT3334	yes	chondroitin sulfate, hyaluronan, dermatan sulfate
BT3465	yes	none found
BT3678	yes	none found
BT3738	no	none found
BT3786	yes	none found
BT4111	yes	homogalacturonan
BT4124	yes	none found
BT4137	yes	none found
BT4178	yes	rhamnogalacturonan I
BT4182	yes	none found
BT4236	no	none found
BT4663	yes	heparin
BT4673	yes	pectic galactan
**Non-disrupted HTCS:**		
BT2826	yes	
BT2971	yes	
BT3134	no	
BT3660	no	
BT3800	no	
BT3951	yes	
BT3957	yes	
