# Supplementary material for: Recognition and Degradation of Plant Cell Wall Polysaccharides by Two Human Gut Symbionts
Source: PLoS Biol. 2011 Dec 20;9(12):e1001221. doi: 10.1371/journal.pbio.1001221 (PMC3243724; doi:10.1371/journal.pbio.1001221)
Supplement: Table S4 — B. ovatus genes with altered expression in vitro during growth on plant glycans or in mice fed a plant glycan rich diet. Mean fold differences in expression compared to growth on minimal medium plus glucose are noted. “Sector” designations for each sub-list refer to the Venn diagram in Figure S6. (PDF) [file pbio.1001221.s013.pdf]

Table S4. *B. ovatus* genes with altered expression *in vitro* during growth on plant glycans or in mice fed a plant
Mean fold differences in expression compared to growth on minimal medium plus glucose
"Sector" designations for each sub-list refer to the Venn diagram in

Gene	Description	β-glucan	Oat spelt xylan	Wheat arabino-xylan	Galacto mannan	Gluco mannan	Xylo glucan	Homogal acturonan	adult plant rich
**Upregulated:**									
**Sector 1 (*in vitro***									
BACOVA_00014	Predicted Protein				16.4				
BACOVA_00032	Hypothetical Protein				10.7				
BACOVA_00034	Hypothetical Protein				10.4				
BACOVA_00035	Predicted Protein				11.8				
BACOVA_00066	Predicted Protein				17.9				
BACOVA_00068	Hypothetical Protein				10.0				
BACOVA_00140	Similar to 4-deoxy-L-threo-5-hexosulose-uronate ketol-isomerase							17.5	
BACOVA_00242	susC-like							12.5	
BACOVA_00321	Hypothetical Protein				16.0				
BACOVA_00370	Similar to IS116 family transposase		15.7						
BACOVA_00858	Similar to mannonate dehydratase							13.0	
BACOVA_00859	Similar to D-mannonate oxidoreductase							13.7	
BACOVA_00860	Conserved Hypothetical Protein							17.3	
BACOVA_00934	susC-like	77.6				17.4			
BACOVA_00936	Predicted Protein	12.1							
BACOVA_00938	Predicted Protein	10.3							
BACOVA_00939	Hypothetical Protein	11.0							
BACOVA_00940	Glycoside hydrolase family 20	10.5							
BACOVA_00942	susC-like	47.6				23.2			
BACOVA_00943	susD-like	29.8				16.3			
BACOVA_00944	Glycoside hydrolase family 30	35.4				21.3			
BACOVA_00945	Hypothetical Protein	32.4				19.4			
BACOVA_00946	Glycoside hydrolase family 3	15.4							
BACOVA_01333	Similar to mannonate dehydratase		135.0					11.2	
BACOVA_01497	Predicted Protein			11.6					
BACOVA_01564	Conserved Hypothetical Protein			33.6					
BACOVA_01565	Conserved Hypothetical Protein			25.1					
BACOVA_01619	Carbohydrate esterase family 7							17.1	
BACOVA_01707	Predicted Protein		39.9	26.6					
BACOVA_01709	Conserved Hypothetical Protein		56.9	41.2					

BACOVA_01711	Similar to L-arabinose isomerase	16.3	12.1				
BACOVA_01712	Similar to L-ribulose-5-phosphate 4-epimerase	20.3	16.1				
BACOVA_01713	Conserved Hypothetical Protein	30.9	24.6				
BACOVA_01715	Similar to aldose 1-epimerase precursor	33.1	20.9				16.8
BACOVA_02061	Glycoside hydrolase family 18				12.4		
BACOVA_02064	susC-like				13.7		
BACOVA_02087	Predicted Protein			184.9	77.9		
BACOVA_02090	Hypothetical Protein			163.2	116.9		
BACOVA_02091	Glycoside hydrolase family 36			169.7	111.8		
BACOVA_02092	Glycoside hydrolase family 26			170.5	101.3		
BACOVA_02093	Glycoside hydrolase family 26			246.9	199.8		
BACOVA_02094	Hypothetical Protein			215.4	127.5		
BACOVA_02095	susD-like			228.8	176.1		
BACOVA_02097	Hypothetical Protein				20.1		
BACOVA_02237	Similar to Na+-transporting NADH:ubiquinone oxidoreductase, Electron transport complex protein rnfC						10.4
BACOVA_02238	Similar to Na+-transporting NADH:ubiquinone oxidoreductase, Electron transport complex protein rnfD						10.6
BACOVA_02239	Hypothetical Protein						11.6
BACOVA_02240	Similar to NADH-ubiquinone oxidoreductase						11.8
BACOVA_02356	Similar to molybdopterin biosynthesis protein						12.1
BACOVA_02532	Conserved Hypothetical Protein	66.7	39.8			23.8	15.7
BACOVA_02533	Similar to xylulose kinase (xylulokinase)	52.9	39.3			33.2	
BACOVA_02558	Similar to UXAC_BACTN Uronate isomerase (Glucuronate isomerase) (Uronic isomerase)						22.0
BACOVA_02559	Similar to transcriptional regulator (LacI family)						28.2
BACOVA_02560	Similar to tagaturonate reductase						24.7
BACOVA_02644	Glycoside hydrolase family 3					185.3	
BACOVA_02645	Glycoside hydrolase family 2					252.4	
BACOVA_02646	Glycoside hydrolase family 31					139.2	
BACOVA_02647	Predicted Protein					22.8	
BACOVA_02648	Hypothetical Protein					19.8	
BACOVA_02649	Glycoside hydrolase family 9					103.8	
BACOVA_02654	Glycoside hydrolase family 43					137.4	
BACOVA_02655	Predicted Protein					118.6	
BACOVA_02656	Glycoside hydrolase family 43					157.2	
BACOVA_02659	Glycoside hydrolase family 3				17.5	100.8	

BACOVA_03088	Polysaccharide lyase family 10					46.7
BACOVA_03170	Predicted Protein			22.0	15.6	
BACOVA_03171	Hypothetical Protein			18.7	14.5	
BACOVA_03172	Conserved Hypothetical Protein			12.3	10.9	
BACOVA_03173	Conserved Hypothetical Protein			12.6		
BACOVA_03175	Hypothetical Protein			10.4		
BACOVA_03333	Glycoside hydrolase family 2			10.3		18.1
BACOVA_03402	susD-like			13.8		
BACOVA_03406	Similar to transcriptional regulator			11.3		12.0
BACOVA_03412	Conserved Hypothetical Protein	17.0				
BACOVA_03413	Conserved Hypothetical Protein	12.9				
BACOVA_03414	Similar to fucose permease	17.3				
BACOVA_03416	Similar to sorbitol dehydrogenase	11.4				
BACOVA_03419	Glycoside hydrolase family 3	24.2	31.2			
BACOVA_03420	Predicted Protein	17.6	26.4			
BACOVA_03438	Glycoside hydrolase family 95		39.3			
BACOVA_03440	Predicted Protein	18.0	57.4			
BACOVA_03443	susC-like	24.1	12.3			
BACOVA_03444	susD-like	13.8				
BACOVA_03445	Predicted Protein	20.0				
BACOVA_03446	Predicted Protein	32.3				
BACOVA_03447	Predicted Protein	17.0				
BACOVA_03448	Predicted Protein		16.4			
BACOVA_03484	Glycoside hydrolase family 97	10.8				
BACOVA_03520	Glycoside hydrolase family 13					50.9
BACOVA_03829	Similar to keto-hydroxyglutarate-aldolase/keto-deoxy-phosphogluconate aldolase					16.7
BACOVA_03830	Similar to 2-dehydro-3-deoxygluconokinase					16.5
BACOVA_03831	Similar to transcriptional regulator					12.8
BACOVA_03832	Similar to altronate hydrolase					25.8
BACOVA_03967	Conserved Hypothetical Protein					12.3
BACOVA_04265	Predicted Protein		18.5			
BACOVA_04266	Conserved Hypothetical Protein		20.8			
BACOVA_04271	Hypothetical Protein		12.8			
BACOVA_04272	Hypothetical Protein		20.8			
BACOVA_04273	Similar to ABC transporter ATP-binding protein		15.1			
BACOVA_04274	Similar to ABC transporter permease		22.2			
BACOVA_04311	Predicted Protein					11.6

BACOVA_04312	Similar to RNA polymerase ECF-type sigma factor					17.3	
BACOVA_04315	Hypothetical Protein					21.2	
BACOVA_04384	Predicted Protein	27.0	15.3				
BACOVA_04896	Similar to hexuronate transporter	12.1				38.8	
BACOVA_04900	Glycoside hydrolase family 105					104.2	
BACOVA_04901	Carbohydrate esterase family 8					108.6	
BACOVA_04902	Carbohydrate esterase family 12-Carbohydrate esterase family 8					53.4	
BACOVA_04903	Predicted Protein					62.2	
BACOVA_04905	Polysaccharide lyase family 1					30.6	
BACOVA_04906	Polysaccharide lyase family 1					381.5	
BACOVA_04907	Polysaccharide lyase family 1					482.9	
BACOVA_04908	Carbohydrate esterase family 8					316.1	
BACOVA_04909	Predicted Protein					164.5	
BACOVA_04910	susD-like					224.1	
BACOVA_04916	Polysaccharide lyase family 1					27.4	
BACOVA_04982	Hypothetical Protein					10.1	
BACOVA_05018	Predicted Protein		11.8				
BACOVA_05037	Glycoside hydrolase family 43					69.3	
BACOVA_05039	Conserved Hypothetical Protein					10.5	
BACOVA_05040	Glycoside hydrolase family 28					251.9	
BACOVA_05176	Hypothetical Protein					14.4	
BACOVA_05203	Hypothetical Protein					13.7	
BACOVA_05541	Hypothetical Protein			14.8			
BACOVA_05601	Similar to NADH-ubiquinone oxidoreductase subunit				21.6		
BACOVA_05603	Similar to NADH:ubiquinone oxidoreductase subunit				14.0		
**Sector 2 (*in vitro, in vivo* shared)**							
BACOVA_00030	Conserved Hypothetical Protein				12.5		176.1
BACOVA_00035	Predicted Protein			14.7			10.0
BACOVA_00557	susD-like	10.1			18.2		19.3
BACOVA_00558	susC-like		12.3		18.1		23.0
BACOVA_00725	Conserved Hypothetical Protein				11.3		15.0
BACOVA_00876	Similar to biotin carboxyl carrier protein				13.4		32.2
BACOVA_00877	Similar to biotin carboxylase	11.3	10.7		20.8		41.4
BACOVA_01282	Similar to tRNA (5-methylaminomethyl-2-thiouridylate)-methyltransferase			42.7		22.7	15.1

BACOVA_01331	Similar to D-mannonate oxidoreductase		127.4					11.9	10.8
BACOVA_01708	Glycoside hydrolase family 51		69.2	47.4				13.4	16.7
BACOVA_01710	Similar to xylulose kinase (xylulokinase)		34.2	27.1					13.8
BACOVA_01714	Similar to Na+/glucose cotransporter		41.1	29.7				13.2	12.1
BACOVA_01889	Hypothetical Protein		18.9	21.7		22.8		35.7	12.6
BACOVA_02052	susD-like		12.9						59.4
BACOVA_02054	Hypothetical Protein		12.1						56.7
BACOVA_02055	Hypothetical Protein	39.5	157.0	10.6		70.6		16.4	822.2
BACOVA_02058	Hypothetical Protein		15.9						88.9
BACOVA_02059	Predicted Protein		27.7			10.9			145.0
BACOVA_02088	Hypothetical Protein				237.3	126.2			12.0
BACOVA_02089	Hypothetical Protein				411.7	248.3			17.7
BACOVA_02096	susC-like				631.9	529.6			15.5
BACOVA_02526	Similar to succinyl-CoA synthetase alpha chain	16.9	24.1	18.6		34.0			28.8
BACOVA_02527	Similar to succinyl-CoA synthetase beta chain	11.3	15.4	13.9		22.8			21.0
BACOVA_02534	Similar to xylose isomerase		42.9	32.8			30.9		10.6
BACOVA_02535	Similar to D-xylose-proton symporter (D-xylose transporter)		65.7	32.7			37.7		11.4
BACOVA_02601	Similar to RNA polymerase ECF-type sigma factor			11.5					16.3
BACOVA_02629	Hypothetical Protein					11.5			81.2
BACOVA_02650	Predicted Protein						303.3		25.9
BACOVA_02651	susD-like						161.5		12.8
BACOVA_02652	susC-like						289.7		18.6
BACOVA_02653	Glycoside hydrolase family 5						183.7		14.9
BACOVA_02741	Glycoside hydrolase family 16	228.8				14.5			149.7
BACOVA_02742	susC-like	259.7				14.8			147.3
BACOVA_02743	susD-like	124.9							70.7
BACOVA_02744	Predicted Protein	298.3				17.9			204.3
BACOVA_02745	Glycoside hydrolase family 3	180.7				12.1			79.9
BACOVA_02746	Hypothetical Protein	232.8				17.8			123.6
BACOVA_02747	Predicted Protein	156.5				13.3			111.9
BACOVA_02771	Similar to cysteine synthase A				21.6			17.5	17.6
BACOVA_03178	Similar to elongation factor G	11.8	22.4	17.1		24.9		22.9	26.7
BACOVA_03415	Similar to fucose permease		16.9						21.6
BACOVA_03417	Glycoside hydrolase family 43		48.2	106.3					24.0
BACOVA_03421	TM-Glycoside hydrolase family 43		71.3	103.4					25.4
BACOVA_03422	Glycoside hydrolase family 31		79.2	150.4					39.0
BACOVA_03423	Glycoside hydrolase family 97		109.5	186.4					52.8

BACOVA_03424	Glycoside hydrolase family 43-Carbohydrate binding module 6	271.8	364.1			98.8
BACOVA_03425	TM-Glycoside hydrolase family 43	163.2	263.9			98.0
BACOVA_03426	susC-like	356.5	417.3			149.9
BACOVA_03427	susD-like	186.9	227.4			83.0
BACOVA_03428	susC-like	107.4	124.8			56.5
BACOVA_03429	susD-like	111.3	138.7			57.8
BACOVA_03430	Predicted Protein	169.9	184.8			96.2
BACOVA_03431	Glycoside hydrolase family 10	84.9	90.1			47.7
BACOVA_03432	Glycoside hydrolase family 5	92.9	124.5			44.8
BACOVA_03433	Glycoside hydrolase family 98-Carbohydrate binding module 35	65.3	95.6			32.0
BACOVA_03434	Glycoside Hydrolase Family 115	89.8	120.1			23.1
BACOVA_03435	Carbohydrate esterase family6-Carbohydrate esterase family 1	52.2	64.3			14.3
BACOVA_03436	Glycoside hydrolase family 43-Carbohydrate binding module 6	95.7	139.5			33.4
BACOVA_03437	Hypothetical Protein	17.4	19.7			15.2
BACOVA_03449	Glycoside Hydrolase Family 115	36.6	81.4			25.4
BACOVA_03450	Carbohydrate esterase family 6-Carbohydrate esterase family 6	36.6	155.1			27.4
BACOVA_03483	Glycoside hydrolase family 51	14.5	10.0			10.6
BACOVA_03514	Glycoside hydrolase family 13	31.9			146.4	34.8
BACOVA_03515	Hypothetical Protein	41.8			176.3	46.5
BACOVA_03516	Hypothetical Protein	14.5			72.0	15.8
BACOVA_03517	susD-like	17.2			90.8	20.3
BACOVA_03518	susC-like	16.4			84.3	20.4
BACOVA_03519	Glycoside hydrolase family 97	15.1			144.9	27.9
BACOVA_03965	Similar to outer membrane efflux protein	28.6				12.4
BACOVA_04103	susC-like			10.3		207.1
BACOVA_04111	Glycoside hydrolase family 92			10.6		66.4
BACOVA_04134	Similar to propionyl-CoA carboxylase beta chain			11.1		28.8
BACOVA_04385	Glycoside hydrolase family 67	413.2	339.7		12.5	47.6
BACOVA_04386	Glycoside hydrolase family 43	158.1	160.9			51.7
BACOVA_04387	Glycoside hydrolase family 10	207.8	184.3			53.7
BACOVA_04388	Hypothetical Protein	189.5	153.8			44.5
BACOVA_04389	Carbohydrate esterase family 6-Carbohydrate esterase family 6	298.4	240.7		10.1	65.8

BACOVA_04390	Glycoside hydrolase family10-Carbohydrate binding module 22-Glycoside hydrolase family 10	1706.9	886.6		13.1	11.5	98.6	305.0
BACOVA_04391	Hypothetical Protein	180.9	82.4					25.4
BACOVA_04392	susD-like	235.3	95.5				14.3	36.5
BACOVA_04393	susC-like	311.6	122.7				13.9	42.8
BACOVA_04500	Similar to glucose/galactose transporter				20.9		60.6	145.9
BACOVA_04501	Glycoside hydrolase family 32						20.6	39.3
BACOVA_04502	Glycoside hydrolase family 91						31.7	81.4
BACOVA_04503	Glycoside hydrolase family 91						17.4	41.6
BACOVA_04504	susD-like						12.0	25.0
BACOVA_04505	susC-like						25.3	44.0
BACOVA_04507	Glycoside hydrolase family 32						34.7	25.5
BACOVA_04622	Predicted Protein	10.8						28.1
BACOVA_04623	Predicted Protein	12.6						26.1
BACOVA_04911	susC-like						283.4	10.2
BACOVA_05038	Predicted Protein						61.3	10.5
BACOVA_05488	Glycoside hydrolase family 53						64.7	81.0
BACOVA_05489	Hypothetical Protein						47.2	62.6
BACOVA_05490	susD-like						47.7	80.5
BACOVA_05491	susC-like						287.8	541.9
BACOVA_05493	Glycoside hydrolase family 5						23.2	43.3
BACOVA_05542	susC-like			14.0				19.0
BACOVA_05543	susD-like			15.7				27.7
BACOVA_05544	Hypothetical Protein			30.6			14.7	75.9
BACOVA_05561	Glycoside hydrolase family 18				10.9			14.3
BACOVA_05563	Hypothetical Protein	10.7			20.7		13.6	30.2
BACOVA_05602	Similar to NADH:ubiquinone oxidoreductase subunit	15.8			36.3		10.1	12.4
**Sector 3 (*in vivo* only)**								
BACOVA_00016	Conserved Hypothetical Protein							30.7
BACOVA_00092	Similar to catalase							11.9
BACOVA_00108	Conserved Hypothetical Protein							12.5
BACOVA_00109	Conserved Hypothetical Protein							11.4
BACOVA_00112	Conserved Hypothetical Protein							13.4
BACOVA_00116	Similar to glutamate/gamma-aminobutyrate anti-porter							17.8
BACOVA_00238	Predicted Protein							13.1
BACOVA_00365	Predicted Protein							11.4
BACOVA_00505	Predicted Protein							10.4

BACOVA_00559	Glycoside hydrolase family 20-Carbohydrate binding module 32	10.1
BACOVA_00560	Glycoside hydrolase family 20-Carbohydrate binding module 32	16.5
BACOVA_00591	Conserved Hypothetical Protein	12.6
BACOVA_00597	Conserved Hypothetical Protein	10.1
BACOVA_00746	Conserved Hypothetical Protein	13.7
BACOVA_00801	Hypothetical Protein	18.2
BACOVA_00802	Hypothetical Protein	40.9
BACOVA_00991	Similar to histidine ammonia-lyase	19.0
BACOVA_00992	Similar to methenyltetrahydrofolate cyclohydrolase	24.0
BACOVA_00993	Similar to imidazolonepropionase	14.1
BACOVA_00994	Similar to formiminotransferase-cyclodeaminase	18.6
BACOVA_00995	Similar to urocanate hydratase	19.0
BACOVA_01069	Similar to aspartate ammonia-lyase	12.1
BACOVA_01071	Similar to L-asparaginase II precursor	16.1
BACOVA_01072	Conserved Hypothetical Protein	26.8
BACOVA_01270	Conserved Hypothetical Protein	17.8
BACOVA_01290	Conserved Hypothetical Protein	15.7
BACOVA_01329	Similar to oxidoreductase, aldo/keto reductase	12.3
BACOVA_01355	Conserved Hypothetical Protein	92.8
BACOVA_01415	Predicted Protein	31.0
BACOVA_01467	Hypothetical Protein	13.5
BACOVA_01476	Conserved Hypothetical Protein	12.4
BACOVA_01482	Hypothetical Protein	10.7
BACOVA_01654	Similar to flavodoxin	10.2
BACOVA_01792	Hypothetical Protein	65.8
BACOVA_01793	Conserved Hypothetical Protein	113.3
BACOVA_01794	Similar to arabinose-proton symporter	52.7
BACOVA_01795	Similar to N-acylglucosamine 2-epimerase	99.2
BACOVA_01796	susC-like	99.8
BACOVA_01797	susD-like	105.7
BACOVA_01798	Conserved Hypothetical Protein	105.0
BACOVA_01799	Hypothetical Protein	53.6
BACOVA_01800	Conserved Hypothetical Protein	85.7
BACOVA_01801	Conserved Hypothetical Protein	49.4
BACOVA_01802	Carbohydrate esterase family6	45.7
BACOVA_01803	Conserved Hypothetical Protein	83.5

BACOVA_01807	Hypothetical Protein	26.5
BACOVA_01808	Carbohydrate esterase family 6	35.5
BACOVA_01809	Hypothetical Protein	73.1
BACOVA_01810	Predicted Protein	27.4
BACOVA_01811	Predicted Protein	48.4
BACOVA_01812	Predicted Protein	64.8
BACOVA_01813	Hypothetical Protein	113.7
BACOVA_01814	susD-like	37.7
BACOVA_01815	susC-like	87.2
BACOVA_01816	Similar to N-acylglucosamine 2-epimerase	77.4
BACOVA_01818	Similar to arabinose-proton symporter	65.4
BACOVA_01819	Conserved Hypothetical Protein	62.5
BACOVA_01893	Similar to pyrophosphate-energized vacuolar membrane proton pump	10.6
BACOVA_01895	Hypothetical Protein	88.1
BACOVA_01896	Glycoside hydrolase family 36	17.0
BACOVA_02051	susC-like	34.1
BACOVA_02053	Hypothetical Protein	10.6
BACOVA_02056	Carbohydrate esterase family 1	34.3
BACOVA_02057	Carbohydrate esterase family 1	49.8
BACOVA_02168	Hypothetical Protein	10.3
BACOVA_02174	susD-like	17.1
BACOVA_02175	susC-like	13.0
BACOVA_02205	Similar to RNA polymerase ECF-type sigma factor	26.5
BACOVA_02364	Hypothetical Protein	27.3
BACOVA_02442	Conserved Hypothetical Protein	10.9
BACOVA_02495	Hypothetical Protein	16.3
BACOVA_02504	Hypothetical Protein	17.1
BACOVA_02539	Conserved Hypothetical Protein	18.2
BACOVA_02602	Similar to RNA polymerase ECF-type sigma factor	17.5
BACOVA_02626	Glycoside hydrolase family 5	36.2
BACOVA_02627	susC-like	64.3
BACOVA_02628	susD-like	34.0
BACOVA_02630	Glycoside hydrolase family 5	23.1
BACOVA_02631	Glycoside hydrolase family 2	14.5
BACOVA_02632	Glycoside hydrolase family 5	19.3
BACOVA_02634	Hypothetical Protein	11.5
BACOVA_02635	Hypothetical Protein	10.6
BACOVA_02636	Glycoside hydrolase family 78	10.0

BACOVA_02663	Hypothetical Protein	19.5
BACOVA_02664	susD-like	11.3
BACOVA_02665	susC-like	12.3
BACOVA_02690	Similar to pyruvate carboxylase subunit B)	15.1
BACOVA_02976	Predicted Protein	15.8
BACOVA_03020	Similar to L-asparaginase I	10.1
BACOVA_03027	Predicted Protein	14.4
BACOVA_03089	Conserved Hypothetical Protein	11.1
BACOVA_03090	Similar to multidrug efflux membrane fusion protein	11.8
BACOVA_03130	Hypothetical Protein	17.1
BACOVA_03131	Hypothetical Protein	31.2
BACOVA_03153	susD-like	80.5
BACOVA_03154	susC-like	28.8
BACOVA_03190	Conserved Hypothetical Protein	10.7
BACOVA_03262	Hypothetical Protein	43.3
BACOVA_03263	Hypothetical Protein	97.7
BACOVA_03264	Hypothetical Protein	116.2
BACOVA_03265	Hypothetical Protein	16.4
BACOVA_03294	Similar to choloylglycine hydrolase	10.8
BACOVA_03335	Predicted Protein	15.0
BACOVA_03379	Conserved Hypothetical Protein	14.4
BACOVA_03380	Glycoside hydrolase family 3	16.6
BACOVA_03381	susD-like	19.3
BACOVA_03382	susC-like	18.4
BACOVA_03485	Hypothetical Protein	11.6
BACOVA_03556	susC-like	22.2
BACOVA_03557	susD-like	26.1
BACOVA_03558	Glycoside hydrolase family 18	21.2
BACOVA_03559	Hypothetical Protein	22.2
BACOVA_03579	susD-like	17.0
BACOVA_03580	Hypothetical Protein	10.8
BACOVA_03601	Similar to rhamnulose kinase/L-fuculose kinase	13.7
BACOVA_03602	Similar to L-rhamnose isomerase	33.0
BACOVA_03603	Similar to L-rhamnose/H+ symporter	22.1
BACOVA_03625	Predicted Protein	12.4
BACOVA_03641	Predicted Protein	11.1
BACOVA_03667	Conserved Hypothetical Protein	27.5
BACOVA_03761	Hypothetical Protein	20.6
BACOVA_03769	Similar to polyphosphate kinase	12.0

BACOVA_03770	Conserved Hypothetical Protein	11.0
BACOVA_03810	Glycoside hydrolase family 20	10.8
BACOVA_03843	Conserved Hypothetical Protein	10.0
BACOVA_03864	Hypothetical Protein	16.9
BACOVA_04005	Hypothetical Protein	20.9
BACOVA_04094	Predicted Protein	44.6
BACOVA_04095	Hypothetical Protein	35.5
BACOVA_04096	Hypothetical Protein	21.6
BACOVA_04097	Glycoside hydrolase family 18	29.9
BACOVA_04098	susD-like	17.1
BACOVA_04099	susC-like	26.1
BACOVA_04104	susD-like	147.8
BACOVA_04105	Glycoside hydrolase family 18	131.0
BACOVA_04106	Hypothetical Protein	130.7
BACOVA_04107	Hypothetical Protein	113.8
BACOVA_04108	Carbohydrate binding module 32	29.5
BACOVA_04109	Conserved Hypothetical Protein	31.0
BACOVA_04110	Conserved Hypothetical Protein	55.6
BACOVA_04131	Hypothetical Protein	39.3
BACOVA_04132	Hypothetical Protein	29.4
BACOVA_04136	Conserved Hypothetical Protein	10.3
BACOVA_04138	Similar to L-lactate permease	11.1
BACOVA_04222	Similar to tyrosine phenol-lyase	12.2
BACOVA_04235	Predicted Protein	15.9
BACOVA_04297	Similar to alanine dehydrogenase	10.3
BACOVA_04314	Conserved Hypothetical Protein	17.3
BACOVA_04408	Hypothetical Protein	12.7
BACOVA_04497	Predicted Protein	14.7
BACOVA_04565	Similar to pyruvate dehydrogenase	11.7
BACOVA_04684	Conserved Hypothetical Protein	32.8
BACOVA_04700	Hypothetical Protein	10.0
BACOVA_04701	Hypothetical Protein	26.2
BACOVA_04702	Hypothetical Protein	56.3
BACOVA_04703	Similar to Fe3+ ABC transporter, permease protein	24.2
BACOVA_04704	Similar to Fe3+ ABC transporter, periplasmic iron-binding protein	27.7
BACOVA_04705	Hypothetical Protein	19.7
BACOVA_04706	Hypothetical Protein	27.3
BACOVA_04707	Hypothetical Protein	45.1
BACOVA_04708	Hypothetical Protein	43.6

BACOVA_04735	Predicted Protein				19.7
BACOVA_04786	Hypothetical Protein				15.2
BACOVA_04799	Predicted Protein				298.5
BACOVA_04800	Glycoside hydrolase family 18				254.1
BACOVA_04801	Hypothetical Protein				189.1
BACOVA_04802	Glycoside hydrolase family 18				108.3
BACOVA_04803	susD-like				134.2
BACOVA_04804	susC-like				73.6
BACOVA_04805	Hypothetical Protein				31.5
BACOVA_04806	Hypothetical Protein				19.6
BACOVA_04807	Glycoside hydrolase family 18				25.5
BACOVA_04808	Hypothetical Protein				17.1
BACOVA_04809	Glycoside hydrolase family 92				13.1
BACOVA_04810	Glycoside hydrolase family 92				10.3
BACOVA_04811	Hypothetical Protein				12.2
BACOVA_04851	Predicted Protein				15.4
BACOVA_04960	Predicted Protein				15.3
BACOVA_04974	Polysaccharide lyase family 11				27.1
BACOVA_04975	Glycoside hydrolase family 105				16.7
BACOVA_04976	Conserved Hypothetical Protein				17.7
BACOVA_05006	Hypothetical Protein				13.6
BACOVA_05024	Predicted Protein				38.2
BACOVA_05031	Glycoside hydrolase family 105				12.4
BACOVA_05125	Predicted Protein				10.6
BACOVA_05295	Conserved Hypothetical Protein				11.9
BACOVA_05305	Conserved Hypothetical Protein				11.5
BACOVA_05351	Predicted Protein				25.1
BACOVA_05401	Conserved Hypothetical Protein				31.0
BACOVA_05433	Hypothetical Protein				31.2
BACOVA_05503	Predicted Protein				10.0
BACOVA_05559	susC-like				12.2
BACOVA_05560	susD-like				12.2
BACOVA_05564	Hypothetical Protein				10.5
BACOVA_05569	Hypothetical Protein				11.9

Downregulated:

Sector 1

BACOVA_01128	Conserved Hypothetical Protein			10.8	
BACOVA_01198	susC	36.6	17.0	36.0	
BACOVA_01199	susD	12.5			

BACOVA_02195	Similar to transcriptional regulator		11.6				
BACOVA_02732	Hypothetical Protein	13.2					
BACOVA_02733	Hypothetical Protein	17.4					
BACOVA_02734	Predicted Protein	12.3					
BACOVA_02735	Hypothetical Protein	12.7					
BACOVA_02814	Conserved Hypothetical Protein	10.0	18.6		18.4		
BACOVA_02953	Predicted Protein			16.6		10.8	
BACOVA_02953	Predicted Protein			15.7			
BACOVA_03637	Predicted Protein					23.1	
BACOVA_03722	Similar to small heat shock protein					12.4	
BACOVA_03757	Similar to glutamate synthase, small subunit	23.7					
BACOVA_03765	Similar to nitrogen regulatory protein P-II	10.2					
BACOVA_03767	Similar to glutamine synthetase		10.6				
BACOVA_04836	Predicted Protein		11.3				
**Sector 2**							
BACOVA_02729	susC	12.5	14.8				12.8
BACOVA_02028	Predicted Protein	11.9	19.2				10.9
BACOVA_02730	susD	10.0					16.2
BACOVA_02382	Similar to cation efflux system protein czcB		390.6				55.2
BACOVA_02381	Similar to cation efflux system protein czcC		123.3				39.3
BACOVA_02383	Similar to cation efflux system protein czcA		73.5				50.8
BACOVA_00178	Similar to Mg2+ transport ATPase protein B		20.5				553.6
BACOVA_02194	Hypothetical Protein		14.8				12.5
BACOVA_04142	Hypothetical Protein		14.0				27.6
BACOVA_04144	Similar to protein rtcB		12.0				18.1
BACOVA_04143	Hypothetical Protein		10.4				32.9
BACOVA_03166	Conserved Hypothetical Protein				22.2		17.8
BACOVA_03813	Similar to TonB-dependent outer membrane receptor				10.2		19.1
**Sector 3**							
BACOVA_04528	Hypothetical Protein						299.4
BACOVA_01521	Conserved Hypothetical Protein						93.5
BACOVA_03780	Similar to anthranilate synthase component I						72.6
BACOVA_04530	Conserved Hypothetical Protein						47.6
BACOVA_03015	Predicted Protein						45.5
BACOVA_03016	Hypothetical Protein						42.0
BACOVA_00832	Hypothetical Protein						39.6
BACOVA_00833	Conserved Hypothetical Protein						35.4
BACOVA_03015	Predicted Protein						33.2

BACOVA_03815	Similar to TonB-dependent outer membrane receptor	30.9
BACOVA_04529	Hypothetical Protein	28.9
BACOVA_04526	Hypothetical Protein	28.6
BACOVA_01522	Conserved Hypothetical Protein	27.6
BACOVA_00095	Predicted Protein	27.6
BACOVA_04262	Conserved Hypothetical Protein	23.3
BACOVA_01520	Hypothetical Protein	23.1
BACOVA_03782	Similar to anthranilate phosphoribosyltransferase	21.5
BACOVA_03781	Similar to anthranilate synthase component II	14.7
BACOVA_03014	Predicted Protein	13.5
BACOVA_00270	Hypothetical Protein	13.4
BACOVA_01519	Predicted Protein	13.3
BACOVA_04368	HTCS	12.8
BACOVA_04307	Conserved Hypothetical Protein	12.2
BACOVA_01233	Similar to histidinol-phosphate aminotransferase	12.2
BACOVA_04307	Conserved Hypothetical Protein	12.0
BACOVA_02394	Conserved Hypothetical Protein	11.9
BACOVA_02395	Hypothetical Protein	11.6
BACOVA_03731	Similar to polysaccharide export outer membrane protein	11.3
BACOVA_04306	Conserved Hypothetical Protein	11.3
BACOVA_03789	Hypothetical Protein	10.2
BACOVA_05110	Similar to NADPH-dependent glutamate synthase small chain	10.0
BACOVA_05596	Similar to pyruvate formate-lyase activating enzyme	10.0
