# Supplementary material for: Recognition and Degradation of Plant Cell Wall Polysaccharides by Two Human Gut Symbionts
Source: PLoS Biol. 2011 Dec 20;9(12):e1001221. doi: 10.1371/journal.pbio.1001221 (PMC3243724; doi:10.1371/journal.pbio.1001221)
Supplement: Table S5 — Putative PULs identified in the B. ovatus ATCC8384 genome. B. ovatus PULs that contain genes expressed in vivo in the ceca of mice fed a plant rich diet are indicated in the final column. Abbreviations and notes: extracytoplasmic function sigma factor, ECF; hybrid two-component system, HTCS; regulators with homology to other families of PUL-associated regulators are indicated (SusR, GntR, AraC); the presence of a potential regulator in a different family is indicated as “unknown.” (PDF) [file pbio.1001221.s014.pdf]

Table S5. Putative PULs identified in the *B. ovatus* ATCC8384 genome

Abbreviations and notes: extracytoplasmic function sigma factor, ECF; hybrid two-component system, HTCS.
Regulators with homology to other families of PUL-associated regulators are indicated: SusR, GntR, AraC.
The presence of a potential regulator in a different family is indicated as "unknown".

B. ovatus PULs that contain genes expressed *in vivo* in the ceca of mce fed a plant rich diet are indicated in the final column.

PUL number	Regulator	Gene range	CAZy annotation	*In vivo* expressed PULs (induced genes are indicated and highlighted in yellow)
1	ECF	BACOVA_00098		
		BACOVA_00101	susC-like	
		BACOVA_00102	susD-like	
		BACOVA_00103		
		BACOVA_00104	Glycoside Hydrolase Family 20	
2	none	BACOVA_00136		
		BACOVA_00137	susD-like	
		BACOVA_00138	susC-like	
3	ECF	BACOVA_00154		
		BACOVA_00155		
		BACOVA_00156	susC-like	
		BACOVA_00157	susD-like	
		BACOVA_00158		
		BACOVA_00159	Glycoside Hydrolase Family 18	
4	HTCS	BACOVA_00239		
		BACOVA_00240		
		BACOVA_00241		
		BACOVA_00242	susC-like	
		BACOVA_00243	susD-like	
		BACOVA_00244	susC-like	
		BACOVA_00245	susD-like	
		BACOVA_00246		
		BACOVA_00247	Glycoside Hydrolase Family 10	
		BACOVA_00248	Polysaccharide Lyase Family 9	
		BACOVA_00249	Glycoside Hydrolase Family 115	

		BACOVA_00250	Glycoside Hydrolase Family 2
5	none	BACOVA_00264	
		BACOVA_00265	susC-like
		BACOVA_00266	susD-like
		BACOVA_00267	
6	ECF	BACOVA_00272	
		BACOVA_00273	
		BACOVA_00274	
		BACOVA_00275	
		BACOVA_00276	
		BACOVA_00277	
		BACOVA_00278	
		BACOVA_00279	
		BACOVA_00280	susD-like
		BACOVA_00281	susC-like
		BACOVA_00282	
		BACOVA_00283	
		BACOVA_00284	
7	none	BACOVA_00298	
		BACOVA_00299	
		BACOVA_00300	
		BACOVA_00301	
		BACOVA_00302	
		BACOVA_00303	
		BACOVA_00304	
		BACOVA_00305	
		BACOVA_00306	
		BACOVA_00307	
		BACOVA_00308	
		BACOVA_00309	susD-like
		BACOVA_00310	susC-like
8	ECF	BACOVA_00425	
		BACOVA_00426	Glycoside Hydrolase Family 3
		BACOVA_00427	Glycoside Hydrolase Family 3
		BACOVA_00428	Glycoside Hydrolase Family 3
		BACOVA_00429	

		BACOVA_00430		
		BACOVA_00431		
		BACOVA_00432		
		BACOVA_00433	susD-like	
		BACOVA_00434	susC-like	
		BACOVA_00435		
		BACOVA_00436		
9	none	BACOVA_00556		
		BACOVA_00557	susD-like	bacova_00557-60 are induced *in vivo* in plant fed mice
		BACOVA_00558	susC-like	
		BACOVA_00559	Glycoside Hydrolase Family 20-Carbohydrate Binding Module 32	
		BACOVA_00560	Glycoside Hydrolase Family 20-Carbohydrate Binding Module 32	
		BACOVA_00561		
		BACOVA_00562	Glycoside Hydrolase Family 2	
		BACOVA_00563	Glycoside Hydrolase Family 33	
10	ECF	BACOVA_00619		
		BACOVA_00620		
		BACOVA_00621	susC-like	
		BACOVA_00622	susD-like	
		BACOVA_00623	susC-like	
		BACOVA_00624	susD-like	
		BACOVA_00625	Glycoside Hydrolase Family 16-Glycoside Hydrolase Family 3	
		BACOVA_00626		
		BACOVA_00627		
11	none	BACOVA_00653		
		BACOVA_00654		
		BACOVA_00655		
		BACOVA_00656		
		BACOVA_00657	susC-like	
		BACOVA_00658	susD-like	
		BACOVA_00659		
		BACOVA_00660		

		BACOVA_00661	
12		BACOVA_00681	susD-like
		BACOVA_00682	susC-like
13	ECF	BACOVA_00832	
		BACOVA_00833	
		BACOVA_00834	
		BACOVA_00835	
		BACOVA_00836	
		BACOVA_00837	Glycoside Hydrolase Family 2
		BACOVA_00838	
		BACOVA_00839	susD-like
		BACOVA_00840	susC-like
		BACOVA_00841	
		BACOVA_00842	
14	none	BACOVA_00865	susD-like
		BACOVA_00866	susC-like
15	ECF	BACOVA_00883	
		BACOVA_00884	
		BACOVA_00885	susC-like
		BACOVA_00886	susD-like
		BACOVA_00887	
		BACOVA_00888	
		BACOVA_00889	
		BACOVA_00890	
		BACOVA_00891	
		BACOVA_00892	
16	ECF	BACOVA_00915	
		BACOVA_00916	
		BACOVA_00917	
		BACOVA_00918	susD-like
		BACOVA_00919	susC-like
		BACOVA_00920	
		BACOVA_00921	
		BACOVA_00922	
		BACOVA_00923	
17	SusR	BACOVA_00932	

		BACOVA_00933	
		BACOVA_00934	susC-like
		BACOVA_00935	susD-like
		BACOVA_00936	
		BACOVA_00937	
		BACOVA_00938	
		BACOVA_00939	
		BACOVA_00940	Glycoside Hydrolase Family 20
18	SusR	BACOVA_00941	
		BACOVA_00942	susC-like
		BACOVA_00943	susD-like
		BACOVA_00944	Glycoside Hydrolase Family 30
		BACOVA_00945	
		BACOVA_00946	Glycoside Hydrolase Family 3
19	HTCS	BACOVA_00974	
		BACOVA_00975	
		BACOVA_00976	
		BACOVA_00977	Glycoside Hydrolase Family 5-Carbohydrate Binding Module 35
		BACOVA_00978	susC-like
		BACOVA_00979	susD-like
		BACOVA_00980	Carbohydrate Esterase 12-Carbohydrate Esterase 4
		BACOVA_00981	Glycoside Hydrolase Family 43
		BACOVA_00982	Glycoside Hydrolase Family 115
20	ECF	BACOVA_001132	
		BACOVA_001133	
		BACOVA_001134	
		BACOVA_001135	
		BACOVA_001136	
		BACOVA_001137	
		BACOVA_001138	
		BACOVA_001139	
21	none	BACOVA_001198	
		BACOVA_001199	
22	HTCS	BACOVA_01213	

		BACOVA_01214	Glycoside Hydrolase Family 31
		BACOVA_01215	Glycoside Hydrolase Family 76
		BACOVA_01216	Carbohydrate Binding Module 32
		BACOVA_01217	susC-like
		BACOVA_01218	susD-like
		BACOVA_01219	
		BACOVA_01220	
		BACOVA_01221	Glycoside Hydrolase Family 31
		BACOVA_01222	Glycoside Hydrolase Family 92
		BACOVA_01223	Glycoside Hydrolase Family 92
23	none	BACOVA_01224	
		BACOVA_01225	
		BACOVA_01226	
		BACOVA_01227	susD-like
		BACOVA_01228	susC-like
24	ECF	BACOVA_01243	
		BACOVA_01244	
		BACOVA_01245	
		BACOVA_01246	
		BACOVA_01247	susD-like
		BACOVA_01248	susC-like
		BACOVA_01249	
		BACOVA_01250	
		BACOVA_01251	
25	HTCS	BACOVA_01291	
		BACOVA_01292	Glycoside Hydrolase Family 88
		BACOVA_01293	Glycoside Hydrolase Family 43
		BACOVA_01294	
		BACOVA_01295	
		BACOVA_01296	susD-like
		BACOVA_01297	susC-like
		BACOVA_01298	
		BACOVA_01299	
		BACOVA_01300	
		BACOVA_01301	
26	HTCS	BACOVA_01304	Glycoside Hydrolase Family 2

		BACOVA_01305	
		BACOVA_01306	
		BACOVA_01307	susD-like
		BACOVA_01308	susC-like
		BACOVA_01309	
		BACOVA_01310	
		BACOVA_01311	
27	HTCS	BACOVA_01312	Polysaccharide Lyase Family 15
		BACOVA_01313	
		BACOVA_01314	
		BACOVA_01315	Glycoside Hydrolase Family 28
		BACOVA_01316	
		BACOVA_01317	Glycoside Hydrolase Family 30
		BACOVA_01318	
		BACOVA_01319	
		BACOVA_01320	susD-like
		BACOVA_01321	susC-like
		BACOVA_01322	
		BACOVA_01323	
		BACOVA_01324	
28	none	BACOVA_01382	
		BACOVA_01383	
		BACOVA_01384	susD-like
		BACOVA_01386	susC-like
29	none	BACOVA_01405	susC-like
		BACOVA_01406	susD-like
30	ECF	BACOVA_01604	
		BACOVA_01605	
		BACOVA_01606	susC-like
		BACOVA_01607	susD-like
		BACOVA_01608	
		BACOVA_01609	Glycoside Hydrolase Family 27
		BACOVA_01610	Carbohydrate Binding Module 35
		BACOVA_01611	
31	HTCS	BACOVA_01661	Glycoside Hydrolase Family 109
		BACOVA_01662	

		BACOVA_01663		
		BACOVA_01664		
		BACOVA_01665		
		BACOVA_01666	susD-like	
		BACOVA_01667	susC-like	
		BACOVA_01668		
		BACOVA_01669		
		BACOVA_01670		
		BACOVA_01671	Glycoside Hydrolase Family 2	
		BACOVA_01672	Glycoside Hydrolase Family 88	
		BACOVA_01673		
		BACOVA_01674		
		BACOVA_01675		
32	ECF	BACOVA_01682		
		BACOVA_01683		
		BACOVA_01684	susC-like	
		BACOVA_01685	susD-like	
		BACOVA_01686		
		BACOVA_01687	Glycoside Hydrolase Family 88- Glycoside Hydrolase Family 92	
33	none	BACOVA_01792		bacova_01792-1803 are induced *in vivo* in plant fed mice
		BACOVA_01793		
		BACOVA_01794		
		BACOVA_01795		
		BACOVA_01796	susC-like	
		BACOVA_01797	susD-like	
		BACOVA_01798		
		BACOVA_01799		
		BACOVA_01800		
		BACOVA_01801		
		BACOVA_01802	Carbohydrate Esterase 6	
		BACOVA_01803		
34	none	BACOVA_01804		
		BACOVA_01805		
		BACOVA_01806		

	BACOVA_01807		bacova_01807-19 are induced *in vivo* in plant fed mice
	BACOVA_01808	Carbohydrate Esterase 6	
	BACOVA_01809		
	BACOVA_01810		
	BACOVA_01811		
	BACOVA_01812		
	BACOVA_01813		
	BACOVA_01814	susD-like	
	BACOVA_01815	susC-like	
	BACOVA_01816		
	BACOVA_01817		
	BACOVA_01818		
	BACOVA_01819		
35 HTCS	BACOVA_01822		
	BACOVA_01823		
	BACOVA_01824		
	BACOVA_01825	susC-like	
	BACOVA_01826	susD-like	
	BACOVA_01827		
	BACOVA_01828		
	BACOVA_01829		
	BACOVA_01830		
	BACOVA_01831		
	BACOVA_01832	susC-like	
	BACOVA_01833	susD-like	
	BACOVA_01834	susC-like	
	BACOVA_01835	susD-like	
	BACOVA_01836		
	BACOVA_01837		
	BACOVA_01838		
	BACOVA_01839		
	BACOVA_01840		
	BACOVA_01841		
	BACOVA_01842		
	BACOVA_01843		

	BACOVA_01844		
36 HTCS	BACOVA_01967	Polysaccharide Lyase Family 8	
	BACOVA_01968		
	BACOVA_01969	Glycoside Hydrolase Family 88	
	BACOVA_01996		
	BACOVA_01997		
	BACOVA_01998	susC-like	
	BACOVA_01999	susD-like	
	BACOVA_02000		
	BACOVA_02001		
	BACOVA_02002		
37 none	BACOVA_01971	Glycoside Hydrolase Family 2	
	BACOVA_01972		
	BACOVA_01973	Glycoside Hydrolase Family 43	
	BACOVA_01974	susD-like	
	BACOVA_01975	susC-like	
	BACOVA_01976		
38 none	BACOVA_01986		
	BACOVA_01987	susC-like	
	BACOVA_01988	susD-like	
	BACOVA_01989	susC-like	
39 ECF	BACOVA_02036		
	BACOVA_02037		
	BACOVA_02038	Glycoside Hydrolase Family 89	
	BACOVA_02039		
	BACOVA_02040		
	BACOVA_02041		
	BACOVA_02042	Glycoside Hydrolase Family 89	
	BACOVA_02043		
	BACOVA_02044	susD-like	
	BACOVA_02045	susC-like	
	BACOVA_02046		
40 LacI	BACOVA_02049		
	BACOVA_02050		
	BACOVA_02051	susC-like	bacova_02051-59 are induced *in vivo* in plant fed mice

	BACOVA_02052	susD-like
	BACOVA_02053	
	BACOVA_02054	
	BACOVA_02055	
	BACOVA_02056	Carbohydrate Esterase 1
	BACOVA_02057	Carbohydrate Esterase 1
	BACOVA_02058	
	BACOVA_02059	
41 HTCS	BACOVA_02060	
	BACOVA_02061	Glycoside Hydrolase Family 18
	BACOVA_02062	
	BACOVA_02063	susD-like
	BACOVA_02064	susC-like
	BACOVA_02065	
42 HTCS	BACOVA_02087	
	BACOVA_02088	
	BACOVA_02089	
	BACOVA_02090	
	BACOVA_02091	Glycoside Hydrolase Family 36
	BACOVA_02092	Glycoside Hydrolase Family 26
	BACOVA_02093	Glycoside Hydrolase Family 26
	BACOVA_02094	
	BACOVA_02095	susD-like
	BACOVA_02096	susC-like
	BACOVA_02097	
43 ECF	BACOVA_02102	
	BACOVA_02103	
	BACOVA_02104	Glycoside Hydrolase Family 2
	BACOVA_02105	Carbohydrate Esterase 7
	BACOVA_02106	Glycoside Hydrolase Family 36
	BACOVA_02107	
	BACOVA_02108	Glycoside Hydrolase Family 105
	BACOVA_02109	Glycoside Hydrolase Family 3
	BACOVA_02110	Glycoside Hydrolase Family 3
	BACOVA_02111	
	BACOVA_02112	

	BACOVA_02113		
	BACOVA_02114		
	BACOVA_02115	susC-like	
	BACOVA_02116	susD-like	
	BACOVA_02117		
44 HTCS	BACOVA_02156		
	BACOVA_02157		
	BACOVA_02158	Glycoside Hydrolase Family 105	
	BACOVA_02159	Glycoside Hydrolase Family 105	
	BACOVA_02160	Glycoside Hydrolase Family 2	
	BACOVA_02161		
	BACOVA_02162	Glycoside Hydrolase Family 2	
	BACOVA_02163		
	BACOVA_02164		
	BACOVA_02165		
	BACOVA_02166	susD-like	
	BACOVA_02167	susC-like	
	BACOVA_02168		
	BACOVA_02169	Glycoside Hydrolase Family 43	
	BACOVA_02170		
	BACOVA_02171	Glycoside Hydrolase Family 105	
	BACOVA_02172		
	BACOVA_02173	Glycoside Hydrolase Family 115	
	BACOVA_02174	susD-like	bacova_02174-75 are induced *in vivo* in plant fed mice
	BACOVA_02175	susC-like	
	BACOVA_02176		
	BACOVA_02177		
	BACOVA_02178	Glycoside Hydrolase Family 109	
	BACOVA_02179		
	BACOVA_02180	Glycoside Hydrolase Family 109	
	BACOVA_02181	Glycoside Hydrolase Family 43	
	BACOVA_02182		
	BACOVA_02183		
45 ECF	BACOVA_02488		
	BACOVA_02489		

		BACOVA_02490	susC-like	
		BACOVA_02491	susD-like	
		BACOVA_02492		
		BACOVA_02493	Glycoside Hydrolase Family 2	
46	none	BACOVA_02626	Glycoside Hydrolase Family 5	bacova_02626-36 are induced *in vivo* in plant fed mice
		BACOVA_02627	susC-like	
		BACOVA_02628	susD-like	
		BACOVA_02629		
		BACOVA_02630	Glycoside Hydrolase Family 5	
		BACOVA_02631	Glycoside Hydrolase Family 2	
		BACOVA_02632	Glycoside Hydrolase Family 5	
		BACOVA_02633		
		BACOVA_02634		
		BACOVA_02635		
		BACOVA_02636	Glycoside Hydrolase Family 78	
		BACOVA_02637		
		BACOVA_02638	Carbohydrate Esterase 6-Carbohydrate Esterase 6	
		BACOVA_02639		
47	HTCS	BACOVA_02640		
		BACOVA_02641		
		BACOVA_02642		
		BACOVA_02643		
		BACOVA_02644	Glycoside Hydrolase Family 3	
		BACOVA_02645	Glycoside Hydrolase Family 2	
		BACOVA_02646	Glycoside Hydrolase Family 31	
		BACOVA_02647		
		BACOVA_02648		
		BACOVA_02649	Glycoside Hydrolase Family 9	
		BACOVA_02650		
		BACOVA_02651	susD-like	
		BACOVA_02652	susC-like	
		BACOVA_02653	Glycoside Hydrolase Family 5	
		BACOVA_02654	Glycoside Hydrolase Family 43	
		BACOVA_02655		

	BACOVA_02656	Glycoside Hydrolase Family 43
	BACOVA_02657	
	BACOVA_02658	
	BACOVA_02659	Glycoside Hydrolase Family 3
48 HTCS	BACOVA_02661	Glycoside Hydrolase Family 5
	BACOVA_02662	Glycoside Hydrolase Family 43
	BACOVA_02663	
	BACOVA_02664	susD-like
	BACOVA_02665	susC-like
	BACOVA_02666	
	BACOVA_02667	
49 HTCS	BACOVA_02692	Glycoside Hydrolase Family 2
	BACOVA_02693	Carbohydrate Esterase 12
	BACOVA_02694	
	BACOVA_02695	
	BACOVA_02696	
	BACOVA_02697	
	BACOVA_02698	
	BACOVA_02699	Glycoside Hydrolase Family 106
	BACOVA_02700	
	BACOVA_02701	
	BACOVA_02702	Polysaccharide Lyase Family 11
	BACOVA_02703	susD-like
	BACOVA_02704	susC-like
	BACOVA_02705	
	BACOVA_02706	
	BACOVA_02707	susD-like
	BACOVA_02708	susC-like
	BACOVA_02709	
	BACOVA_02710	Glycoside Hydrolase Family 2
	BACOVA_02711	Glycoside Hydrolase Family 88
	BACOVA_02712	
	BACOVA_02713	
	BACOVA_02714	Glycoside Hydrolase Family 28
	BACOVA_02715	Glycoside Hydrolase Family 95
	BACOVA_02716	

		BACOVA_02717	Glycoside Hydrolase Family 2
		BACOVA_02718	Glycoside Hydrolase Family 105
		BACOVA_02719	Polysaccharide Lyase Family 11
50	ECF	BACOVA_02727	
		BACOVA_02728	
		BACOVA_02729	susC-like
		BACOVA_02730	susD-like
		BACOVA_02731	
		BACOVA_02732	
		BACOVA_02733	
		BACOVA_02734	
		BACOVA_02735	
51	HTCS	BACOVA_02738	Glycoside Hydrolase Family 3
		BACOVA_02739	
		BACOVA_02740	
		BACOVA_02741	Glycoside Hydrolase Family 16
		BACOVA_02742	susC-like
		BACOVA_02743	susD-like
		BACOVA_02744	
		BACOVA_02745	Glycoside Hydrolase Family 3
		BACOVA_02746	
		BACOVA_02747	
52	HTCS	BACOVA_02748	Carbohydrate Esterase 6
		BACOVA_02749	
		BACOVA_02750	
		BACOVA_02751	susD-like
		BACOVA_02752	susC-like
		BACOVA_02753	Glycoside Hydrolase Family 3
		BACOVA_02754	
		BACOVA_02755	Glycoside Hydrolase Family 36
		BACOVA_02756	
		BACOVA_02757	
53	SusR	BACOVA_02782	
		BACOVA_02783	Glycoside Hydrolase Family 31
		BACOVA_02784	Glycoside Hydrolase Family 66
		BACOVA_02785	

	BACOVA_02786	susD-like
	BACOVA_02787	susC-like
	BACOVA_02788	
54 HTCS	BACOVA_02795	Glycoside Hydrolase Family 2
	BACOVA_02796	
	BACOVA_02797	Glycoside Hydrolase Family 43
	BACOVA_02798	
	BACOVA_02799	Glycoside Hydrolase Family 51
	BACOVA_02800	
	BACOVA_02801	
	BACOVA_02802	
	BACOVA_02803	susC-like
	BACOVA_02804	susD-like
	BACOVA_02805	susC-like
	BACOVA_02806	Glycoside Hydrolase Family 43
	BACOVA_02807	
	BACOVA_02808	
	BACOVA_02809	
	BACOVA_02810	
	BACOVA_02811	
55 unknown	BACOVA_02816	
	BACOVA_02817	susC-like
	BACOVA_02818	susD-like
	BACOVA_02819	
56 ECF	BACOVA_02845	
	BACOVA_02846	
	BACOVA_02847	susC-like
	BACOVA_02848	susD-like
	BACOVA_02849	
	BACOVA_02850	
	BACOVA_02851	
	BACOVA_02852	
	BACOVA_02853	
57 HTCS	BACOVA_02857	
	BACOVA_02858	Carbohydrate Esterase 6
	BACOVA_02859	susC-like

		BACOVA_02860	susD-like
		BACOVA_02861	Polysaccharide Lyase Family 15
		BACOVA_02862	
		BACOVA_02863	Glycoside Hydrolase Family 88
		BACOVA_02864	
		BACOVA_02865	
58	HTCS	BACOVA_02881	
		BACOVA_02882	
		BACOVA_02883	susD-like
		BACOVA_02884	susC-like
		BACOVA_02885	
		BACOVA_02886	
		BACOVA_02887	
59	ECF	BACOVA_02943	
		BACOVA_02944	
		BACOVA_02945	susC-like
		BACOVA_02946	susD-like
		BACOVA_02947	
		BACOVA_02948	
		BACOVA_02949	Glycoside Hydrolase Family 5
		BACOVA_02950	Glycoside Hydrolase Family 20
60	none	BACOVA_02951	
		BACOVA_02952	
		BACOVA_02953	
		BACOVA_02954	susC-like
		BACOVA_02955	susD-like
		BACOVA_02956	Glycoside Hydrolase Family 43
		BACOVA_02957	
		BACOVA_02958	Glycoside Hydrolase Family 3
		BACOVA_02959	Glycoside Hydrolase Family 97
61	unknown	BACOVA_03062	susD-like
		BACOVA_03063	susC-like
62	unknown	BACOVA_03078	susD-like
		BACOVA_03079	susC-like
		BACOVA_03080	
		BACOVA_03081	

	BACOVA_03082	susC-like	
	BACOVA_03083	susD-like	
63 HTCS	BACOVA_03096		
	BACOVA_03097	susC-like	
	BACOVA_03098	susD-like	
	BACOVA_03099		
	BACOVA_03100	Glycoside Hydrolase Family 2	
	BACOVA_03101	Glycoside Hydrolase Family 50	
	BACOVA_03102	Glycoside Hydrolase Family 50	
64 ECF	BACOVA_03118	Glycoside Hydrolase Family 106	
	BACOVA_03119		
	BACOVA_03120	Glycoside Hydrolase Family 78	
	BACOVA_03121	Glycoside Hydrolase Family 20	
	BACOVA_03122		
	BACOVA_03123		
	BACOVA_03124		
	BACOVA_03125	susD-like	
	BACOVA_03126		
	BACOVA_03127	susC-like	
	BACOVA_03128		
	BACOVA_03129		
65 unknown	BACOVA_03153	susD-like	bacova_03153-54 are induced *in vivo* in plant fed mice
	BACOVA_03154	susC-like	
66 unknown	BACOVA_03171		
	BACOVA_03172		
	BACOVA_03173		
	BACOVA_03174		
	BACOVA_03175		
	BACOVA_03176		
67 GntR	BACOVA_03251		
	BACOVA_03252		
	BACOVA_03253	susC-like	
	BACOVA_03254	susD-like	
	BACOVA_03255		
	BACOVA_03256	Glycoside Hydrolase Family 76	

	BACOVA_03257	Glycoside Hydrolase Family 76	
	BACOVA_03258	Glycoside Hydrolase Family 92	
	BACOVA_03259		
68 ECF	BACOVA_03283		
	BACOVA_03284		
	BACOVA_03285		
	BACOVA_03286		
	BACOVA_03287	susC-like	
	BACOVA_03288	susD-like	
	BACOVA_03289		
69 unknown	BACOVA_03317		
	BACOVA_03318		
	BACOVA_03319		
	BACOVA_03320	susC-like	
	BACOVA_03321	susD-like	
	BACOVA_03322		
70 ECF	BACOVA_03337		
	BACOVA_03338		
	BACOVA_03339	susC-like	
	BACOVA_03340	susD-like	
	BACOVA_03341	Glycoside Hydrolase Family 76	
	BACOVA_03342		
	BACOVA_03343		
	BACOVA_03344	Glycoside Hydrolase Family 76	
	BACOVA_03345		
	BACOVA_03346	Glycoside Hydrolase Family 92	
	BACOVA_03347	Glycoside Hydrolase Family 125	
71 unknown	BACOVA_03379		bacova_03379-82 are induced *in vivo* in plant fed mice
	BACOVA_03380	Glycoside Hydrolase Family 3	
	BACOVA_03381	susD-like	
	BACOVA_03382	susC-like	
72 AraC	BACOVA_03386		
	BACOVA_03387		
	BACOVA_03388		
	BACOVA_03389		

	BACOVA_03390	Glycoside Hydrolase Family 109
	BACOVA_03391	
	BACOVA_03392	Glycoside Hydrolase Family 109
	BACOVA_03393	
	BACOVA_03394	
	BACOVA_03395	
	BACOVA_03396	
	BACOVA_03397	Carbohydrate Esterase 9
	BACOVA_03398	
	BACOVA_03399	Glycoside Hydrolase Family 3
	BACOVA_03400	Glycoside Hydrolase Family 26
	BACOVA_03401	
	BACOVA_03402	susD-like
	BACOVA_03403	susC-like
	BACOVA_03404	
	BACOVA_03405	
	BACOVA_03406	
73 HTCS	BACOVA_03417	Glycoside Hydrolase Family 43
	BACOVA_03418	
	BACOVA_03419	Glycoside Hydrolase Family 3
	BACOVA_03420	
	BACOVA_03421	Glycoside Hydrolase Family 43
	BACOVA_03422	Glycoside Hydrolase Family 31
	BACOVA_03423	Glycoside Hydrolase Family 97
	BACOVA_03424	Glycoside Hydrolase Family 43-Carbohydrate Binding Module 6
	BACOVA_03425	Glycoside Hydrolase Family 43
	BACOVA_03426	susC-like
	BACOVA_03427	susD-like
	BACOVA_03428	susC-like
	BACOVA_03429	susD-like
	BACOVA_03430	
	BACOVA_03431	Glycoside Hydrolase Family 10
	BACOVA_03432	Glycoside Hydrolase Family 30
	BACOVA_03433	Glycoside Hydrolase Family 98-Carbohydrate Binding Module 35

	BACOVA_03434	Glycoside Hydrolase Family 115	
	BACOVA_03435	Carbohydrate Esterase 6-Carbohydrate Esterase 1	
	BACOVA_03436	Glycoside Hydrolase Family 43-Carbohydrate Binding Module 6	
	BACOVA_03437		
	BACOVA_03438	Glycoside Hydrolase Family 95	
	BACOVA_03439		
	BACOVA_03440		
	BACOVA_03441		
	BACOVA_03442		
	BACOVA_03443	susC-like	
	BACOVA_03444	susD-like	
	BACOVA_03445		
	BACOVA_03446		
	BACOVA_03447		
	BACOVA_03448		
	BACOVA_03449	Glycoside Hydrolase Family 115	
	BACOVA_03450	Carbohydrate Esterase 6	
74	BACOVA_03466		
	BACOVA_03467		
	BACOVA_03468		
	BACOVA_03469		
75 HTCS	BACOVA_03504		
	BACOVA_03505		
	BACOVA_03506	susC-like	
	BACOVA_03507	susD-like	
	BACOVA_03508	Glycoside Hydrolase Family 16	
	BACOVA_03509		
	BACOVA_03510		
	BACOVA_03511	Glycoside Hydrolase Family 2	
76 SusR	BACOVA_03514	Glycoside Hydrolase Family 13	bacova_03514-20 are induced *in vivo* in plant fed mice
	BACOVA_03515		This PUL is homologous to *B. thetaiotaomicron* starch
	BACOVA_03516		utilization PUL.

	BACOVA_03517	susD-like	
	BACOVA_03518	susC-like	
	BACOVA_03519	Glycoside Hydrolase Family 97	
	BACOVA_03520	Glycoside Hydrolase Family 13	
	BACOVA_03521		
77 ECF	BACOVA_03554		
	BACOVA_03555		
	BACOVA_03556	susC-like	bacova_03556-59 are induced *in vivo* in plant fed mice
	BACOVA_03557	susD-like	This PUL is homologous to a *B. thetaiotaomicron* PUL
	BACOVA_03558	Glycoside Hydrolase Family 18	associated with host glycan utilization *in vitro* and *in vivo*.
	BACOVA_03559		
78 HTCS	BACOVA_03562		
	BACOVA_03563		
	BACOVA_03564		
	BACOVA_03565	susC-like	
	BACOVA_03566	susD-like	
	BACOVA_03567		
	BACOVA_03568		
	BACOVA_03569	Carbohydrate Esterase 7	
79 none	BACOVA_03576	Carbohydrate Esterase 8	
	BACOVA_03577	Glycoside Hydrolase Family 95	
	BACOVA_03578	susC-like	
	BACOVA_03579	susD-like	
	BACOVA_03580		
	BACOVA_03581	Carbohydrate Esterase 8	
	BACOVA_03582		
80 ECF	BACOVA_03594		
	BACOVA_03595		
	BACOVA_03596	susC-like	
	BACOVA_03597	susD-like	
	BACOVA_03598		
	BACOVA_03599		
81 HTCS	BACOVA_03607		

		BACOVA_03608	susC-like
		BACOVA_03609	susD-like
		BACOVA_03610	
		BACOVA_03611	
		BACOVA_03612	
		BACOVA_03613	Glycoside Hydrolase Family 50
		BACOVA_03614	
		BACOVA_03615	
		BACOVA_03616	Glycoside Hydrolase Family 31
		BACOVA_03617	Glycoside Hydrolase Family 2
		BACOVA_03618	Carbohydrate Esterase 6
82	HTCS	BACOVA_03623	Glycoside Hydrolase Family 92
		BACOVA_03624	
		BACOVA_03625	
		BACOVA_03626	Glycoside Hydrolase Family 125
		BACOVA_03627	Glycoside Hydrolase Family 76
		BACOVA_03628	
		BACOVA_03629	Glycoside Hydrolase Family 92
		BACOVA_03630	
		BACOVA_03631	
		BACOVA_03632	
		BACOVA_03633	Glycoside Hydrolase Family 76
		BACOVA_03634	susC-like
		BACOVA_03635	susD-like
		BACOVA_03636	
83	HTCS	BACOVA_03638	
		BACOVA_03639	Glycoside Hydrolase Family 36
		BACOVA_03640	Glycoside Hydrolase Family 29
		BACOVA_03641	
		BACOVA_03642	
		BACOVA_03643	susD-like
		BACOVA_03644	susC-like
		BACOVA_03645	
		BACOVA_03646	
84	ECF	BACOVA_03855	
		BACOVA_03856	

	BACOVA_03857	susC-like (frameshift w/ _03858)	
	BACOVA_03858	susC-like	
	BACOVA_03859	susD-like	
	BACOVA_03860		
	BACOVA_03861		
	BACOVA_03862	Glycoside Hydrolase Family 18	
85 ECF	BACOVA_04092		
	BACOVA_04093		
	BACOVA_04094		bacova_04094-99 and bacova_04103-111 are induced *in vivo* in plant fed mice. This PUL is homologous to a *B. thetaiotaomicron* PUL associated with host glycan utilization *in vitro* and *in vivo*.
	BACOVA_04095		
	BACOVA_04096		
	BACOVA_04097	Glycoside Hydrolase Family 18	
	BACOVA_04098	susD-like	
	BACOVA_04099	susC-like	
	BACOVA_04100		
	BACOVA_04101		
	BACOVA_04102		
	BACOVA_04103	susC-like	(part of above PUL)
	BACOVA_04104	susD-like	
	BACOVA_04105	Glycoside Hydrolase Family 18	
	BACOVA_04106		
	BACOVA_04107		
	BACOVA_04108	Carbohydrate Binding Module 32	
	BACOVA_04109		
	BACOVA_04110		
	BACOVA_04111	Glycoside Hydrolase Family 92	
86 HTCS	BACOVA_04114		
	BACOVA_04115	susC-like	
	BACOVA_04116	susD-like	
	BACOVA_04117	Polysaccharide Lyase Family 1	
	BACOVA_04118		
	BACOVA_04119	Glycoside Hydrolase Family 43	
	BACOVA_04120		

		BACOVA_04121	Glycoside Hydrolase Family 78
		BACOVA_04122	Glycoside Hydrolase Family 28
		BACOVA_04123	
		BACOVA_04124	
		BACOVA_04125	
		BACOVA_04126	Glycoside Hydrolase Family 78-<br>Glycoside Hydrolase Family 33
		BACOVA_04127	
		BACOVA_04128	Glycoside Hydrolase Family 105
		BACOVA_04129	Glycoside Hydrolase Family 95
		BACOVA_04130	
		BACOVA_04131	
		BACOVA_04132	
87	ECF	BACOVA_04146	
		BACOVA_04147	
		BACOVA_04148	susD-like
		BACOVA_04149	susC-like
		BACOVA_04150	
		BACOVA_04151	
88	GntR	BACOVA_04181	Glycoside Hydrolase Family 3
		BACOVA_04182	
		BACOVA_04183	
		BACOVA_04184	
		BACOVA_04185	susD-like
		BACOVA_04186	susC-like
		BACOVA_04187	
		BACOVA_04188	Glycoside Hydrolase Family 33
		BACOVA_04189	
		BACOVA_04190	
		BACOVA_04191	
89	unknown	BACOVA_04248	
		BACOVA_04249	
		BACOVA_04250	
		BACOVA_04251	
		BACOVA_04252	susD-like
		BACOVA_04253	susC-like

90	unknown	BACOVA_04292	
		BACOVA_04293	susC-like
		BACOVA_04294	susD-like
91	HTCS	BACOVA_04356	
		BACOVA_04357	Glycoside Hydrolase Family 29-Carbohydrate Binding Module 32
		BACOVA_04358	Glycoside Hydrolase Family 2
		BACOVA_04359	Glycoside Hydrolase Family 20
		BACOVA_04360	
		BACOVA_04361	susD-like
		BACOVA_04362	susC-like
		BACOVA_04363	
		BACOVA_04364	
		BACOVA_04365	
		BACOVA_04366	
		BACOVA_04367	
		BACOVA_04368	
92	ECF	BACOVA_04376	
		BACOVA_04377	susD-like
		BACOVA_04378	susC-like
		BACOVA_04379	
		BACOVA_04380	
93	HTCS	BACOVA_04385	Glycoside Hydrolase Family 67
		BACOVA_04386	Glycoside Hydrolase Family 43
		BACOVA_04387	Glycoside Hydrolase Family 10
		BACOVA_04388	
		BACOVA_04389	Carbohydrate Esterase6-Carbohydrate Esterase6
		BACOVA_04390	Glycoside Hydrolase Family 10-Carbohydrate Binding Module 22
		BACOVA_04391	
		BACOVA_04392	susD-like
		BACOVA_04393	susC-like
		BACOVA_04394	
94	ECF	BACOVA_04399	
		BACOVA_04400	

		BACOVA_04401		
		BACOVA_04402	susC-like	
		BACOVA_04403	susD-like	
		BACOVA_04404		
		BACOVA_04405		
		BACOVA_04406		
95	unknown	BACOVA_04427	susD-like	
		BACOVA_04428	susC-like	
96	HTCS	BACOVA_04477	Polysaccharide Lyase Family 12	
		BACOVA_04478	susD-like	
		BACOVA_04479	susC-like	
		BACOVA_04480	susD-like	
		BACOVA_04481	susC-like	
		BACOVA_04482	Glycoside Hydrolase Family 29	
		BACOVA_04483		
		BACOVA_04484	Glycoside Hydrolase Family 88	
97	HTCS	BACOVA_04496		
		BACOVA_04497		
		BACOVA_04498		
		BACOVA_04499		
		BACOVA_04500		bacova_04497 and bacova_04500-07 are induced *in vivo.*
		BACOVA_04501	Glycoside Hydrolase Family 32	This PUL is homologous to *B. thetaiotaomicron* fructan
		BACOVA_04502	Glycoside Hydrolase Family 91	utilization PUL, but likely has specificity for inulin rather
		BACOVA_04503	Glycoside Hydrolase Family 91	than levan.
		BACOVA_04504	susD-like	
		BACOVA_04505	susC-like	
		BACOVA_04506		
		BACOVA_04507	Glycoside Hydrolase Family 32	
98	ECF	BACOVA_04799		bacova_04799-4811 are induced *in vivo* in plant fed mice
		BACOVA_04800	Glycoside Hydrolase Family 18	This PUL is homologous to a *B. thetaiotaomicron* PUL

			associated with host glycan utilization *in vitro* and *in vivo*.
	BACOVA_04801		
	BACOVA_04802	Glycoside Hydrolase Family 18	
	BACOVA_04803	susD-like	
	BACOVA_04804	susC-like	
	BACOVA_04805		
	BACOVA_04806		
	BACOVA_04807	Glycoside Hydrolase Family 18	
	BACOVA_04808		
	BACOVA_04809	Glycoside Hydrolase Family 92	
	BACOVA_04810	Glycoside Hydrolase Family 92	
	BACOVA_04811		
	BACOVA_04812		
	BACOVA_04813	Glycoside Hydrolase Family 92	
99 SusR	BACOVA_04864		
	BACOVA_04865		
	BACOVA_04866		
	BACOVA_04867		
	BACOVA_04868	Glycoside Hydrolase Family 38	
	BACOVA_04869	Glycoside Hydrolase Family 92	
	BACOVA_04870	Glycoside Hydrolase Family 2-Carbohydrate Binding Module 32	
	BACOVA_04871		
	BACOVA_04872	Glycoside Hydrolase Family 78	
	BACOVA_04873		
	BACOVA_04874	Glycoside Hydrolase Family 31	
	BACOVA_04875		
	BACOVA_04876	susC-like	
	BACOVA_04877	susD-like	
	BACOVA_04878	susC-like	
	BACOVA_04879		
	BACOVA_04880		
	BACOVA_04881		
	BACOVA_04882		
	BACOVA_04883	Glycoside Hydrolase Family 92	
	BACOVA_04884	Glycoside Hydrolase Family 92	

100 HTCS	BACOVA_04900	Glycoside Hydrolase Family 105
	BACOVA_04901	Carbohydrate Esterase8
	BACOVA_04902	Carbohydrate Esterase12-Carbohydrate Esterase 8
	BACOVA_04903	
	BACOVA_04904	
	BACOVA_04905	Polysaccharide Lyase Family 1
	BACOVA_04906	Polysaccharide Lyase Family 1
	BACOVA_04907	Polysaccharide Lyase Family 1
	BACOVA_04908	Carbohydrate Esterase 8
	BACOVA_04909	
	BACOVA_04910	susD-like
	BACOVA_04911	susC-like
	BACOVA_04912	
	BACOVA_04913	
	BACOVA_04914	
	BACOVA_04915	
	BACOVA_04916	Polysaccharide Lyase Family 1
	BACOVA_04917	
	BACOVA_04918	
	BACOVA_04919	
	BACOVA_04920	Polysaccharide Lyase Family 1
	BACOVA_04921	susC-like
	BACOVA_04922	susD-like
	BACOVA_04923	
	BACOVA_04924	
	BACOVA_04925	Carbohydrate Esterase 8
	BACOVA_04926	Glycoside Hydrolase Family 28
	BACOVA_04927	Glycoside Hydrolase Family 3
	BACOVA_04928	
	BACOVA_04929	Glycoside Hydrolase Family 43-Carbohydrate Binding Module 6
	BACOVA_04930	
	BACOVA_04931	
101 HTCS	BACOVA_04945	Glycoside Hydrolase Family 106
	BACOVA_04946	Glycoside Hydrolase Family 28

	BACOVA_04947	
	BACOVA_04948	Glycoside Hydrolase Family 28
	BACOVA_04949	Carbohydrate Esterase12
	BACOVA_04950	Glycoside Hydrolase Family 2
	BACOVA_04951	Glycoside Hydrolase Family 43-Carbohydrate Binding Module 32
	BACOVA_04952	Glycoside Hydrolase Family 42
	BACOVA_04953	
	BACOVA_04954	Glycoside Hydrolase Family 28
	BACOVA_04955	
	BACOVA_04956	susD-like
	BACOVA_04957	susC-like
	BACOVA_04958	
	BACOVA_04959	
	BACOVA_04960	
	BACOVA_04961	
	BACOVA_04962	susC-like
	BACOVA_04963	susD-like
	BACOVA_04964	susC-like
	BACOVA_04965	susD-like
	BACOVA_04966	Polysaccharide Lyase Family 11
	BACOVA_04967	
	BACOVA_04968	
	BACOVA_04969	
	BACOVA_04970	
	BACOVA_04971	
	BACOVA_04972	Carbohydrate Esterase12
	BACOVA_04973	Glycoside Hydrolase Family 105
	BACOVA_04974	Polysaccharide Lyase Family 11
	BACOVA_04975	Glycoside Hydrolase Family 105
	BACOVA_04976	
	BACOVA_04977	
	BACOVA_04978	Carbohydrate Esterase 4
	BACOVA_04979	Glycoside Hydrolase Family 105
102 HTCS	BACOVA_04985	
	BACOVA_04986	

		BACOVA_04987	
		BACOVA_04988	susD-like
		BACOVA_04989	susC-like
		BACOVA_04990	
		BACOVA_04991	
		BACOVA_04992	Glycoside Hydrolase Family 2
		BACOVA_04993	
		BACOVA_04994	
		BACOVA_04995	Polysaccharide Lyase Family 11
		BACOVA_04996	
		BACOVA_04997	Glycoside Hydrolase Family 28
		BACOVA_04998	
		BACOVA_04999	
		BACOVA_05000	Polysaccharide Lyase Family 9
		BACOVA_05001	
		BACOVA_05002	Glycoside Hydrolase Family 28
		BACOVA_05003	
		BACOVA_05004	Carbohydrate Esterase 6-Glycoside Hydrolase Family 105
		BACOVA_05005	Polysaccharide Lyase Family 1
		BACOVA_05006	
		BACOVA_05007	susC-like
		BACOVA_05008	susD-like
		BACOVA_05009	
		BACOVA_05010	
		BACOVA_05011	susC-like
		BACOVA_05012	susD-like
		BACOVA_05013	
		BACOVA_05014	
		BACOVA_05015	Glycoside Hydrolase Family 28
		BACOVA_05016	
103	ECF	BACOVA_05141	Glycoside Hydrolase Family 20-Carbohydrate Binding Module 32
		BACOVA_05142	
		BACOVA_05143	
		BACOVA_05144	

		BACOVA_05145	
		BACOVA_05146	
		BACOVA_05147	
		BACOVA_05148	susD-like
		BACOVA_05149	susC-like
		BACOVA_05150	
		BACOVA_05151	
104	ECF	BACOVA_05174	
		BACOVA_05175	
		BACOVA_05176	
		BACOVA_05177	
		BACOVA_05178	susC-like
		BACOVA_05179	susD-like
		BACOVA_05180	
		BACOVA_05181	
		BACOVA_05182	Glycoside Hydrolase Family 35-Carbohydrate Binding Module 32
		BACOVA_05183	Glycoside Hydrolase Family 89
105	ECF	BACOVA_05184	susD-like
		BACOVA_05185	
		BACOVA_05186	susC-like
		BACOVA_05187	
		BACOVA_05188	
		BACOVA_05189	
106	unknown	BACOVA_05278	susC-like
		BACOVA_05279	susD-like
		BACOVA_05280	
107	HTCS	BACOVA_05466	
		BACOVA_05467	
		BACOVA_05468	Polysaccharide Lyase Family 15
		BACOVA_05469	
		BACOVA_05470	
		BACOVA_05471	
		BACOVA_05472	
		BACOVA_05473	Polysaccharide Lyase Family 12
		BACOVA_05474	Glycoside Hydrolase Family 88

		BACOVA_05475	susD-like	
		BACOVA_05476	susC-like	
		BACOVA_05477		
		BACOVA_05478	Polysaccharide Lyase Family 12	
		BACOVA_05479		
		BACOVA_05497	Polysaccharide Lyase Family 13	
108	HTCS	BACOVA_05487	Glycoside Hydrolase Family 2	
				bacova_05488-91 and bacova_05493
		BACOVA_05488	Glycoside Hydrolase Family 53	are induced *in vivo*
		BACOVA_05489		in plant fed mice.
		BACOVA_05490	susD-like	
		BACOVA_05491	susC-like	
		BACOVA_05492		
		BACOVA_05493	Glycoside Hydrolase Family 5	
		BACOVA_05494		
		BACOVA_05495		
109	unknown	BACOVA_05542	susC-like	
		BACOVA_05543	susD-like	
110	ECF	BACOVA_05556		
		BACOVA_05557		
		BACOVA_05558		
		BACOVA_05559	susC-like	
		BACOVA_05560	susD-like	
		BACOVA_05561	Glycoside Hydrolase Family 18	
		BACOVA_05562		
		BACOVA_05563		
		BACOVA_05564		
		BACOVA_05565	Glycoside Hydrolase Family 3	
111	ECF	BACOVA_05573		
		BACOVA_05574		
		BACOVA_05575		
		BACOVA_05576	susC-like	
		BACOVA_05577	susD-like	
		BACOVA_05578		
		BACOVA_05579		
		BACOVA_05580		

	BACOVA_05581	
112 HTCS	BACOVA_05611	Glycoside Hydrolase Family 28
	BACOVA_05612	
	BACOVA_05613	Carbohydrate Binding Module 6
	BACOVA_05614	
	BACOVA_05615	
	BACOVA_05616	
	BACOVA_05617	
	BACOVA_05618	susC-like
	BACOVA_05619	susD-like
	BACOVA_05620	
	BACOVA_05621	
	BACOVA_05622	
