# Supplementary material for: Recognition and Degradation of Plant Cell Wall Polysaccharides by Two Human Gut Symbionts
Source: PLoS Biol. 2011 Dec 20;9(12):e1001221. doi: 10.1371/journal.pbio.1001221 (PMC3243724; doi:10.1371/journal.pbio.1001221)
Supplement: Table S6 — Comparison of homologous PULs between B. thetaiotaomicron and B. ovatus. Dark green lines delineate “homologous PULs.” Light green lines delineate “probably homologous PULs.” Yellow lines delineate genes immediately adjacent to PULs that are syntenic best Blast hits. Gray lines delineate genes within homologous PULs that are not best Blast hits. (PDF) [file pbio.1001221.s015.pdf]

Table S6. Comparison of homologous PULs between *B. thetaiotaomicron* (*Bt*) and *B. ovatus* (*Bo*)

Dark green lines delineate "homologous PULs"
Light green lines delineate "probably homologous PULs"
Yellow lines delineate gene immediately adjacent to PULs that are syntenic best Blast hits
Gray lines delineate genes within homologous PULs that are not best Blast hits

Homologous PUL number	*Bt* gene	*Bo* gene
1	BT0139	BACOVA_01298
	BT0140	BACOVA_01297
	BT0141	BACOVA_03566
	BT0142	BACOVA_03567
	BT0143	BACOVA_01294
	BT0145	BACOVA_01293
	BT0146	BACOVA_01292
	BT0147	BACOVA_01290
	BT0148	BACOVA_01289
	BT0149	BACOVA_01288
	BT0150	BACOVA_01287
	BT0151	BACOVA_01286
	BT0152	BACOVA_01285
	BT0153	BACOVA_01284
	BT0154	BACOVA_01283
	BT0155	BACOVA_01282
	BT0156	BACOVA_01281
	BT0178	BACOVA_01262
	BT0179	BACOVA_01261
	BT0180	BACOVA_01260
	BT0181	BACOVA_01259
	BT0182	BACOVA_01257
	BT0183	BACOVA_01256
	BT0184	BACOVA_01255
	BT0185	BACOVA_01254
	BT0186	BACOVA_01253
	BT0187	BACOVA_01252
2	BT0188	BACOVA_01251
	BT0189	BACOVA_01250
	BT0190	BACOVA_01248
	BT0191	BACOVA_01247
	BT0192	BACOVA_01246
	BT0193	BACOVA_01245
	BT0194	BACOVA_01244
	BT0195	BACOVA_01243
	BT0196	BACOVA_05581
	BT0197	BACOVA_01239
	BT0198	BACOVA_01238
	BT0199	BACOVA_01237
	BT0200	BACOVA_01235
	BT0201	BACOVA_01234
	BT0202	BACOVA_01233

	BT0203	BACOVA_01232
	BT0204	BACOVA_01231
	BT0205	BACOVA_01230
3	BT0206	BACOVA_01228
	BT0207	BACOVA_01227
	BT0208	BACOVA_01226
	BT0209	BACOVA_01225
	BT0210	BACOVA_01224
	BT0212	BACOVA_00373
	BT0213	BACOVA_00374
	BT0214	BACOVA_00375
	BT0216	BACOVA_00378
	BT0217	BACOVA_00379
	BT0218	BACOVA_00380
	BT0219	BACOVA_00381

	BT0743	BACOVA_02478
	BT0744	BACOVA_02479
	BT0745	BACOVA_02480
	BT0746	BACOVA_02481
	BT0747	BACOVA_02482
	BT0748	BACOVA_02484
	BT0749	BACOVA_02485
	BT0750	BACOVA_02486
	BT0751	BACOVA_02487
4	BT0752	BACOVA_02488
	BT0753	BACOVA_02489
	BT0754	BACOVA_02490
	BT0755	BACOVA_02491
	BT0756	BACOVA_02492
	BT0757	BACOVA_02493

	BT0972	BACOVA_00778
	BT0973	BACOVA_00779
	BT0974	BACOVA_00780
	BT0975	BACOVA_00781
	BT0976	BACOVA_00782
	BT0977	BACOVA_00783
	BT0978	BACOVA_00784
5	BT0979	BACOVA_00785
	BT0980	BACOVA_00786
	BT0981	BACOVA_00787
	BT0983	BACOVA_00788
	BT0984	BACOVA_00162
	BT0985	BACOVA_00163
	BT0986	BACOVA_00164
	BT0987	BACOVA_01291
	BT0988	BACOVA_00178
	BT0989	BACOVA_00179
	BT0990	BACOVA_00180

	BT0992	BACOVA_00181
	BT0993	BACOVA_00182
	BT0996	BACOVA_00184
	BT0997	BACOVA_00268
	BT0998	BACOVA_00270
	BT0999	BACOVA_00271
	BT1000	BACOVA_02584
	BT1001	BACOVA_00285
	BT1002	BACOVA_00286
	BT1003	BACOVA_00287
	BT1004	BACOVA_00288
	BT1010	BACOVA_04129
	BT1011	BACOVA_04128
	BT1012	BACOVA_04127
	BT1013	BACOVA_04126
	BT1014	BACOVA_02700
	BT1015	BACOVA_04124
	BT1017	BACOVA_04123
	BT1018	BACOVA_04122
	BT1019	BACOVA_04121
	BT1020	BACOVA_04120
	BT1021	BACOVA_04119
	BT1022	BACOVA_04118
	BT1023	BACOVA_04117
	BT1028	BACOVA_04116
	BT1029	BACOVA_04115
	BT1030	BACOVA_04114
	BT1031	BACOVA_04113
6	BT1032	BACOVA_04111
	BT1033	BACOVA_04110
	BT1034	BACOVA_04109
	BT1035	BACOVA_04108
	BT1041	BACOVA_04100
	BT1046	BACOVA_04099
	BT1047	BACOVA_04098
	BT1048	BACOVA_04097
	BT1049	BACOVA_04096
	BT1050	BACOVA_04095
	BT1052	BACOVA_04093
	BT1053	BACOVA_04092
	BT1054	BACOVA_04090
	BT1055	BACOVA_04089
	BT1056	BACOVA_04088
	BT1057	BACOVA_04087
	BT1058	BACOVA_04086
	BT1059	BACOVA_04084
	BT1060	BACOVA_04083
	BT1061	BACOVA_04082
	BT1263	BACOVA_03843

	BT1264	BACOVA_03846
	BT1270	BACOVA_03847
	BT1271	BACOVA_03848
	BT1272	BACOVA_03849
	BT1273	BACOVA_03850
	BT1274	BACOVA_03851
	BT1275	BACOVA_03852
	BT1276	BACOVA_03853
	BT1277	BACOVA_03854
7	BT1278	BACOVA_03855
	BT1279	BACOVA_03856
	BT1280	BACOVA_03857/8 (frameshift)
	BT1281	BACOVA_03859
	BT1282	BACOVA_00158
	BT1283	BACOVA_01339
	BT1284	BACOVA_00159
	BT1286	BACOVA_03863
	BT1287	BACOVA_03864
8	BT1439	BACOVA_00865
	BT1440	BACOVA_00866
	BT1441	BACOVA_00867
	BT1442	BACOVA_00868
	BT1443	BACOVA_00871
	BT1444	BACOVA_00872
	BT1445	BACOVA_00873
	BT1446	BACOVA_00874
	BT1447	BACOVA_00875
	BT1448	BACOVA_00876
	BT1449	BACOVA_00877
	BT1541	BACOVA_04279
	BT1542	BACOVA_04280
	BT1543	BACOVA_04281
	BT1544	BACOVA_04284
	BT1545	BACOVA_04285
	BT1546	BACOVA_04287
	BT1547	BACOVA_04288
	BT1548	BACOVA_04289
	BT1549	BACOVA_04290
	BT1550	BACOVA_04291
9	BT1551	BACOVA_04292
	BT1552	BACOVA_04293
	BT1553	BACOVA_04294
	BT1554	BACOVA_04297
	BT1555	BACOVA_04298
	BT1556	BACOVA_04299
	BT1557	BACOVA_04300
	BT1558	BACOVA_04301
	BT1559	BACOVA_04302

	BT1560	BACOVA_04303
	BT1561	BACOVA_04304
	BT1562	BACOVA_04305
	BT1563	BACOVA_04306
	BT1672	BACOVA_04415
	BT1673	BACOVA_04416
	BT1674	BACOVA_04417
	BT1675	BACOVA_04419
	BT1676	BACOVA_04420
	BT1677	BACOVA_04421
	BT1678	BACOVA_04422
	BT1679	BACOVA_04423
	BT1680	BACOVA_04424
	BT1681	BACOVA_04426
10	BT1682	BACOVA_04427
	BT1683	BACOVA_04428
	BT1684	BACOVA_04429
	BT1685	BACOVA_04430
	BT1686	BACOVA_04431
	BT1687	BACOVA_04432
	BT1688	BACOVA_04433
	BT1689	BACOVA_04434
	BT1691	BACOVA_04436
	BT1692	BACOVA_04437
	BT1745	BACOVA_04487
	BT1746	BACOVA_04489
	BT1747	BACOVA_04490
	BT1749	BACOVA_04491
	BT1750	BACOVA_04492
	BT1751	BACOVA_04493
	BT1753	BACOVA_04494
11	BT1754	BACOVA_04496
	BT1757	BACOVA_04498
	BT1758	BACOVA_04500
	BT1759	BACOVA_04501
	BT1762	BACOVA_04504
	BT1763	BACOVA_04505
	BT1764	BACOVA_04506
	BT1765	BACOVA_04507
12	BT2032	BACOVA_00682
	BT2033	BACOVA_00681
	BT2034	BACOVA_00680
	BT2035	BACOVA_00679
	BT2037	BACOVA_00677
	BT2038	BACOVA_00676
	BT2039	BACOVA_00675
	BT2040	BACOVA_00674

	BT2041	BACOVA_00672
	BT2042	BACOVA_00671
	BT2043	BACOVA_00670
	BT2159	BACOVA_03187
	BT2160	BACOVA_03186
	BT2161	BACOVA_03185
	BT2162	BACOVA_03184
	BT2163	BACOVA_03183
	BT2164	BACOVA_03182
	BT2165	BACOVA_03180
	BT2166	BACOVA_03179
	BT2167	BACOVA_03178
	BT2168	BACOVA_03177
13	BT2169	BACOVA_03176
	BT2170	BACOVA_03175
	BT2171	BACOVA_03173
	BT2172	BACOVA_03172
	BT2173	BACOVA_03171
	BT2176	BACOVA_03169
	BT2177	BACOVA_03168
	BT2178	BACOVA_03166
	BT2179	BACOVA_03165
	BT2180	BACOVA_03163
	BT2181	BACOVA_03162
	BT2182	BACOVA_03161
	BT2183	BACOVA_03159
	BT2186	BACOVA_03157
	BT2187	BACOVA_03156
14	BT3086	BACOVA_02783
	BT3087	BACOVA_02784
	BT3088	BACOVA_02785
	BT3089	BACOVA_02786
	BT3090	BACOVA_02787
	BT3091	BACOVA_02788
15	BT3309	BACOVA_00941
	BT3310	BACOVA_00942
	BT3311	BACOVA_00943
	BT3312	BACOVA_00944
	BT3313	BACOVA_00945
	BT3314	BACOVA_00946
16	BT3328	BACOVA_01962
	BT3329	BACOVA_01963
	BT3330	BACOVA_02000
	BT3331	BACOVA_01999
	BT3332	BACOVA_01998
	BT3333	BACOVA_01997

	BT3334	BACOVA_01996
	BT3335	BACOVA_01995
	BT3336	BACOVA_01994
	BT3337	BACOVA_01993
	BT3339	BACOVA_01991
	BT3340	BACOVA_01990
17	BT3515	BACOVA_03334
	BT3516	BACOVA_03336
	BT3517	BACOVA_03337
	BT3518	BACOVA_03338
	BT3519	BACOVA_03339
	BT3520	BACOVA_03340
	BT3521	BACOVA_03341
	BT3522	BACOVA_03342
	BT3523	BACOVA_03343
	BT3524	BACOVA_03344
	BT3526	BACOVA_03345
	BT3527	BACOVA_03346
	BT3528	BACOVA_03347
	BT3529	BACOVA_03348
	BT3554	BACOVA_03364
	BT3555	BACOVA_03365
	BT3558	BACOVA_03367
	BT3559	BACOVA_03368
	BT3560	BACOVA_03369
	BT3561	BACOVA_03370
	BT3562	BACOVA_03371
	BT3563	BACOVA_03373
	BT3564	BACOVA_03377
	BT3565	BACOVA_03378
18	BT3566	BACOVA_03379
	BT3567	BACOVA_03380
	BT3568	BACOVA_03381
	BT3569	BACOVA_03382
	BT3570	BACOVA_02919
	BT3576	BACOVA_03384
	BT3577	BACOVA_03386
	BT3578	BACOVA_03387
	BT3579	BACOVA_03388
	BT3581	BACOVA_03390
	BT3582	BACOVA_03391
	BT3583	BACOVA_03392
	BT3584	BACOVA_03393
	BT3585	BACOVA_03394
	BT3586	BACOVA_03395
	BT3620	BACOVA_03455
	BT3621	BACOVA_03456

	BT3622	BACOVA_03457
	BT3623	BACOVA_03458
	BT3624	BACOVA_03459
	BT3625	BACOVA_03461
	BT3626	BACOVA_03462
	BT3627	BACOVA_03463
	BT3628	BACOVA_03464
	BT3629	BACOVA_03465
19	BT3630	BACOVA_03466
	BT3631	BACOVA_03467
	BT3632	BACOVA_03468
	BT3633	BACOVA_03469

	BT3696	BACOVA_03512
	BT3697	BACOVA_03513
20	BT3699	BACOVA_03515
	BT3700	BACOVA_03516
	BT3701	BACOVA_03517
	BT3702	BACOVA_03518
	BT3703	BACOVA_03519
	BT3704	BACOVA_03520
	BT3705	BACOVA_03521
	BT3708	BACOVA_03522
	BT3709	BACOVA_03523
	BT3710	BACOVA_03524
	BT3711	BACOVA_03527
	BT3712	BACOVA_03528
	BT3713	BACOVA_03529
	BT3714	BACOVA_03530
	BT3715	BACOVA_03531
	BT3716	BACOVA_03532
	BT3717	BACOVA_03533

21	BT3983	BACOVA_04804
	BT3985	BACOVA_04805
	BT3986	BACOVA_04806
	BT3987	BACOVA_04807
	BT3988	BACOVA_04808
	BT3990	BACOVA_04809
	BT3991	BACOVA_04810
	BT3992	BACOVA_04811
	BT3993	BACOVA_04812
	BT3994	BACOVA_04813
	BT3995	BACOVA_04814
	BT3996	BACOVA_04815
	BT3997	BACOVA_04816
	BT3998	BACOVA_04817
	BT3999	BACOVA_04818
	BT4000	BACOVA_04819
	BT4001	BACOVA_04820

	BT4002	BACOVA_04821
	BT4003	BACOVA_04822
	BT4004	BACOVA_04823
	BT4058	BACOVA_04853
	BT4059	BACOVA_04854
	BT4060	BACOVA_04855
	BT4061	BACOVA_04856
	BT4062	BACOVA_04857
	BT4063	BACOVA_04858
	BT4064	BACOVA_04859
	BT4065	BACOVA_04860
	BT4066	BACOVA_04861
	BT4067	BACOVA_04862
22	BT4069	BACOVA_04864
	BT4070	BACOVA_04866
	BT4071	BACOVA_04867
	BT4072	BACOVA_04868
	BT4073	BACOVA_04869
	BT4074	BACOVA_04870
	BT4075	BACOVA_04871
	BT4076	BACOVA_04872
	BT4077	BACOVA_02057
	BT4084	BACOVA_04882
	BT4085	BACOVA_04881
	BT4086	BACOVA_04880
	BT4087	BACOVA_04879
	BT4088	BACOVA_04878
	BT4089	BACOVA_04877
	BT4090	BACOVA_04876
	BT4091	BACOVA_02638
	BT4092	BACOVA_04883
	BT4093	BACOVA_04884
	BT4094	BACOVA_04885
	BT4096	BACOVA_04886
	BT4097	BACOVA_04887
	BT4098	BACOVA_04888
	BT4099	BACOVA_04889
	BT4100	BACOVA_04890
	BT4101	BACOVA_04892
	BT4102	BACOVA_04893
	BT4103	BACOVA_04894
	BT4104	BACOVA_04895
	BT4105	BACOVA_04896
	BT4106	BACOVA_04897
	BT4107	BACOVA_04898
23	BT4108	BACOVA_04900
	BT4109	BACOVA_04901
	BT4110	BACOVA_04902
	BT4111	BACOVA_04904

	BT4112	BACOVA_03580
	BT4113	BACOVA_04922
	BT4114	BACOVA_04911
	BT4115	BACOVA_04905
	BT4117	BACOVA_04913
	BT4118	BACOVA_04914
	BT4120	BACOVA_04923
	BT4123	BACOVA_04926
	BT4124	BACOVA_04931
	BT4125	BACOVA_04934
	BT4126	BACOVA_04935
	BT4127	BACOVA_04936
	BT4128	BACOVA_04938
	BT4129	BACOVA_04939
	BT4130	BACOVA_04940
	BT4131	BACOVA_04941
24	BT4402	BACOVA_03554
	BT4403	BACOVA_03555
	BT4404	BACOVA_03556
	BT4405	BACOVA_03557
	BT4406	BACOVA_03558
	BT4407	BACOVA_03559
	BT4468	BACOVA_05275
	BT4469	BACOVA_05276
25	BT4470	BACOVA_05278
	BT4471	BACOVA_05279
	BT4472	BACOVA_05280
	BT4473	BACOVA_05281
	BT4474	BACOVA_05282
	BT4475	BACOVA_05284
	BT4476	BACOVA_05285
	BT4477	BACOVA_05286
	BT4482	BACOVA_05288
	BT4483	BACOVA_05289
	BT4484	BACOVA_05290
	BT4485	BACOVA_05291
	BT4486	BACOVA_05292
	BT4487	BACOVA_05293
	BT4642	BACOVA_05456
	BT4643	BACOVA_05457
	BT4644	BACOVA_05458
	BT4645	BACOVA_05459
	BT4646	BACOVA_05460
	BT4647	BACOVA_05461
	BT4648	BACOVA_05462
	BT4649	BACOVA_05463
	BT4650	BACOVA_05465

	BT4651	BACOVA_05467
26	BT4652	BACOVA_05468
	BT4653	BACOVA_05469
	BT4654	BACOVA_05470
	BT4655	BACOVA_05471
	BT4656	BACOVA_05472
	BT4657	BACOVA_05473
	BT4658	BACOVA_05474
	BT4659	BACOVA_05475
	BT4660	BACOVA_05476
	BT4661	BACOVA_05477
	BT4662	BACOVA_05478
	BT4663	BACOVA_05479
	BT4664	BACOVA_05481
	BT4665	BACOVA_05482
	BT4666	BACOVA_05483
27	BT4667	BACOVA_05487
	BT4668	BACOVA_05488
	BT4669	BACOVA_05489
	BT4670	BACOVA_05490
	BT4671	BACOVA_05491
	BT4672	BACOVA_05493
	BT4673	BACOVA_05495
	BT4674	BACOVA_05496
	BT4675	BACOVA_05497

	BT4691	BACOVA_05545
	BT4696	BACOVA_05546
	BT4697	BACOVA_05547
	BT4698	BACOVA_05548
	BT4699	BACOVA_05549
	BT4700	BACOVA_05550
	BT4701	BACOVA_05551
	BT4702	BACOVA_05552
	BT4703	BACOVA_05554
	BT4704	BACOVA_05555
28	BT4705	BACOVA_05556
	BT4706	BACOVA_05558
	BT4707	BACOVA_05559
	BT4708	BACOVA_05560
	BT4709	BACOVA_05561
	BT4710	BACOVA_05562
	BT4712	BACOVA_05564
	BT4713	BACOVA_04535
	BT4714	BACOVA_05565
	BT4715	BACOVA_05566
	BT4716	BACOVA_05567
	BT4718	BACOVA_05568
	BT4719	BACOVA_05569
	BT4720	BACOVA_05570

	BT4721	BACOVA_05571
