# Supplementary material for: Recognition and Degradation of Plant Cell Wall Polysaccharides by Two Human Gut Symbionts
Source: PLoS Biol. 2011 Dec 20;9(12):e1001221. doi: 10.1371/journal.pbio.1001221 (PMC3243724; doi:10.1371/journal.pbio.1001221)
Supplement: Table S7 — Supplier information for carbohydrate growth array. (PDF) [file pbio.1001221.s016.pdf]

Table S7. Supplier information for carbohydrate growth array

Substrate	source	2x stock (mg/ml	sterilization
arabinose	Sigma A3131	10	FS
cellobiose	Sigma C7252	10	FS
fructose	Sigma F0127	10	FS
fucose	Sigma F2252	10	FS
galactose	Sigma G0750	10	FS
galacturonic acid	Fluka 73960	10	FS
glucosamine	Sigma G4875	10	FS
glucose	Sigma G8270	10	FS
glucuronic acid	Sigma G8645	10	FS
mannose	Sigma M4625	10	FS
N-acetylgalactosamine	Sigma A2795	10	FS
N-acetylglucosamine	Sigma A3286	10	FS
N-acetylneuraminic acid (IV-S)	Sigma A0812	10	FS
rhamnose	Sigma R3875	10	FS
xylose	Sigma X3877	10	FS
chondroitin sulfate (bovine trachea)	Sigma C9819	10	AC
heparin (porcine mucosa)	Sigma H0777	10	FS
hyaluronan (rooster comb)	Sigma H5388	10	AC
α-mannan (*S. cerevisiae*)	Sigma M7504	10	AC
neutral mucin O-glycans (porcine muco	custom (porcine gastric mucosa)	20	FS
lichenan	Megazyme	10	AC
rhamnogalacturonan II	custom (red wine)	15	AC
laminarin	Sigma L9634	10	AC
dextran (*L. mesenteroides*)	Sigma D5251	10	AC
arabinan (sugar beet)	Megazyme P-ARAB	10	AC
arabinogalactan (larch)	Megazyme P-ARGAL	10	AC
pectic galactan (potato)	Megazyme P-GAPT	10	AC
homogalacturonan (citrus peel)	Megazyme P-GACT	10	AC
rhamnogalacturonan I (potato pectin)	Megazyme P-RHAM1	20	AC
arabinoxylan (wheat)	Megazyme P-WAXYL	10	AC
β-glucan (barley)	Megazyme P-BGBL	10	AC
galactomannan (carob)	Megazyme P-GALML	10	AC
glucomannan (konjac)	Megazyme P-GLCML	10	AC
xylan, water soluble (oat spelt)	Fluka 95590	10	AC
xyloglucan (tamarind)	Megazyme P-XYGLN	10	AC
inulin (chicory)	Sigma I2255	10	AC
levan (*E. herbicola*)	Sigma L8647	10	AC
pullulan (*A. pullulans*)	Sigma P4516	10	AC
Glycogen	sigma G0885	10	AC
amylopectin (maize)	Sigma 10120	10	AC
amylopectin (potato)	Sigma 10118	10	AC

Carbohydrate sterilization methods were either by filter sterilization through a 0.22μ filter (FS);
or by autoclaving for 15 min. at 121 degrees C (AC).
