# Supplementary material for: Recognition and Degradation of Plant Cell Wall Polysaccharides by Two Human Gut Symbionts
Source: PLoS Biol. 2011 Dec 20;9(12):e1001221. doi: 10.1371/journal.pbio.1001221 (PMC3243724; doi:10.1371/journal.pbio.1001221)
Supplement: Table S8 — Microarray datasets used in this study. All GeneChip data used in this study are available from the Gene Expression Omnibus (GEO) database (www.ncbi.nlm.nih.gov/projects/geo/) under the indicated accession number. (PDF) [file pbio.1001221.s017.pdf]

Table S8. Microarray datasets used in this study

*Denotes substrate with high turbidity that prohibited accurate absorbance measurement. Where an OD_{600} range is given, values represent the range of absorbance values based on absolute reading (high value) minus total starting turbidity (low value). In some cases only the number of hours under which a culture was incubated is shown (*e.g.*, wheat arabinoxylan).

GEO series	GEO number	GEO platform	Species	Experiment description	Culture type/ volume	Growth phase/OD_{600}*
**New *in vitro* arrays for this study:**						
GSE25576	GSM628320	GLP7006	*Bt (wild-type)*	MM-pectic galactan (5mg/ml) #1	Tube/5ml	mid-log/0.53-0.79*
GSE25576	GSM628321	GLP7006	*Bt (wild-type)*	MM-pectic galactan (5mg/ml) #2	Tube/5ml	mid-log/0.53-0.79*
GSE25576	GSM628322	GLP7006	*Bt (wild-type)*	MM-homogalacturonan (5 mg/ml) #1	Tube/5ml	mid-log/0.62
GSE25576	GSM628323	GLP7006	*Bt (wild-type)*	MM-homogalacturonan (5 mg/ml) #2	Tube/5ml	mid-log/0.62
GSE25576	GSM628318	GLP7006	*Bt (wild-type)*	MM-arabinan (5 mg/ml) #1	Tube/5ml	mid-log/0.52-0.69*
GSE25576	GSM628319	GLP7006	*Bt (wild-type)*	MM-arabinan (5 mg/ml) #2	Tube/5ml	mid-log/0.49-0.66*
GSE25576	GSM628328	GLP7006	*Bt (wild-type)*	MM-arabinogalacatan (5mg/ml) #1	Tube/5ml	mid-log/0.59
GSE25576	GSM628329	GLP7006	*Bt (wild-type)*	MM-arabinogalacatan (5mg/ml) #2	Tube/5ml	mid-log/0.61
GSE25576	GSM628330	GLP7006	*Bt (wild-type)*	MM-pullulan (5 mg/ml) #1	Tube/5ml	mid-log/0.61
GSE25576	GSM628331	GLP7006	*Bt (wild-type)*	MM-pullulan (5 mg/ml) #2	Tube/5ml	mid-log/0.59
GSE25576	GSM628324	GLP7006	*Bt (wild-type)*	MM-1% rhamnogalacturonan I (10 mg/ml)	Tube/5ml	mid-log/0.52
GSE25576	GSM628325	GLP7006	*Bt (wild-type)*	MM-1% rhamnogalacturonan I (10 mg/ml)	Tube/5ml	mid-log/0.53
GSE25576	GSM628326	GLP7006	*Bt (wild-type)*	MM-1.5% rhamnogalacturonan II	Tube/5ml	mid-log/0.37
GSE25576	GSM628327	GLP7006	*Bt (wild-type)*	MM-1.5% rhamnogalacturonan II	Tube/5ml	mid-log/0.38
GSE25576	GSM628389	GLP9803	*Bo (wild-type)*	MM-glucose (5mg/ml) #1	Tube/5ml	mid-log/0.65
GSE25576	GSM628390	GLP9803	*Bo (wild-type)*	MM-glucose (5mg/ml) #2	Tube/5ml	mid-log/0.60
GSE25576	GSM628395	GLP9803	*Bo (wild-type)*	MM-xyloglucan (5mg/ml) #1	Tube/5ml	mid-log/0.64
GSE25576	GSM628396	GLP9803	*Bo (wild-type)*	MM-xyloglucan (5mg/ml) #2	Tube/5ml	mid-log/0.60
GSE25576	GSM628401	GLP9803	*Bo (wild-type)*	MM-beta glucan (5mg/ml) #1	Tube/5ml	mid-log/0.62
GSE25576	GSM628402	GLP9803	*Bo (wild-type)*	MM-beta glucan (5mg/ml) #2	Tube/5ml	mid-log/0.60
GSE25576	GSM628393	GLP9803	*Bo (wild-type)*	MM-wheat arabinoxylan (5mg/ml) #1	Tube/5ml	mid-log/12-hour
GSE25576	GSM628394	GLP9803	*Bo (wild-type)*	MM-wheat arabinoxylan (5mg/ml) #2	Tube/5ml	mid-log/12-hour
GSE25576	GSM628397	GLP9803	*Bo (wild-type)*	MM-glucomannan (5mg/ml) #1	Tube/5ml	mid-log/0.62
GSE25576	GSM628398	GLP9803	*Bo (wild-type)*	MM-glucomannan (5mg/ml) #2	Tube/5ml	mid-log/0.59
GSE25576	GSM628399	GLP9803	*Bo (wild-type)*	MM-galactomannan (5mg/ml) #1	Tube/5ml	mid-log/0.63
GSE25576	GSM628400	GLP9803	*Bo (wild-type)*	MM-galactomannan (5mg/ml) #2	Tube/5ml	mid-log/0.64
GSE25576	GSM628391	GLP9803	*Bo (wild-type)*	MM-oat spelt xylan (OSX) (5mg/ml) #1	Tube/5ml	mid-log/0.40 (*turbid substrate)
GSE25576	GSM628392	GLP9803	*Bo (wild-type)*	MM-oat spelt xylan (OSX) (5mg/ml) #2	Tube/5ml	mid-log/0.38 (*turbid substrate)
GSE25576	GSM628403	GLP9803	*Bo (wild-type)*	MM-homogalacturonan (5mg/ml) #1	Tube/5ml	mid-log/0.61
GSE25576	GSM628404	GLP9803	*Bo (wild-type)*	MM-homogalacturonan (5mg/ml) #2	Tube/5ml	mid-log/0.60

New *in vivo* arrays for this study:

GSE25576	GSM628405	GLP9803	*Bo (wild-type)*	*in vivo* NMRI cecal; plant rich diet #1	N/A	*in vivo*; NMRI cecum, plant rich diet 1/1
GSE25576	GSM628406	GLP9803	*Bo (wild-type)*	*in vivo* NMRI cecal; plant rich diet #2	N/A	*in vivo*; NMRI cecum, plant rich diet 1/2
GSE25576	GSM628407	GLP9803	*Bo (wild-type)*	*in vivo* NMRI cecal; plant rich diet #3	N/A	*in vivo*; NMRI cecum, plant rich diet 1/3

In vitro arrays included from previous studies:

GSE11980	GSM302686	GPL7006	*Bt* (wild-type)	Minimal medium plus glucose 0.5% (w/v); replicate 1/2	Tube/5ml	middle logarithmic growth phase/0.62
GSE11980	GSM302791	GPL7006	*Bt* (wild-type)	Minimal medium plus glucose 0.5% (w/v); replicate 2/2	Tube/5ml	middle logarithmic growth phase/0.64
GSE11980	GSM302800	GPL7006	*Bt* (wild-type)	Minimal medium plus chondroitin sulfate 0.5% (w/v); replicate 1/2	Tube/5ml	middle logarithmic growth phase/0.65
GSE11980	GSM302801	GPL7006	*Bt* (wild-type)	Minimal medium plus chondroitin sulfate 0.5% (w/v); replicate 2/2	Tube/5ml	middle logarithmic growth phase/0.68
GSE11980	GSM302802	GPL7006	*Bt* (wild-type)	Minimal medium plus hyaluronan 0.5% (w/v); replicate 1/2	Tube/5ml	middle logarithmic growth phase/0.67
GSE11980	GSM302803	GPL7006	*Bt* (wild-type)	Minimal medium plus hyaluronan 0.5% (w/v); replicate 2/2	Tube/5ml	middle logarithmic growth phase/0.62
GSE11980	GSM302804	GPL7006	*Bt* (wild-type)	Minimal medium plus heparin 0.5% (w/v); replicate 1/2	Tube/5ml	middle logarithmic growth phase/0.62
GSE11980	GSM302805	GPL7006	*Bt* (wild-type)	Minimal medium plus heparin 0.5% (w/v); replicate 2/2	Tube/5ml	middle logarithmic growth phase/0.55
GSE11980	GSM302806	GPL7006	*Bt* (wild-type)	Minimal medium plus α-mannan 0.5% (w/v); replicate 1/2	Tube/5ml	middle logarithmic growth phase/0.59
GSE11980	GSM302807	GPL7006	*Bt* (wild-type)	Minimal medium plus α-mannan 0.5% (w/v); replicate 2/2	Tube/5ml	middle logarithmic growth phase/0.60
GSE11980	GSM301720	GPL1821	*Bt* (wild-type)	Minimal medium plus glucose 0.5% (w/v); replicate 1/2	Tube/5ml	middle logarithmic growth phase/0.62
GSE11980	GSM301721	GPL1821	*Bt* (wild-type)	Minimal medium plus glucose 0.5% (w/v); replicate 2/2	Tube/5ml	middle logarithmic growth phase/0.64
GSE11980	GSM301722	GPL1821	*Bt* (wild-type)	Minimal medium plus 0.5% (w/v) neutral PMG glycans; replicate 1/2	Tube/5ml	middle logarithmic growth phase/0.59
GSE11980	GSM301731	GPL1821	*Bt* (wild-type)	Minimal medium plus 0.5% (w/v) neutral PMG glycans; replicate 2/2	Tube/5ml	middle logarithmic growth phase/0.63

In vivo arrays included from previous studies:

GSE2231	GSM40886	GPL1821	*Bt* (wild-type)	Cecal bacterial population from adult NMRI inbred mouse fed simple sugar diet;	N/A	N/A
GSE2231	GSM40887	GPL1821	*Bt* (wild-type)	Cecal bacterial population from adult NMRI inbred mouse fed simple sugar diet;	N/A	N/A
GSE2231	GSM40888	GPL1821	*Bt* (wild-type)	Cecal bacterial population from adult NMRI inbred mouse fed simple sugar diet;	N/A	N/A

GSE5279	GSM119522	GPL1821	*Bt* (wild-type)	Pooled cecal bacterial populations from 17 day-old NMRI inbred mice suckling on mother's milk; replicate 1/6	N/A	N/A
GSE5279	GSM119523	GPL1821	*Bt* (wild-type)	Pooled cecal bacterial populations from 17 day-old NMRI inbred mice suckling on mother's milk; replicate 2/6	N/A	N/A
GSE5279	GSM119524	GPL1821	*Bt* (wild-type)	Pooled cecal bacterial populations from 17 day-old NMRI inbred mice suckling on mother's milk; replicate 3/6	N/A	N/A
GSE5279	GSM119525	GPL1821	*Bt* (wild-type)	Pooled cecal bacterial populations from 17 day-old NMRI inbred mice suckling on mother's milk; replicate 4/6	N/A	N/A
GSE5279	GSM119526	GPL1821	*Bt* (wild-type)	Pooled cecal bacterial populations from 17 day-old NMRI inbred mice suckling on mother's milk; replicate 5/6	N/A	N/A
GSE5279	GSM119527	GPL1821	*Bt* (wild-type)	Pooled cecal bacterial populations from 17 day-old NMRI inbred mice suckling on mother's milk; replicate 6/6	N/A	N/A
GSE2231	GSM40892	GPL1821	*Bt* (wild-type)	Cecal bacterial population from adult NMRI inbred mouse fed plant rich diet; replicate	N/A	N/A
GSE2231	GSM40893	GPL1821	*Bt* (wild-type)	Cecal bacterial population from adult NMRI inbred mouse fed plant rich diet; replicate	N/A	N/A
GSE2231	GSM40894	GPL1821	*Bt* (wild-type)	Cecal bacterial population from adult NMRI inbred mouse fed plant rich diet; replicate	N/A	N/A
GSE2231	GSM40895	GPL1821	*Bt* (wild-type)	Cecal bacterial population from adult NMRI inbred mouse fed plant rich diet; replicate	N/A	N/A
GSE2231	GSM40896	GPL1821	*Bt* (wild-type)	Cecal bacterial population from adult NMRI inbred mouse fed plant rich diet; replicate	N/A	N/A
GSE2231	GSM40891	GPL1821	*Bt* (wild-type)	Cecal bacterial population from adult NMRI inbred mouse fed plant rich diet; replicate	N/A	N/A
GSE2231	GSM40889	GPL1821	*Bt* (wild-type)	Cecal bacterial population from adult NMRI inbred mouse fed plant rich diet; replicate	N/A	N/A
GSE2231	GSM40890	GPL1821	*Bt* (wild-type)	Cecal bacterial population from adult NMRI inbred mouse fed plant rich diet; replicate	N/A	N/A
GSE2231	GSM40885	GPL1821	*Bt* (wild-type)	Cecal bacterial population from adult NMRI inbred mouse fed plant rich diet; replicate	N/A	N/A
