# Supplementary material for: Recognition and Degradation of Plant Cell Wall Polysaccharides by Two Human Gut Symbionts
Source: PLoS Biol. 2011 Dec 20;9(12):e1001221. doi: 10.1371/journal.pbio.1001221 (PMC3243724; doi:10.1371/journal.pbio.1001221)
Supplement: Table S9 — Primers used in this study. Sequences recognized by restriction enzymes used for molecular cloning are underlined. (PDF) [file pbio.1001221.s018.pdf]

Table S9. Primers used in this study

Sequences recognized by restriction enzymes used for molecular cloning are underlined

Name	Species	Sequence(5'-3')
**HTCS mutants:**		
BT0267-1F	*Bt*	CCTTGAATTCGTACTGTTTTTATTTAATTATTTCCGTGTGC
BT0267-1R	*Bt*	CCTTGAATTCCCATTTTAATTTTCCGCAATACAAGTAATTT
BT0366-1F	*Bt*	CCTTGAATTCGACCGCTAAAATATTACGTATGTGACCATG
BT0366-1R	*Bt*	CCTTGAATTCGTAGTTACAGTATCTTTGGAAGTATCCCTTT
BT0958-1F	*Bt*	CCTTGAATTCTACTAACATATTGGAGTAGAGGATGGTTTG
BT0958-1R	*Bt*	CCTTGAATTCAACTAACCGTAACTGATGGCATCAGTGA
BT0981-1F	*Bt*	CCTTGAATTCACGGTACCTAATTCCGTTCCTATGATAA
BT0981-1R	*Bt*	CCTTGAATTCAAAACACATTATGCTAGAGGCGTAAGTC
BT1183-2F	*Bt*	CCTTGAATTCCTAAACCTATCTGGGCTACGGTATCG
BT1183-2R	*Bt*	CCTTGAATTCGCCGTTTCGGCTGGAAGTAGA
BT1635-2F	*Bt*	CCTTGAATTCACTAACTGAAGAAATACATCCATCATGC
BT1635-2R	*Bt*	CCTTGAATTCGTCCCGGTAGCTGTAAAAGTAAATTTTG
BT1734-1F	*Bt*	CCTTGAATTCTTAAGCAACCGGTGAGTGTACTACATTCAA
BT1734-1R	*Bt*	CCTTGAATTCAAGTAATGGGAGCCGGTTAATCCTTAAA
BT1754-1F	*Bt*	CCTTGAATTCCATGGTGACATAAGATGAATGATGAGAT
BT1754-1R	*Bt*	CCTTGAATTCCTACCGAACGACCGATTTAATAATTATC
BT2391-1F	*Bt*	CCTTGAATTCTGAACTACATTAGTTGAATATGAGTAATGGC
BT2391-1R	*Bt*	CCTTGAATTCCCCTGTTATTTACATAGCACTTACAGAAA
BT2628-1F	*Bt*	CCTTGAATTCTATGCTTAAAATTTGAAGCAAGTTGCAGAT
BT2628-1R	*Bt*	CCTTGAATTCGCCATGACTATCCTAATTCATAAAGAAGAGTT
BT2860-1F	*Bt*	CCTTGAATTCACTGCTAAGATCATCAGTTAAAGAAAGG
BT2860-1R	*Bt*	CCTTGAATTCCCTGCTTAAATTGTCACCTATTGCTGAT
BT2897-1F	*Bt*	CCTTGAATTCGAGGGTTAGGCTTTTCGTTCACTGGATATA
BT2897-1R	*Bt*	CCTTGAATTCTCGATCCCTGTATCTAAATCACTTTACATTA
BT2923-1F	*Bt*	CCTTGAATTCATTCGTAAGGCTTTATGTGGTTTGGTAC
BT2923-1R	*Bt*	CCTTGAATTCCCGCAAACTTTGTTCTTTTAAGTCTTAACA
BT3049-1F	*Bt*	CCTTGAATTCATCCAATGATTGGGAATAGAACAAGGCT

BT3049-1R	*Bt*	CCTTGAATTCCGGATTTTATTGGTAGACGCTGAAACTT
BT3097-1F	*Bt*	CCTTGAATTCTATCTTAGGACAGTAAGGGATTTATGTGGT
BT3097-1R	*Bt*	CCTTGAATTCTATCGTATTAATAGATGCCGTTACTGCA
BT3172-1F	*Bt*	CCTTGAATTCGACGGACACTAACTGAAGAGATACAAAC
BT3172-1R	*Bt*	CCTTGAATTCCATTGAAATATTGAAGGAGTCACAGCGA
BT3302-2F	*Bt*	CCTTGAATTCTGATATGAGTAGACATAAATGCTTCGTATA
BT3302-2R	*Bt*	CCTTGAATTCTGTATTTAAAGTCTTTGAAGCTTTGTATGG
BT3334-1F	*Bt*	CCTTGAATTCGTTATGACAAAATATTACTTTCAGCCATTTG
BT3334-1R	*Bt*	CCTTGAATTCTTTTAGTTGTAGTAGATAGCGTCCACATTG
BT3465-2F	*Bt*	CCTTGAATTCTATGATGTGCTATACAGGATAGCTATGGATTTA
BT3465-2R	*Bt*	CCTTGAATTCATCATAAATAAAAAGTCCCTGCCCCAAG
BT3678-1F	*Bt*	CCTTGAATTCAATAGCATCACTTGTTAGGTTGATGATAATAATAA
BT3678-1R	*Bt*	CCTTGAATTCACTTTATCTGTGGATGGAGATTAACGAAAT
BT3738-1F	*Bt*	CCTTGAATTCTTTATTAGTACATAGGGTTGGAAGACGGTT
BT3738-1R	*Bt*	CCTTGAATTCTCAATTAAGGGTGTACATAAGCCAATTC
BT3786-1F	*Bt*	CCTTGAATTCTCTGTAGCAGGTTGCTGACAAAGAATAT
BT3786-1R	*Bt*	CCTTGAATTCGGTGCTATGATTATCCTAATTCATCAAGAAG
BT4111-1F	*Bt*	CCTTGAATTCATTGAATCAATCGTTTTGATGGCTATAACT
BT4111-1R	*Bt*	CCTTGAATTCTGATTACGTCGTATAGTTCTTCTGAGGAAG
BT4124-1F	*Bt*	CCTTGAATTCGTCAATGACTGAATGTCTCAAGGAATAGTA
BT4124-1R	*Bt*	CCTTGAATTCGCTTAATTATTCGTTATCATCGTTTCTGC
BT4137-1F	*Bt*	CCTTGAATTCTTACTAGAAACAAATCTCATTAAAGGCTGGA
BT4137-1R	*Bt*	CCTTGAATTCATCTAATTTTCACCTTTCTCATCCGTCT
BT4178-1F	*Bt*	CCTTGAATTCATTTAACTCCGTTCCAAACAAATGAGAA
BT4178-1R	*Bt*	CCTTGAATTCTTTAGTCTACAAAACAGGCACGTTGCAAGT
BT4182-1F	*Bt*	CCTTGAATTCATAATTGAATCCGATATATGTTTCAGGACAG
BT4182-1R	*Bt*	CCTTGAATTCAGATAGACTAGTCGGATGCAATGATTTT
BT4236-1F	*Bt*	CCTTGAATTCGCAAATAATTTAAAACGGAGTCGGAAAT
BT4236-1R	*Bt*	CCTTGAATTCTTAAGTTACAGCGACCTGTCAAAACGTA
BT4663-1F	*Bt*	CCTTGAATTCGGAATAAATCACATTTTCCTATATTTCCA
BT4663-1R	*Bt*	CCTTGAATTCCCATTCTATTCATAGAAGAAGTTCTGGAAT

BT4673-1F	*Bt*	gcggtcgactactttccgtcaaccgga
BT4673-1R	*Bt*	gcgtctagagagattggaataaactgcagcg

HTCS periplasmic domain expression:

BT0366 NcoF	*Bt*	CTCCCATGGGCCAGGAACGGTTTGCCGACCGCTAT
BT0366 XhoR	*Bt*	CTCCTCGAGAGTCTTATAGAAATAAGGTACAAT
Bacova_02097 NheF	*Bo*	CTCGCTAGCAGTTTTCACCAGCTGGGAGTAAAA
Bacova_02097 XhoR	*Bo*	CTCCTCGAGGAATGGCGGATACACATTGATGGA
Bacova_02740 NcoF	*Bo*	CTCCCATGGGCGAACTGACCAACCGGATGTTCGAT
Bacova_02740 XhoR	*Bo*	CTCCTCGAGCTGGCTCACTTTCACTTCTAAGGTGCT
Bacova_04394 NcoF	*Bo*	CTCCCATGGGCCAGACCGGCAAATTCTATTCAACA
Bacova_04394 XhoR	*Bo*	CTCCTCGAGGGTCTGGTACCAGGGTGGAGTGAT
BT3049 NcoF	*Bt*	ctcccatggctgcccacccttacctcatccaaagattg
BT3049 XhoR	*Bt*	ctcctcgagtgtcagccagagaggagggttgatatgtat
BT4178 NcoF	*Bt*	ctcccatgggcggcggcattgaactccgttccaaa
BT4178 XhoR	*Bt*	ctcctcgaggttcgtcttccagaaaggaggcac
BT4182 NheF	*Bt*	ctcgctagctctgtcgagatacgttcgaacaaa
BT4182 XhoR	*Bt*	ctcctcgagggcccaggtcgtctgccagaaagg
BT4673 NcoF	*Bt*	ctcccatgggcaaaaaagtaaattatcagcaattcgac
BT4673 XhoR	*Bt*	ctcctcgagcggtggaagaatccggatattcagaatatg
Bacova_03437 NheF	*Bo*	ctcgctagcCaatctcctaagttattcactactgac
Bacova_03437 XhoR	*Bo*	ctcctcgagttaAgcataccatgcaggagatataaagat
Bacova_03441 NcoF	*Bo*	ctcccatgggcgagaatacattgttctatgattcc
Bacova_03441 XhoR	*Bo*	ctcctcgagagacgaacgccaccacggtgc

qPCR:

Bacova_02096 F	*Bo*	GCACTTGTTCGTTTTGATAATTGGCAT
Bacova_02096 R	*Bo*	AAGGGAAATGAAAAGGTGGAATTCAG

Bacova_02652 F	*Bo*	ACGGATATAACGTGATAGGCATGTT
Bacova_02652 R	*Bo*	TCACACGCTCACCATTTTTATCGAG
Bacova_02742 F	*Bo*	TTGGTTTCAAGTCTTTGTTGGGGAT
Bacova_02742 R	*Bo*	CCGATCCTTATCAATTGGGACTCG
Bacova_03426 F	*Bo*	CCCTTCCATTCAGCTCCAATTG
Bacova_03426 R	*Bo*	GCGACGGTGTAATTGATGCTGATGA
Bacova_03428 F	*Bo*	CATCTCCATATACTCACGTTCTTTTGC
Bacova_03428 R	*Bo*	CGGAACTTTCATACGTGGACTTAGC
Bacova_04393 F	*Bo*	ATTGTTTCGCCTGTCTGATTACTCT
Bacova_04393 R	*Bo*	ATGAACCGTAATAAAGTAAAATCGCTGGA
BT0362 F	*Bt*	TGTCTACTTCCTTCAGTGACCTCG
BT0362 R	*Bt*	AATCGTGCAGGAAGTGAGTAGC
BT0364 F	*Bt*	GAGAATCAGCAACGAAGCTTTCATGA
BT0364 R	*Bt*	ACTTTGTATACATCGTTACCTACTTCACCA
BT3046 F	*Bt*	CCAATGCACGTTATTATGTAAGTATGGGTAT
BT3046 R	*Bt*	ATGTTTACTTACCGACTTATCTTGTTTGAA
BT4164 F	*Bt*	GTTCATATAGTCTGTTCCTTTCACGTAGG
BT4164 R	*Bt*	GACGAAGCGAAATTCCGTTTCAG
BT4671 F	*Bt*	ATGGTTTCATCGCCCGAAGAG
BT4671 R	*Bt*	GCGTATGGTTGAGACAGATGTAGG
16S F	*Bt/Bo*	GGTAGTCCACACAGTAAACGATGAA
16S R	*Bt/Bo*	CCCGTCAATTCCTTTGAGTTTC
